# Dibismuthanes in catalysis: from synthesis and characterization to redox behavior towards oxidative cleavage of 1,2-diols†

**DOI:** 10.1039/d1ob00367d

**Published:** 2021-05-06

**Authors:** Marc Magre, Jennifer Kuziola, Nils Nöthling, Josep Cornella

**Affiliations:** Max-Planck-Institut für Kohlenforschung Mülheim an der Ruhr 45470 Germany cornella@kofo.mpg.de

## Abstract

A family of aryl dinuclear bismuthane complexes has been successfully synthesized and characterized. The two bismuth centers are bonded to various xanthene-type backbones, which differ in ring-size and flexibility, resulting in complexes with different intramolecular Bi⋯Bi distances. Moreover, their pentavalent Bi(v) analogues have also been prepared and structurally characterized. Finally, the synergy between bismuth centers in catalysis has been studied by applying dinuclear bismuthanes **5–8** to the catalytic oxidative cleavage of 1,2-diols. Unfortunately, no synergistic effects were observed and the catalytic activities of dinuclear bismuthanes and triphenylbismuth are comparable.

## Abstract 2

A family of novel dimetallic Bi(iii) and Bi(v) complexes is reported. Structural analysis of the complexes in the solid state permitted the study of the Bi–Bi distance and its impact in the catalytic redox properties.

## Graphical abstract

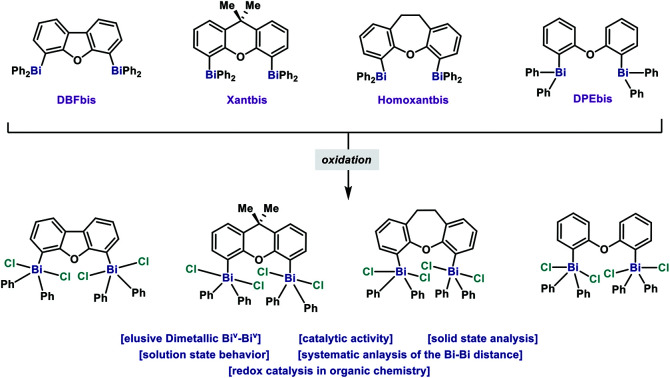

## Introduction

**Fig. 1:**
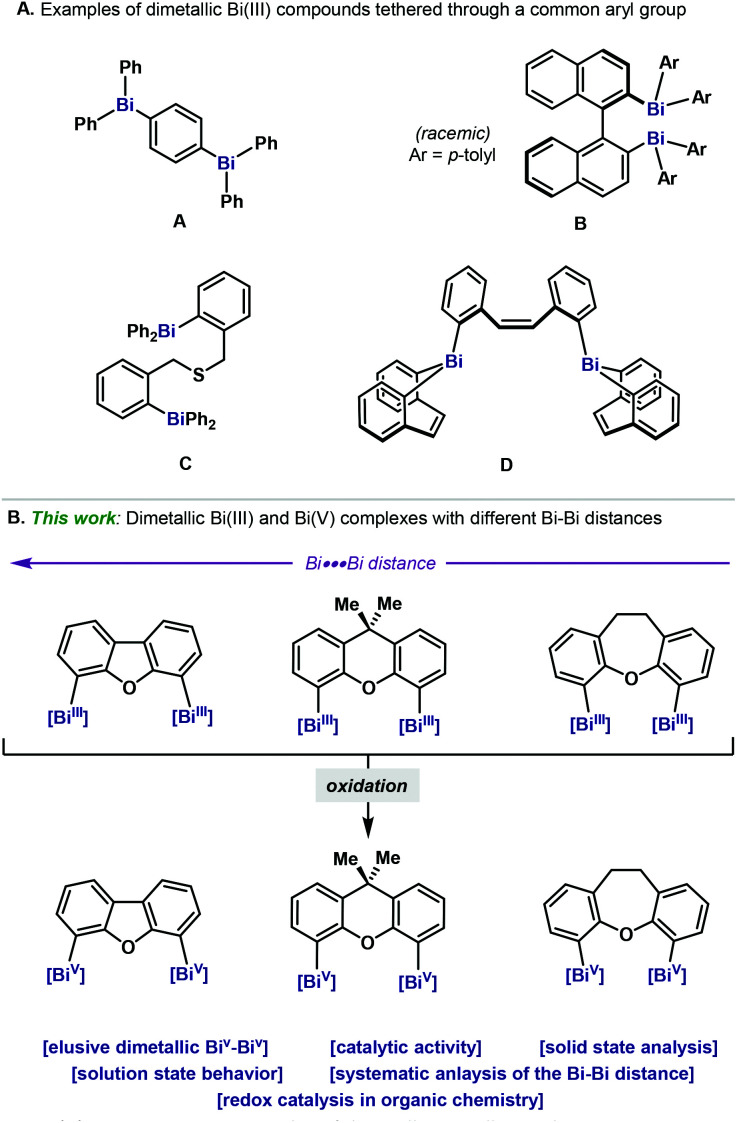
(A) Representative examples of dimetallic triarylbismuthanes. (B) Synthesis of dimetallic Bi(iii) and Bi(v) complexes.

Organobismuthanes are a class of organometallic reagents where the Bi(iii) is connected to C atoms. Such compounds have been largely studied and a wide variety of examples have been reported.^1^ In particular, the triarylbismuthanes subclass has attracted the attention of chemists due to their rather high stability and facile preparation. As a result, several monometallic organobismuthanes have been explored as reagents for organic synthesis.^2^ Compared to the vast literature on monometallic triarylbismuthanes, examples of bi- and dimetallic Bi complexes are much rarer and are mainly relegated to the low-valent counterparts Bi(i) and Bi(ii). Dimerization in these complexes is highly favored as a result of the unpaired electron in Bi(ii)^3,4^ or the stabilization of the highly reactive lone-pair in Bi(i).^3,5^ On the other hand, examples of dimetallic organobismuth complexes have been comparatively much less explored, and only a handful of examples exist in the literature.^6^ Indeed, compared to the lighter counterparts in the group 15 (*e.g.* N^7^ and P^8^), examples of dimetallic heavy pnictogens are really limited and mainly focus on As and Sb,^9,10^ leaving dimetallic Bi compounds as boutique examples (Fig. 1A).^6^ This can be attributed to the classical ligand redistribution of heteroleptic triarylbismuthanes,^11^ which poses severe hurdles in the selective synthesis of unsymmetrical dinuclear bismuthanes. Among the known examples, a common denominator is the high ligand flexibility and the long distances between the two Bi atoms (Fig. 1A). Yet, access to dibismuthanes bearing a more rigid ligand backbone would enable a systematic study of the Bi–Bi distance. Herein we report the synthesis, characterization and structural analysis of a family of new triaryl dinuclear bismuthanes, which bear distinct aromatic backbones on the tether unit. Systematic structural variations on the backbone permit an evaluation on how the rigidity of the tether influences the Bi–Bi distance (Fig. 1B).

Oxidation of the dibismuthanes with SO_2_Cl_2_ afforded high yields of dimetallic Bi(v) dichloro compounds, a class of compounds that remained elusive to date. The structure of both Bi(iii) and Bi(v) dimetallic complexes was elucidated by single crystal X-ray diffraction and their behavior in solution analyzed by NMR. Due to our growing interest in exploring the redox properties of Bi,^12^ the catalytic redox properties of these compounds have also been explored in the context of the oxidative cleavage of 1,2-diols.

## Results and discussion

### Synthesis of heteroleptic triaryldibismuthanes **5–8**

**Scheme 1:**
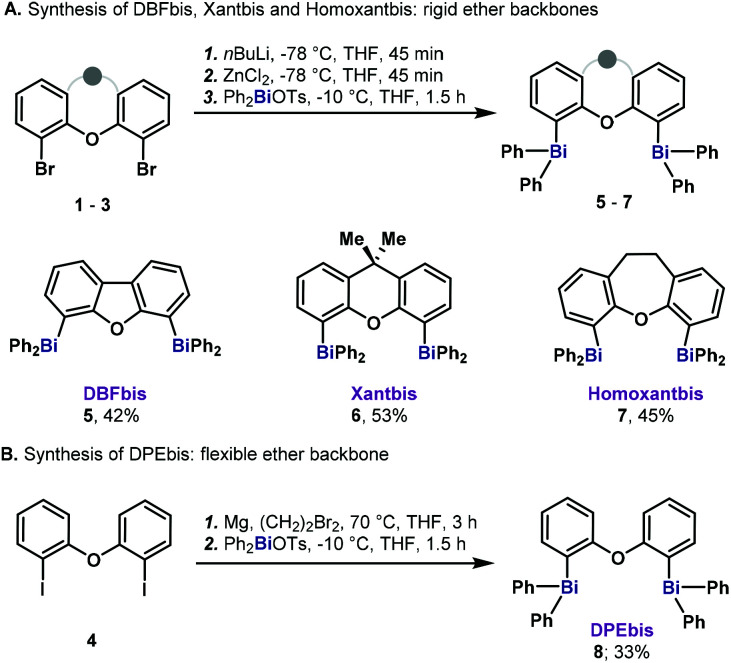
Synthesis of dibismuthanes **5–8**.

Based on the precedents in the synthesis of diphosphines and the availability of methods to obtain dihalogenated precursors,^13^ we set out to explore the heavy analogs of Xantphos, Homoxantphos, DBFphos and DPEphos, where the P atom has been replaced by Bi. Capitalizing on the important work from Hyvl on the efficient synthesis of heteroleptic triarylbismuthanes,^14^ we were able to access the desired dibismuthanes (**5–8**) in good yields (Scheme 1). Dinuclear bismuthanes bearing a rigid xanthene-like backbone (**5–7**) could be isolated in good yields as white solids *via* an organozinc-transmetalation protocol (Scheme 1A). On the other hand, dibismuthane **8**, bearing a more flexible backbone, required *in situ* Grignard formation followed by transmetalation to Ph_2_BiOTs (Scheme 1B). It is important to mention that **5–8** were reluctant to hydrolysis and were purified by flash chromatography through silica gel. Crystals suitable for X-ray diffraction were obtained for all the compounds prepared. By analogy to the P counterparts and based on the already reported nomenclature for BINABi,^6*b*^ we named the new dibismuthanes Xantbis, Homoxantbis, DPEbis and DBFbis.

With dibismuthanes **5–8** in hand, we can structurally compare the effect of the ring size of the backbone (**5***vs.***6** and **7**) and the flexibility (**5***vs.***8**). Dibismuthanes **5–8** are stable towards air and moisture, both in solution and in solid state. By means of NMR spectroscopy, we could observe that dinuclear bismuthanes **5–8** present a *C*_2_-symmetric backbone in solution, which suggest both Bi atoms are equivalent.

**Fig. 2:**
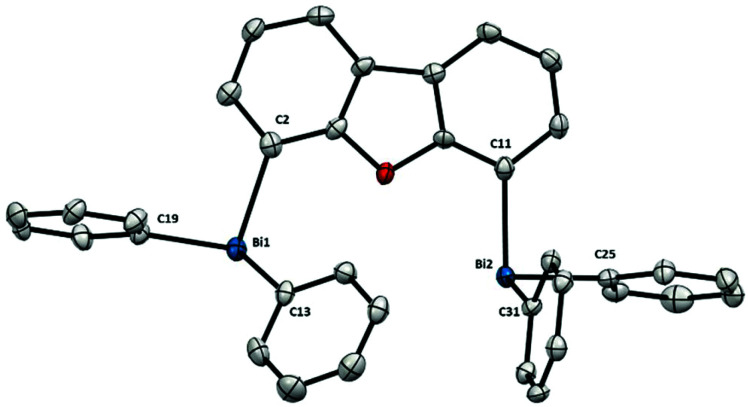
Structure of **5**. Ellipsoids are drawn at the 50% probability level and H atoms are omitted for clarity. Selected bond lengths (Å) and angles (°): Bi1–C2: 2.262(7), Bi1–C13: 2.244(6), Bi1–C19: 2.253(7), Bi2–C11: 2.252(7), Bi2–C25: 2.243(7), Bi2–C31: 2.248(7), C2–Bi1–C13: 93.7(2), C2–Bi1–C19: 96.3(3), C13–Bi1–C19: 93.8(2), C11–Bi2–C25: 95.8(2), C11–Bi2–C31: 94.0(2), C25–Bi2–C31: 96.4(2); Bi1⋯Bi2: 5.544(1) Å.

The solid state structure of dibismuthane **5** (Fig. 2) bears a close resemblance to the monometallic triphenylbismuth, both in the Bi–C distances and the trigonal pyramidal geometry around the Bi atoms.^15^ The resemblance in structure between triphenylbismuth and **5** is attributed to the rigidity of the backbone in **5**, which limits the torsion and results in a large Bi⋯Bi distance of 5.544(1) Å. This led us to speculate that both Bi in **5** do not interact with each other, and the complex can be conceived as two independent Bi centers, both electronically and structurally. However, as the Bi get closer in space, this resemblance to the monometallic Ph_3_Bi slowly disappears.

**Fig. 3:**
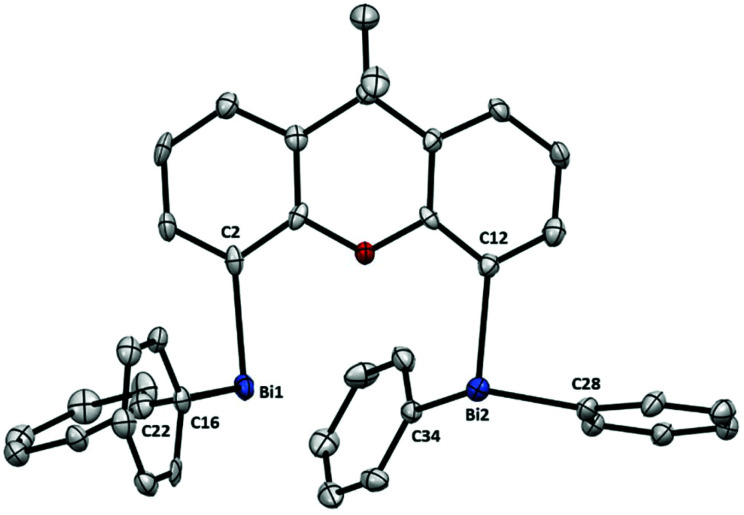
Structure of **6**. Ellipsoids are drawn at the 50% probability level and H atoms are omitted for clarity. Selected bond lengths (Å) and angles (°): Bi1–C2: 2.268(6), Bi1–C16: 2.246(5), Bi1–C22: 2.262(5), Bi2–C12: 2.255(5), Bi2–C28: 2.242(5), Bi2–C34: 2.252(5), C2–Bi1–C16: 96.16(19), C2–Bi1–C22: 93.60(19), C16–Bi1–C22: 93.82(18), C12–Bi2–C28: 90.36(19), C12–Bi2–C34: 94.80(2), C28–Bi2–C34: 93.06(19); Bi1⋯Bi2: 4.187(1) Å.

For example, the solid state structure of dibismuthane **6** (Fig. 3) shows the expected trigonal pyramidal geometry at both Bi atoms. However, compared to the geometry of triphenylbismuth,^15^ dinuclear bismuthane **6** shows a slight distortion comparing both Bi atoms [C2–Bi1–C16: 96.16(19), C2–Bi1–C22: 93.60(19), C16–Bi1–C22: 93.82(18) compared to C12–Bi2–C28: 90.36(19), C12–Bi2–C34: 94.80(2), C28–Bi2–C34: 93.06(19)], probably as a result of the steric hindrance between both centers. A closer look at the xanthene backbone revealed the characteristic bending, comparable to that observed for the phosphine analogue Xantphos.^16^ The structure of dibismuthane **6** shows a distance between both bismuth atoms of 4.187(1) Å, longer than its phosphorus homonym (4.045(1) Å).^16*b*^ In terms of Bi–C distances, **6** compares well with its monometallic analogue, showing that the presence of two bismuth units does not have a detrimental effect on the Bi–C distances.

**Fig. 4:**
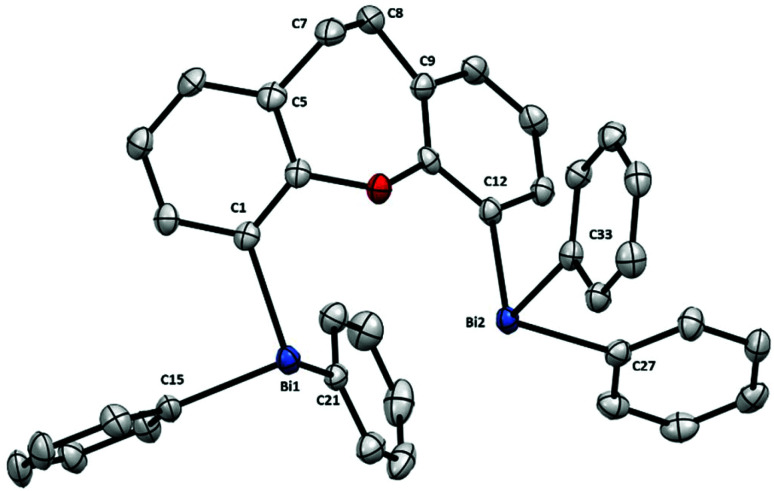
Structure of **7**. Ellipsoids are drawn at the 50% probability level and H atoms are omitted for clarity. Selected bond lengths (Å) and angles (°): Bi1–C1: 2.251(3), Bi1–C15: 2.255(3), Bi1–C21: 2.247(4), Bi2–C11: 2.252(3), Bi2–C27: 2.259(4), Bi2–C33: 2.262(3), C1–Bi1–C15: 92.32(12), C1–Bi1–C21: 97.62(12), C15–Bi1–C21: 94.52(13), C11–Bi2–C27: 92.72(12), C11–Bi2–C33: 93.21(12), C27–Bi2–C33: 94.34(12), C5–C7–C8: 108.8(3), C9–C8–C7: 115.0(3); Bi1⋯Bi2: 3.807(1) Å.

Differences from the monometallic triarylbismuth are more exacerbated in dinuclear bismuthane **7** (Fig. 4).

The presence of the BiPh_2_-moieties in **7** influences dramatically the backbone, increasing its torsion [C5–C7–C8: 108.8(3)°, C9–C8–C7: 115.0(3)° of **7**] compared to non-substituted benzoxepine derivative [C5–C7–C8: 113.54°, C9–C8–C7: 118.20°].^17^ We attribute this high structural torsion to the steric constraint between both bismuth centers, resulting in a longer C1–C12 distance [4.324(1) Å of **7***vs.* 4.039 Å of benzoxepine derivative]. However, the most remarkable feature in **7** is a Bi⋯Bi distance of 3.807(1) Å: much below the sum of the van der Waal radius (4.14 Å) and among the shortest distances between two Bi(iii) atoms reported.^18^ Additionally, examples of short Bi⋯Bi distances between two Bi(iii) atoms can be found in an intermolecular fashion in some diarylbismuth–(iii) halides [3.965(4)^18*a*^ and 3.973(9) Å^18*b*^] and also in BiMe_3_ [3.899(1) Å].^18*c*^

The increasing differences when moving from **5** to **7** are ascribed mainly due to the steric repulsion between the Bi atoms, which translates into rather unique torsions of the shared ligand backbones.

**Fig. 5:**
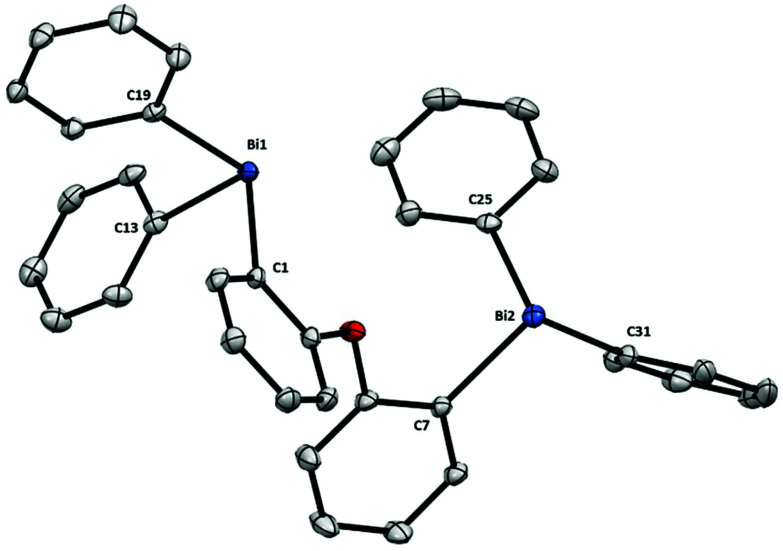
Structure of **8**. Ellipsoids are drawn at the 50% probability level and H atoms are omitted for clarity. Selected bond lengths (Å) and angles (°): Bi1–C1: 2.2624(17), Bi1–C13: 2.2494(19), Bi1–C19: 2.2605(18), Bi2–C7: 2.2426(18), Bi2–C25: 2.2491(18), Bi2–C31: 2.2525(19), C1–Bi1–C13: 97.61(6), C1–Bi1–C19: 92.13(6), C13–Bi1–C19: 92.20(7), C7–Bi2–C25: 94.66(7), C7–Bi2–C31: 97.14(7), C25–Bi2–C31: 91.50(7); Bi1⋯Bi2: 5.430(1) Å.

Finally, the structure of dibismuthane **8** (Fig. 5) shows that when the ether backbone is not tethered, the Bi–C remain unaltered and are comparable to those from dibismuthanes **5–7**. In the solid state structure the two aryl groups from the ligand backbone are placed almost perpendicular to each other [angle between phenyl planes 72.05(1)°], probably due to the steric hindrance of the –BiPh_2_ moieties. In fact, this structural behavior results in a Bi⋯Bi distance of 5.430(1) Å. Similarly as observed with **5** and **6** (compared to their corresponding P-analogues), the Bi⋯Bi distance in **8** is larger than its P-analog DPEphos (4.876 Å).^19^

### Synthesis of pentavalent dibismuth(v) **9–12**

**Scheme 2:**
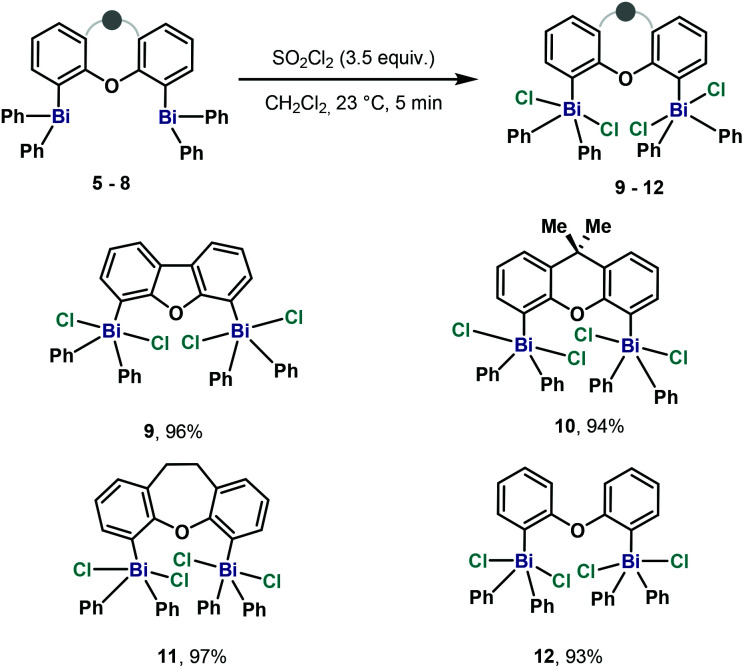
Synthesis of pentavalent dibismuth(v) compounds **9–12**.

After the successful synthesis and analysis in the solid state of a family of structurally different dibismuthanes **5–8**, we decided to study their reactivity towards oxidation. Whereas the oxidation of triarylbismuthanes has been widely studied using strong oxidants,^20,21^ it is worth mentioning that oxidation of dinuclear bismuthanes have not been reported. Then, we decided to oxidize **5–8** with the commonly utilized SO_2_Cl_2_. In all cases, smooth conversion of all dibismuthanes to the parent pentavalent dimetallic **9–12** were obtained (Scheme 2). Due to the high yields obtained, evaporation of the solvent led to pure Bi(v) compounds, whose structures were elucidated by single crystal X-ray analysis.

**Fig. 6:**
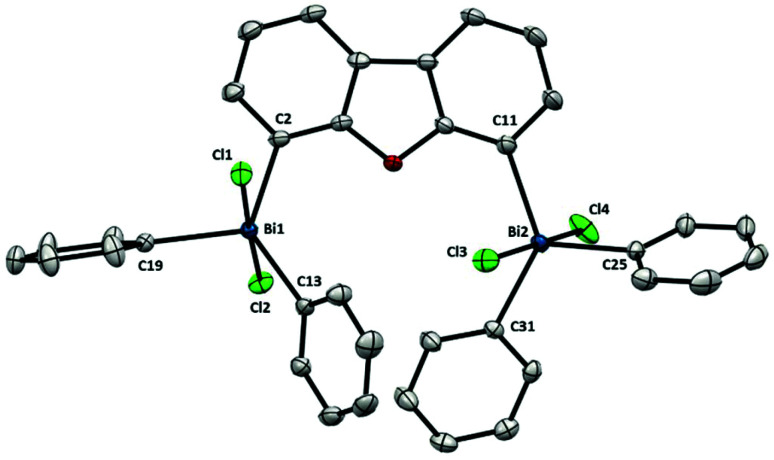
Structure of **9**. Ellipsoids are drawn at the 50% probability level and H atoms are omitted for clarity. Selected bond lengths (Å) and angles (°): Bi1–C2: 2.184(2), Bi1–C13: 2.213(2), Bi1–C19: 2.213(2), Bi1–Cl1: 2.5816(6), Bi1–Cl2: 2.5827(6), Bi2–C11: 2.186(3), Bi2–C25: 2.197(3), Bi2–C31: 2.194(2), Bi2–Cl3: 2.5496(6), Bi2–Cl4: 2.5769(6), C2–Bi1–C13: 130.24(9), C2–Bi1–C19: 113.08(9), C13–Bi1–C19: 116.51(8), Cl1–Bi1–Cl2; 177.46(2), C2–Bi1–Cl1: 86.64(7), C2–Bi1–Cl2: 91.17(7), C13–Bi1–Cl1: 89.26(7), C13–Bi1–Cl2: 91.19(7), C19–Bi1–Cl1: 90.29(7), C19–Bi1–Cl2: 91.75(7), C11–Bi2–C25: 112.86(9), C11–Bi2–C31: 128.63(9), C25–Bi2–C31: 118.32(9), Cl3–Bi2–Cl4: 174.32(2), C11–Bi2–Cl3: 92.41(7), C11–Bi2–Cl4: 87.53(7), C25–Bi2–Cl3: 93.52(7), C25–Bi2–Cl4: 91.74(7), C31–Bi2–Cl3: 88.73(6), C31–Bi2–Cl4: 86.88(6); Bi1⋯Bi2: 6.290(1) Å.

As depicted in Fig. 6, each Bi atom in **9** adopts a trigonal bipyramidal geometry with the two chloride ligands in apical position and the aromatic rings in equatorial, in agreement with the apicophilicity of electronegative ligands.^22^ The structure of pentavalent dibismuthane **9** bears a close resemblance to Ph_3_BiCl_2_.^23^ Due to the larger distance between both bismuth centers (Bi⋯Bi: 6.290(1) Å) the steric pressure between them is highly diminished. This structural feature is reflected on the distances between the Bi and the C in the backbone [Bi1–C2: 2.184(2) Å and Bi2–C11: 2.186(3) Å] showing minimal differences among them and to Ph_3_BiCl_2_ (Fig. 6).

**Fig. 7:**
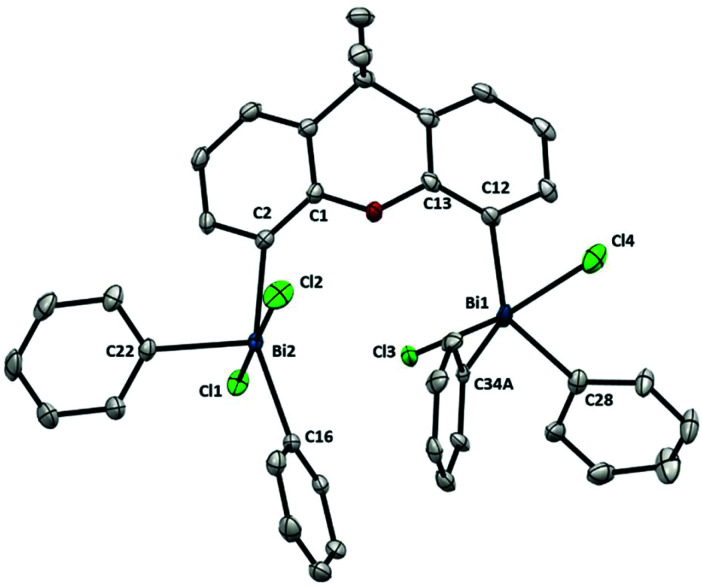
Structure of **10**. Ellipsoids are drawn at the 50% probability level and disordered parts, solvent and H atoms are omitted for clarity. Selected bond lengths (Å) and angles (°): Bi2–C2: 2.212(3), Bi2–C16: 2.223(2), Bi2–C22: 2.212(2), Bi2–Cl2: 2.5702(7), Bi2–Cl1: 2.5977(6), Bi1–C12: 2.190(2), Bi1–C34A: 2.189(5), Bi1–C28: 2.214(3), Bi1–Cl4: 2.5825(7), Bi1–Cl3: 2.5828(7), C2–Bi2–C16: 155.63(9), C2–Bi2–C22: 102.11(9), C16–Bi2–C22: 102.17(9), Cl1–Bi2–Cl2; 176.01(2), C2–Bi2–Cl2: 89.03(7), C2–Bi2–Cl1: 87.53(7), C16–Bi2–Cl2: 92.14(7), C16–Bi2–Cl1: 90.16(7), C22–Bi2–Cl2: 92.19(7), C22–Bi2–Cl1: 90.51(7), C12–Bi1–C34A: 137.74(16), C12–Bi1–C28: 116.05(10), C34A –Bi1–C28: 106.19(16), Cl3–Bi1–Cl4: 172.07(2), C12–Bi1–Cl4: 85.19(7), C12–Bi1–Cl3: 87.13(7), C34A –Bi1–Cl4: 93.92(16), C34A –Bi1–Cl3: 90.43(15), C28–Bi1–Cl4: 93.43(7), C28–Bi1–Cl3: 91.72(7); Bi1⋯Bi2: 5.031(1) Å.

**Scheme 3:**
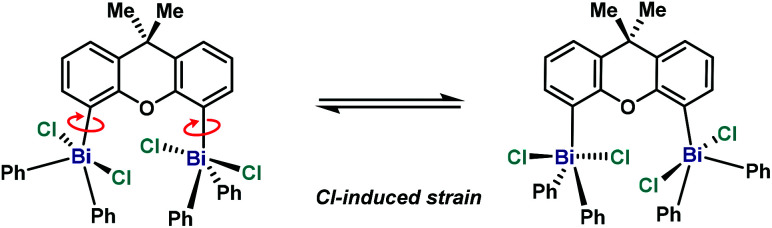
Proposed structural distortion observed in **10** at low temperature.

Compared to Ph_3_BiCl_2_,^23^ the geometry of complex **10** (Fig. 7) is slightly more distorted, probably due the steric hindrance between both Bi units. This geometry distortion is more evident when comparing both Bi centers. Whereas Bi1 atom adopts a geometry similar to the Ph_3_BiCl_2_ [C12–Bi1–C34A: 137.74(16)°, C12–Bi1–C28: 116.05(10)°, C34A–Bi1–28: 106.19(16)°], the geometry of Bi2 atom presents a larger degree of distorsion [C2–Bi2–C16: 155.63(9)°, C2–Bi2–C22: 102.11(9)°, C16–Bi2–C22: 102.17(9)°]. The large angle observed between C2–Bi2–C16 is the consequence of the effort of Bi2 to accommodate the incoming Cl3 from the neighboring Bi1. The steric hindrance between both Bi centers is also manifested in the distance between the Bi and the carbon at the backbone. For example, whereas Bi1–C12 distance is 2.190(2) Å, the distance of Bi2–C2 is elongated up to 2.212(3) Å. This repulsion is also reflected in the torsion between the Bi2 and the ligand backbone. For example, the C1–C2–Bi2 angle is 128.98(18)°, compared to the C13–C12–Bi1 angle of 124.27(19)°. Hence, the Bi2-center is out-of-plane due to the steric interaction with the neighboring Bi1. The high repulsion and steric hindrance in **10** results in the increase of the Bi⋯Bi distance (5.031(1) Å *vs.* 4.187(1) Å from the trivalent analogue **6**). Further insights into the repulsion and torsion between both Bi centers in **10** can be obtained from the VT-NMR analysis (from 25 °C to −90 °C, see ESI† for details). In solution at ambient temperature, the fluxionality between both bismuth centers is apparent, as **10** reveals itself as a *C*_2_-symmetric compound, with both Bi being equivalent. However, at lower temperatures, the two Bi atoms are no longer equivalent, consistent with the solid-state analysis of **10**. The solution behavior of **10** can be explained by a chloride-induced steric repulsion between the two TBP bismuth centers (Scheme 3). At low temperature, the Cl-induced structural strain can be observed.

**Fig. 8:**
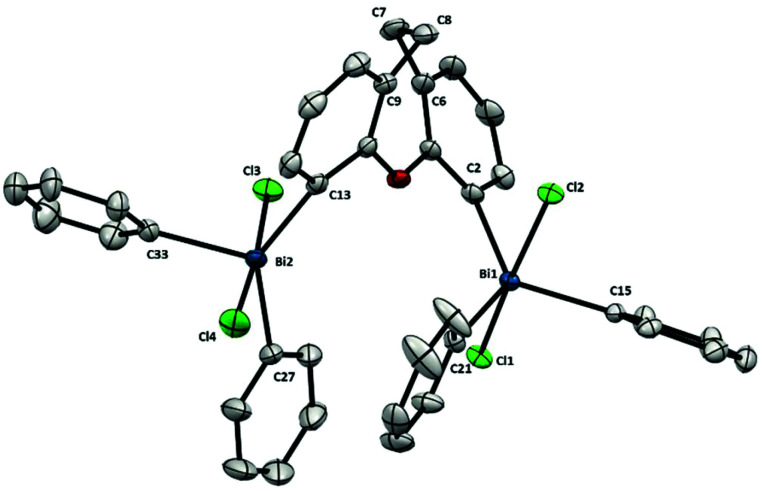
Structure of **11**. Ellipsoids are drawn at the 50% probability level and solvent and H atoms are omitted for clarity. Selected bond lengths (Å) and angles (°): Bi1–C2: 2.192(2), Bi1–C15: 2.200(2), Bi1–C21: 2.212(2), Bi1–Cl1: 2.5972(6), Bi1–Cl2: 2.5962(6), Bi2–C13: 2.218(3), Bi2–C27: 2.216(3), Bi2–C33: 2.206(3), Bi2–Cl3: 2.5881(7), Bi2–Cl4: 2.5856(7), C2–Bi1–C15: 119.38(9), C2–Bi1–C21: 127.71(9), C15–Bi1–C21: 112.80(9), Cl1–Bi1–Cl2: 170.725(19), C2–Bi1–Cl1: 88.91(7), C2–Bi1–Cl2: 85.48(7), C15–Bi1–Cl1: 88.26(6), C15–Bi1–Cl2: 88.06(6), C21–Bi1–Cl1: 96.08(7), C21–Bi1–Cl2: 93.19(7), C13–Bi2–C27: 138.46(10), C13–Bi2–C33: 110.94(10), C27–Bi2–C33: 110.60(11), Cl3–Bi2–Cl4: 175.59(2), C13–Bi2–Cl3: 91.39(7), C13–Bi2–Cl4: 89.29(7), C27–Bi2–Cl3: 90.69(9), C27–Bi2–Cl4: 91.67(9), C33–Bi2–Cl3: 87.62(7), C33–Bi2–Cl4: 88.07(7), C9–C8–C7: 109.6(2), C6–C7–C8: 110.1(2); Bi1⋯Bi2: 5.195(1) Å.

As represented in Fig. 8, the presence of bulky BiPh_2_Cl_2_-moieties in **11** has an influence on the backbone, as observed also in its analogue Bi(iii) **7** (Fig. 4). In agreement with the increase of steric hindrance of the Bi-centers (–BiPh_2_Cl_2_ in **11***vs.* –BiPh_2_**7**) the torsion in the ligand backbone is more accentuated, reflected in the angle between the planes of the benzo groups (in **11** is 46.39° and in **7** is 56.09°). This steric interaction between both bismuth centers is also shown in the distance between the Bi and the carbon at the backbone. For example, although attached to the same aryl unit, the Bi1–C2 distance is 2.192(2) Å whereas the distance of Bi2–C13 is elongated up to 2.218(3) Å. This Bi–C elongation is also observed in its 6-membered ring analogue **10** (Fig. 7) but not in the more rigid 5-member ring **9** (Fig. 6). The high repulsion and steric hindrance in **11** results in the increase of the Bi⋯Bi distance [5.195(1) Å *vs.* 3.807(1) Å from the trivalent analogue **7**]. Due to the higher degree of backbone flexibility in **11** compared to **10**, the Bi⋯Bi distance in **11** is slightly longer than in **10**, an opposite structural feature observed in the less sterically congested trivalent analogues (**7** and **6**).

**Fig. 9:**
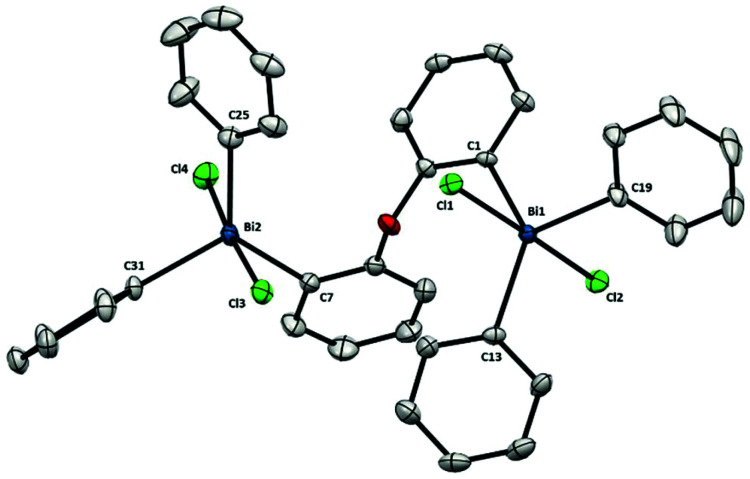
Structure of **12**. Ellipsoids are drawn at the 50% probability level and H atoms are omitted for clarity. Selected bond lengths (Å) and angles (°): Bi1–C1: 2.184(3), Bi1–C13: 2.199(3), Bi1–C19: 2.204(3), Bi1–Cl1: 2.5862(8), Bi1–Cl2: 2.5892(8), Bi2–C7: 2.189(3), Bi2–C25: 2.205(3), Bi2–C31: 2.214(3), Bi2–Cl3: 2.5677(8), Bi2–Cl4: 2.6191(8), C1–Bi1–C13: 130.36(11), C1–Bi1–C19: 109.56(12), C13–Bi1–C19: 120.09(12), Cl1–Bi1–Cl2: 175.42(3), C1–Bi1–Cl1: 86.12(8), C1–Bi1–Cl2: 90.01(8), C13–Bi1–Cl1: 92.04(8), C13–Bi1–Cl2: 88.59(8), C19–Bi1–Cl1: 92.47(9), C19–Bi1–Cl2: 91.13(9), C7–Bi2–C25: 128.79(12), C7–Bi2–C31: 114.23(12), C25–Bi2–C31: 116.77(12), Cl3–Bi2–Cl4: 176.06(3), C7–Bi2–Cl3: 93.06(9), C7–Bi2–Cl4: 83.75(9), C25–Bi2–Cl3: 89.99(9), C25–Bi2–Cl4: 90.22(9), C31–Bi2–Cl3: 91.45(8), C31–Bi2–Cl4: 91.96(9); Bi1⋯Bi2: 6.288(1) Å.

The structure of pentavalent dibismuthane **12** (Fig. 9) shows similarities to compound **9** and Ph_3_BiCl_2_.^23^ Therefore, each bismuth center adopts a slightly distorted trigonal bipyramidal geometry with the Cl in the apical positons. Similar to the parent Bi(iii) (**8**), the flexibility of the ligand backbone allows to diminish the steric constraints between both bismuth centers. Indeed, the two aryl groups from the backbone are placed almost perpendicular to each other [86.28(1)°]. Nevertheless, despite the free rotation of the ligand, the distance between both bismuth atoms is 6.288(1) Å, very similar to that found for **9**, which contains a rigid 5-member ring scaffold in the backbone.

### Catalytic oxidative cleavage of 1,2-diols

We have seen that dinuclear bismuthanes **5–8** show excellent air and moisture stability and high reactivity with strong oxidants such as SO_2_Cl_2_, affording the corresponding Bi(v) complexes **9–12** in almost quantitative yields. Barton and co-workers reported one of the first examples of bismuth redox catalysis, showing that catalytic amounts of triphenylbismuth together with NBS were able to catalyze the oxidative cleavage of 1,2-diols in excellent yields.^24^ The application of the rare examples of dibismuthanes reported has been relegated mainly to their use as ligands for transition metal complexes.^6^ To the best of our knowledge, there is no precedent on the catalytic redox behavior of such dimetallic compounds. Consequently, we decided to test our family of dibismuthanes **5–8** in the known oxidative cleavage of 1,2-diols.^25^ We began our investigations by screening dibismuthanes **5–8** towards the oxidative cleavage of 1,2-diphenylethane-1,2-diol **13** using the reaction conditions already developed by Barton and co-workers (Table 1). It was immediately revealing that the ligand backbone had an important effect on the catalytic performance. Whereas the catalytic activity of dibismuthanes **5–6** and **8** compared well with BiPh_3_ (Table 1, entry 1 *vs.* entries 2–3 and 5), dinuclear bismuthane **7** showed much lower activity. This was attributed to the close distance between both bismuth atoms (Fig. 4), increasing the steric hindrance and preventing catalytic activity.

**Table 1:** Catalyst screening for the oxidative cleavage of **13**^a^

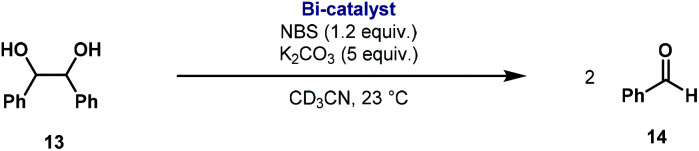			
Entry	Bi-catalyst	Time (min)	Yield^b^ (%)
1	**BiPh**_**3**_ (10 mol%)	10	>95
2	**5** (5 mol%)	10	>95
3	**6** (5 mol%)	10	>95
4	**7** (5 mol%)	10	55
5	**8** (5 mol%)	10	>95
6	**8** (2 mol%)	30	>95
7	—	30	<10

a Reaction conditions: **13** (0.12 mmol), **5–8** (5 or 2 mol%), NBS (1.2 equiv.), K_2_CO_3_ (5 equiv.) in 1.2 mL of CD_3_CN [0.1 M] at 23 °C for 10 or 30 min.

b Yields were determined by ^1^H NMR using mesitylene as internal standard.

Kinetic experiments showed that dibismuthane **8**, bearing the most flexible ligand, outperformed the remaining dinuclear bismuthanes, performing similarly to BiPh_3_ (see ESI† for details). Among dibismuthanes with rigid backbones (**5–7**) we found out that **5** performed slightly better than **6** (Bi⋯Bi distance of 5.544(1) and 4.187(1) Å, respectively) and it was superior to **7** (Bi⋯Bi distance of 3.807(1) Å). This trend is in perfect agreement with a lower catalytic activity when the dinuclear bismuthane bears a rigid backbone and a short Bi⋯Bi distance, two features that would increase the steric hindrance in the bismuth active centers. With the optimized reaction conditions in hand, we decided to study the ability of dibismuthane **8** towards the catalytic oxidative cleavage of different 1,2-diols (Table 2). Whereas 1,2-diphenylethane-1,2-diol **13** underwent oxidative cleavage quantitatively (Table 2, entry 1), more sterically hindered 1,1,2,2-tetraphenylethane-1,2-diol **15** required longer reaction time. Similarly, decane-1,2-diol **17** required longer time to be converted to nonanal **18** (Table 2, entry 3) compared to 1-phenylethane-1,2-diol **19**, which converted to the desired benzaldehyde **14** quantitatively in 30 min.

**Table 2:** Dibismuthane **8**-catalyzed oxidative cleavage of 1,2-diols^a^

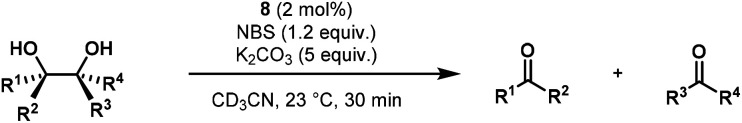			
Entry	1,2-Diol	Product	Yield^b^ (%)
1	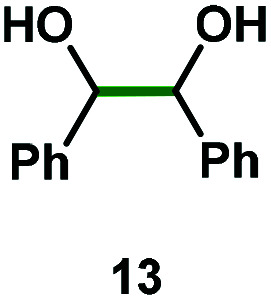	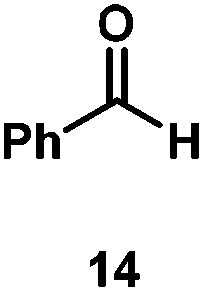	97 (88)
2^c^	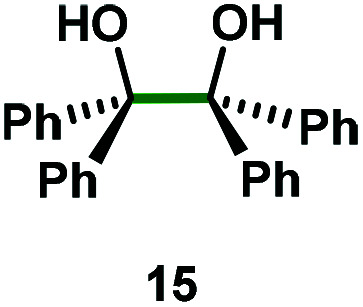	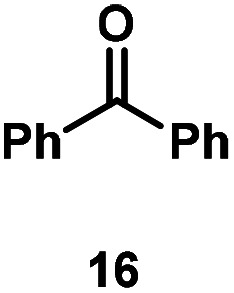	73 (68)
3^c^	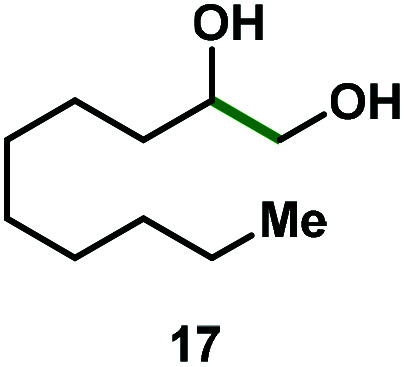	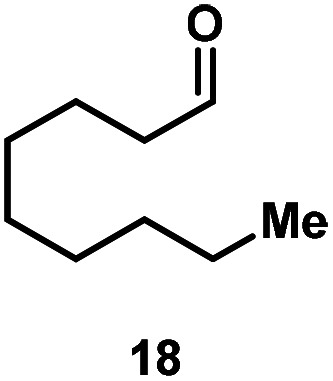	70 (66)
4	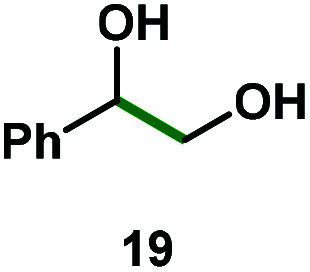	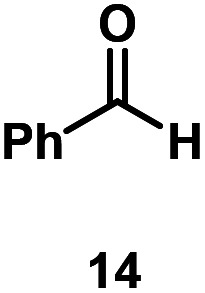	98 (91)
5	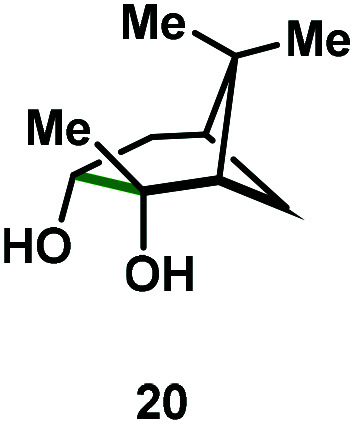	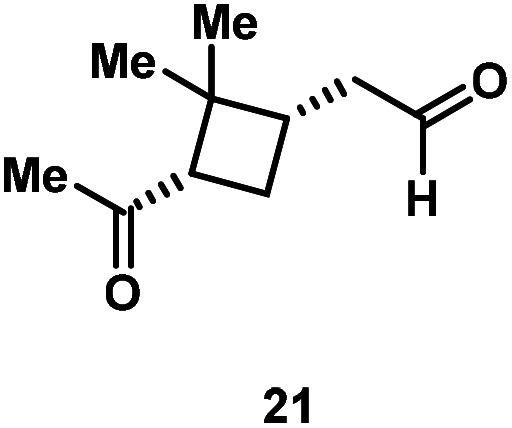	98 (94)

a Reaction conditions: 1,2-diols (0.12 mmol), **8** (2 mol%), NBS (1.2 equiv.), K_2_CO_3_ (5 equiv.) in 1.2 mL of CD_3_CN [0.1 M] at 23 °C for 30 min.

b Yields were determined by ^1^H NMR using mesitylene as internal standard. Isolated yields in parenthesis.

c Reaction time of 1 hour.

Application of the Bi-catalyzed protocol to the naturally occurring (1*S*,2*S*,3*R*,5*S*)-(+)-2,3-pinandiol (**20**) led to the quantitative conversion towards the dicarbonyl compound **21**. This last example is worth commenting further. Whereas most of the examples have freedom of rotation along the C–C bond, pinanediol has a rigid backbone, and therefore, the conformation of the diol is locked. However, this does not pose a problem in the C–C scission. This is in agreement with previous observations by Barton with a *cis*-decaline diol system.^24^ In cases where NBS might be problematic in certain synthetic endeavors, the reaction can also be carried out with stoichiometric amounts of the corresponding Ar_3_Bi(v)Cl_2_, obtaining virtually quantitative yields of the C–C cleavage (see ESI† for details). We believe that this reactivity could have interesting applications in the realm of natural product synthesis and further studies along these lines are currently under investigation in our laboratory.

## Conclusion

In summary, a variety of structurally different dinuclear bismuthanes have been synthesized and structurally characterized by single crystal X-ray diffraction. Fine-tuning of the ligand scaffold permitted a systematic evaluation of the influence of the backbone on the bismuth geometry and more importantly, on the Bi⋯Bi distance. Among these compounds, **7** revealed itself as a dibismuthane with an extremely short intramolecular Bi⋯Bi distance. Moreover, the oxidation of dibismuthanes **5–8** has been accomplished using SO_2_Cl_2_, isolating the corresponding pentavalent dibismuth tetrachlorides **9–12** in excellent yields. Studies on the catalytic redox properties of **5–8** revealed that the ligand backbone has a dramatic effect on the catalytic activity towards the oxidative cleavage of 1,2-diols. In this regard, dinuclear bismuthane **8** (with a flexible backbone and a long Bi⋯Bi distance) does not surpass the catalytic activity of triphenylbismuth. Further studies on the possible synergistic effects of two Bi atoms in the same complex towards catalytic redox processes are currently ongoing.

## Conflicts of interest

The authors declare no conflict of interest.

## Supplementary Material

OB-019-D1OB00367D-s001

OB-019-D1OB00367D-s002
