# Correction: Dibismuthanes in catalysis: from synthesis and characterization to redox behavior towards oxidative cleavage of 1,2-diols

**DOI:** 10.1039/d1ob90072b

**Published:** 2021-05-26

**Authors:** Marc Magre, Jennifer Kuziola, Nils Nöthling, Josep Cornella

**Affiliations:** Max-Planck-Institut für Kohlenforschung Mülheim an der Ruhr 45470 Germany cornella@kofo.mpg.de

## Abstract

Correction for ‘Dibismuthanes in catalysis: from synthesis and characterization to redox behavior towards oxidative cleavage of 1,2-diols’ by Marc Magre *et al.*, *Org. Biomol. Chem.*, 2021, DOI: 10.1039/d1ob00367d.

In the article, the authors claimed that oxidation of triaryldibismuthanes has not been explored. However, the authors overlooked an important precedent regarding such oxidation. The missing reference is listed below as ref. 1. The conclusions of this work remain unaltered. The authors apologize for any inconvenience.

The Royal Society of Chemistry apologises for these errors and any consequent inconvenience to authors and readers.
