# Supplementary material for: Dibismuthanes in catalysis: from synthesis and characterization to redox behavior towards oxidative cleavage of 1,2-diols
Source: Org Biomol Chem. 2021 May 6;19(22):4922–9. doi: 10.1039/d1ob00367d (PMC8188851; doi:10.1039/d1ob00367d)
Supplement: OB-019-D1OB00367D-s001 [file OB-019-D1OB00367D-s001.pdf]

Electronic Supplementary Material (ESI) for Organic & Biomolecular Chemistry.
This journal is © The Royal Society of Chemistry 2021

Supporting Information

Dibismuthanes in Catalysis: From Synthesis and Characterization to Redox Behavior towards Oxidative Cleavage of 1,2-Diols

Marc Magre, Jennifer Kuziola, Nils Nöthling and Josep Cornella*

Max-Planck-Institut für Kohlenforschung, D-45470 Mülheim/Ruhr, Germany

cornella@kofo.mpg.de

Table of Contents

1. General considerations S3

2. Synthesis of Ligands **3** and **4** S4

3. Synthesis of Dibismuthanes **5-8** S6

   3.1. Synthesis of Dibismuthane **5** S6

   3.2. Synthesis of Dibismuthane **6** S8

   3.3. Synthesis of Dibismuthane **7** S10

   3.4. Synthesis of Dibismuthane **8** S12

4. Synthesis of Pentavalent Dibismuth Compounds **9-12** S14

5. Low temperature and VT NMR analysis S19

   5.1. Compound **10** S19

   5.2. Compound **11** S21

6. Stoichiometric experiments of **9-12** for the oxidative cleavage of 1,2-diphenylethane-1,2-diol (**13)** S23

7. Kinetic experiments of **5-8** for Bi-catalyzed oxidative cleavage of 1,2-diphenylethane-1,2-diol (**13)** S24

8. Scope of Bi-catalyzed oxidative cleavage of 1,2-diols S26

9. References S29

10. NMR spectra S30

11. X-ray single crystal analysis S44

1. General considerations

Experimental methods

Unless otherwise stated, all manipulations were performed using standard Schlenk techniques under dry argon in flame-dried glassware. Anhydrous *n*-pentane, THF, Et_2O and toluene were distilled from appropriate drying agents and were transferred under argon.

Flash chromatography: Merck silica gel 60 (40-63 µm). Preparative TLC plates: PLC Silica gel 60 F_{254}, 1 mm, 20x20 cm (Sigma-Aldrich). ESI-MS: ESQ 3000 (Bruker). High-resolution mass determinations: Bruker APEX III FT-MS (7 T magnet) or MAT 95 (Finnigan). NMR spectra were recorded using 300 MHz Bruker Avance III, 400 MHz Bruker Avance III HD and 500 MHz Bruker Avance III NMR spectrometers. ^1H NMR spectra (300.13 MHz, 400.2 MHz, 500.1 Hz) were referenced to the residual protons of the deuterated solvent, and are reported to tetramethylsilane (δ TMS = 0 ppm), chloroform-*d* (δ_{TMS}= 7.26 ppm) or acetonitrile-d_3 (δ_{TMS}= 1.94ppm). $^{13}C\{^1H\}$ NMR spectra (75.47 MHz, 101 MHz, 125 MHz) were referenced internally to the D-coupled ^{13}C resonances of the NMR solvent and are reported to tetramethylsilane (δ_{TMS}= 0 ppm) and chloroform-*d* (δ_{TMS}= 77.16ppm). Chemical shifts (δ) are given in ppm, relative to deuterated solvent residual peak, and coupling constants (*J*) provided in Hz. C, H, Bi, Cl elemental analyses were performed by the Microanalytical Laboratory Kolbe.

2. Synthesis of Ligands 3 and 4

2.1 Synthesis of 4,6-dibromo-10,11-dihydrodibenzo[*b*,*f*]oxepine (3)

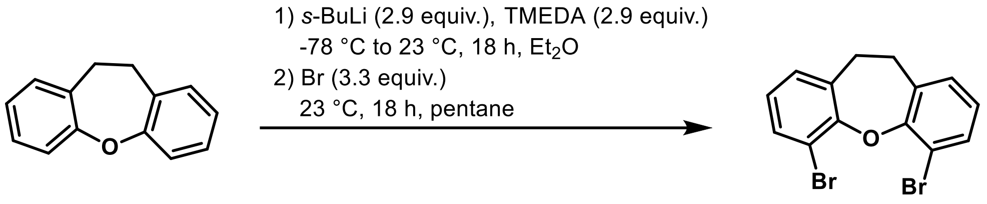

3.1 **3**

To a flame dried Schlenk-flask charged with a stir bar was added 10,11-dihydrodibenzo[*b*,*f*]oxepine[1,2] **(3.1)** (585 mg, 2.98 mmol, 1.0 equiv.), anhydrous Et_2O (28 mL), anhydrous TMEDA (1.3 mL, 8.6 mmol, 2.9 equiv.) and dropwise a solution of 1.4 M *s*-BuLi (6.2 mL, 8.64 mmol, 2.9 equiv.) at –78 °C. The mixture was warmed to 23 °C and left to stir for 18 h. The solution was cooled to –78 °C, followed by a slow addition of Br_2 (0.50 mL, 9.83 mmol, 3.3 equiv.) in pentane (6.5 mL) and allowed to stir for another 18 h at 23 °C. After completion, a saturated aqueous solution of $Na_2S_2O_3$ was added, followed by Et_2O and the layers were separated. The aqueous layer was washed with Et_2O (3 × 20 mL) and the combined organics were washed with $Na_2S_2O_3$, dried over $MgSO_4$, filtered and concentrated under reduced pressure. Purification via flash chromatography (SiO_2, 100% hexane) yielded 4,6-dibromo-10,11-dihydrodibenzo[*b*,*f*]oxepine **(3)** as a white solid (510 mg, 48% yield).

^{1}H NMR (300 MHz, $CDCl_3$): δ 7.47 (dd, *J* = 7.9, 1.7 Hz, 2H), 7.05 (dd, *J* = 7.6, 1.7 Hz, 2H), 6.89 (t, *J* = 7.7 Hz, 2H), 3.14 (s, 4H).

^{13}C NMR (75 MHz, $CDCl_3$): δ 152.7, 133.5, 132.0, 129.4, 124.7, 115.1, 32.1.

HRMS (ESI): calc'd for $C_{14}H_{10}O_1Br_2$ $[M]^+$ 351.909315; found 351.909350.

2.2 Synthesis of 2,2'-oxybis(iodobenzene) (4)

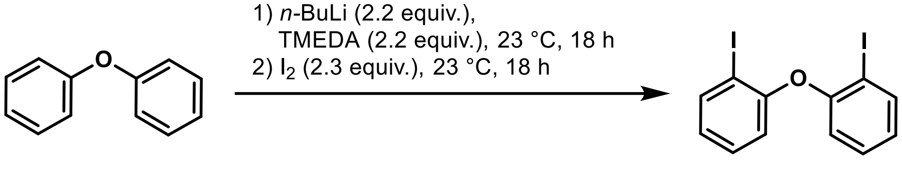

To a flame dried Schlenk-flask charged with a stir bar was added diphenylether **(4.1)** (1 g, 5.8 mmol, 1 equiv.), anhydrous THF (12 mL) and the solution was cooled to −78 °C. Then, anhydrous TMEDA (1.93 mL, 12.9 mmol, 2.2 equiv.) and a solution of 2.6 M *n*-BuLi (4.97 mL, 12.9 mmol, 2.9 equiv.) were added dropwise. The mixture was warmed to 23 °C and left to stir for 18 h. The solution was cooled to −78 °C, followed by a slow addition of I_2 (3.4 g, 13.5 mmol, 2.3 equiv.) and allowed to stir for another 18 h at 23 °C. After completion, a saturated aqueous solution of $Na_2S_2O_3$ was added, followed by Et_2O and the layers were separated. The aqueous layer was washed with Et_2O (3 × 10 mL) and the combined organics were washed with $Na_2S_2O_3$, dried over $MgSO_4$, filtered and concentrated under reduced pressure. Purification via flash chromatography (SiO_2, 100% hexane) yielded 2,2'-oxybis(iodobenzene) **(4)** as a white solid (1.047 g, 56% yield).

^{1}H NMR (300 MHz, $CDCl_3$): δ 7.88 (dd, *J* = 7.9, 1.6 Hz, 2H), 7.28 (ddd, *J* = 8.2, 7.3, 1.5 Hz, 2H), 6.89 (td, *J* = 7.6, 1.4 Hz, 2H), 6.78 (dd, *J* = 8.2, 1.4 Hz, 2H).

^{13}C NMR (75 MHz, $CDCl_3$): δ 156.0, 140.1, 129.6, 125.5, 118.7, 88.4.

HRMS (ESI): calc'd for $C_{12}H_8O_1I_2Na_1$ $[M+Na]^+$ 444.855679; found 444.856070.

3. Synthesis of Dibismuthanes 5-8

3.1 Synthesis of 4,6-bis(diphenylbismuthanyl)dibenzo[*b*,*d*]furan (5)

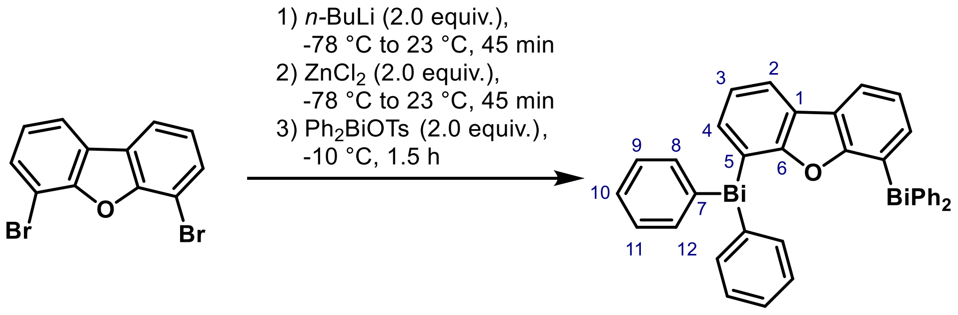

1 **5**

4,6-dibromodibenzofuran[3] **(1)** (150 mg, 0.46 mmol) was placed in a flame-dried Schlenk-flask under Ar atmosphere and dissolved in 6.5 mL of anhydrous THF. The solution was cooled to −78 °C and a solution of 2.6 M *n*-BuLi in hexane (0.35 mL, 0.92 mmol, 2.0 equiv.) was added dropwise. The mixture was warmed to 23 °C and left to stir for 45 min. Then, the mixture was cooled again to −78 °C and a solution of $ZnCl_2$ in anhydrous THF was added (0.92 mmol, 4.5 mL, 2.0 equiv.). The mixture was warmed to 23 °C and left to stir for 45 min. After this, Ph_2BiOTs[4] (491.8 mg, 0.92 mmol, 2.0 equiv.) was added in one portion at −10 °C, followed by the addition of additional 3 mL of anhydrous THF and the reaction was left to stir for 1.5 h at this temperature. The solution was quenched with a saturated aqueous solution of $NaHCO_3$ and diluted with Et_2O, whereupon it was extracted twice with Et_2O (2 × 8 mL). The combined organic phases were dried over $MgSO_4$, filtered and concentrated under reduced pressure (not to dryness!).[a] The crude reaction mixture was then purified by flash chromatography (SiO_2, 20:1 hexane:EtOAc). The obtained solid was washed further with cold pentane (2 × 5 mL) to yield the desired complex **5** as an off-white solid (173 mg, 42% yield).

^{1}H NMR (300 MHz, $CDCl_3$): δ 7.94 (dd, *J* = 7.6, 1.3 Hz, 2H [H_4]), 7.77 (dt, *J* = 5.9, 1.6 Hz, 8H [$H_{8,12}$]), 7.68 (dd, *J* = 7.2, 1.2 Hz, 2H, [H_2]), 7.32 (m, *J* = 8.0, 3.4 Hz, 14H [$H_{3,9,10,11}$]).

^{13}C NMR (75 MHz, $CDCl_3$): δ 170.7 [C_q], 160.0 [C_q], 137.9 [C_8], 136.0 [C_2], 130.5 [C_9], 127.8 [C_{10}], 125.5 [C_3], 123.6 [C_1], 120.7 [C_4].[b]

HRMS (ESI): calc'd for $C_{36}H_{27}O_1Bi_2$ $[M+H]^+$ 893.166410; found 893.166160.

EA: $C_{36}H_{27}O_1Bi_2\cdot H_2O$, calc'd C 47.49, H 3.10, Bi 45.90 %, exp. C 47.66, H 3.05, Bi 46.05 %.

X-ray quality crystals were obtained from slow evaporation of a solution of complex **5** in CH_2Cl_2:hexane (1:5) at 23 °C.

[a]Note: To avoid dismutation, which results in lower yields, it is advised to not to reach dryness.[4]

[b]Note: One quartenary carbon signal was not observed in the ^{13}C NMR spectra. To avoid a misassignment, the observable quaternary carbons were assigned as Cq.

3.2 Synthesis of (9,9-dimethyl-*9H*-xanthene-4,5-diyl)bis(diphenylbismuthane) (6)

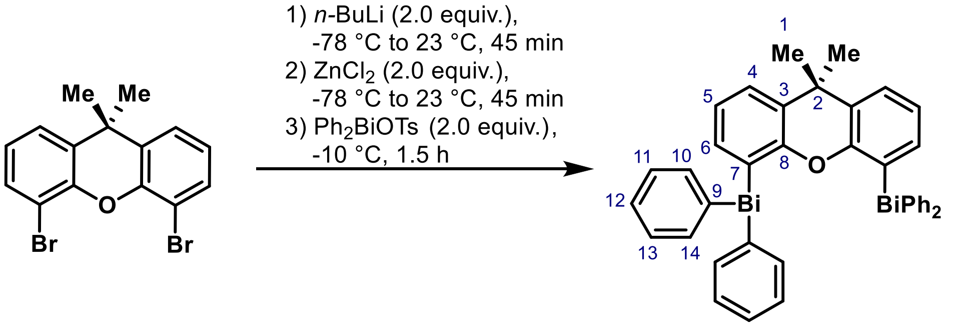

2 **6**

4,5-dibromo-9,9-dimethyl-9*H*-xanthene[5] **(2)** (555 mg, 1.5 mmol) was placed in a flame-dried Schlenk-flask under Ar atmosphere and dissolved in 25 mL of anhydrous THF. The solution was cooled to −78 °C and a solution of 2.6 M *n*-BuLi in hexane (0.28 mL, 3 mmol, 2.0 equiv.) was added dropwise. The mixture was warmed to 23 °C and left to stir for 45 min. After this time, the mixture was cooled again to −78 °C and a solution of $ZnCl_2$ in anhydrous THF was added (3.0 mmol, 15 mL, 2.0 equiv.). The mixture was warmed to 23 °C and left to stir for 45 min. Then, Ph_2BiOTs[4] (1.6 g, 3.0 mmol, 2.0 equiv.) was added in one portion at −10 °C, followed by the addition of additional 5 mL of anhydrous THF and the reaction was left to stir for 1.5 h at this temperature. The solution was quenched with a saturated aqueous solution of $NaHCO_3$ and diluted with Et_2O, whereupon it was extracted twice with Et_2O (2 × 25 mL). The combined organic phases were dried over $MgSO_4$, filtered and concentrated under reduced pressure (not to dryness!)[a]. The crude reaction mixture was then purified by flash chromatography (SiO_2, 20:1 hexane:EtOAc). The obtained solid was washed further with cold pentane (2 × 5 mL) to yield the desired complex **6** as an off-white solid (750 mg, 53% yield).

^{1}H NMR (300 MHz, $CDCl_3$): δ 7.66 (dd, *J* = 7.7, 1.6 Hz, 8H [$H_{10,14}$]), 7.47 (dd, *J* = 7.2, 1.5 Hz, 2H [H_6]), 7.42 (dd, *J* = 7.7, 1.5 Hz, 2H [H_4]), 7.37 – 7.26 (m, 12H [$H_{11,12,13}$]), 7.05 (dd, *J* = 7.7, 7.2 Hz, 2H [H_5]), 1.67 (s, 6H [H_1]).

^{13}C NMR (75 MHz, $CDCl_3$): δ 155.5 [C_q], 152.6 [C_q], 137.8 [C_{10}], 136.7 [C_6], 130.4 [C_{11}], 130.0 [C_3], 127.6 [C_{12}], 126.6 [C_5], 126.3 [C_4], 35.2 [C_2], 32.5 [C_1].[b]

HRMS (ESI): calc. for $C_{39}H_{33}Bi_2O_1$ $[M+H]^+$ 935.2133; found 935.2131.

EA: $C_{39}H_{32}Bi_2O{\cdot}0.5H_2O$, calc'd C 49.64, H 3.53, Bi 44.29 %; exp. C 49.76, H 3.51, Bi 44.44 %.

X-ray quality crystals were obtained from a phase transfer diffusion (5:1) of pentane into a concentrated solution of complex **6** in CH_2Cl_2 at +5 °C.

[a]Note: To avoid dismutation, which results in lower yields, it is advised to not to reach dryness.[4]

[b]Note: One quartenary carbon signal was not observed in the ^{13}C NMR spectra. To avoid a misassignment, the observable quaternary carbons were assigned as Cq.

3.3 Synthesis of 4,6-bis(diphenylbismuthanyl)-10,11-dihydrodibenzo[*b*,*f*]oxepine (7)

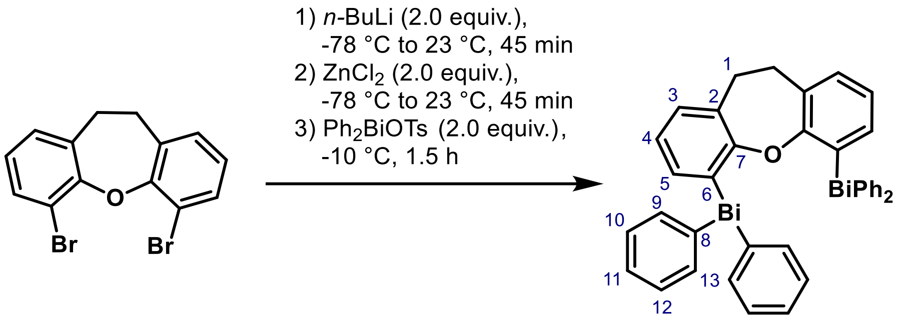

3 7

4,6-dibromo-10,11-dihydrodibenzo-oxepine **(3)** (200 mg, 0.56 mmol) was placed in a flame-dried Schlenk-flask under Ar atmosphere and dissolved in 9 mL of anhydrous THF. The solution was cooled to –78 °C and a solution of 2.6 M *n*-BuLi in hexane (0.43 mL, 1.1 mmol, 2.0 equiv.) was added dropwise. The mixture was stirred at 23 °C for 45 min. After this time, the mixture was cooled again to –78 °C and a solution of $ZnCl_2$ in anhydrous THF was added (1.1 mmol, 6 mL, 2 equiv.). The mixture was warmed to 23 °C and left to stir for 45 min. Then, Ph_2BiOTs[4] (603.7 mg, 1.1 mmol, 2.0 equiv.) was added in one portion at –10 °C, followed by the addition of additional 4 mL of anhydrous THF and the reaction was left to stir for 1.5 h at this temperature. The reaction mixture was quenched with a saturated aqueous solution of $NaHCO_3$ and diluted with Et_2O, whereupon it was extracted twice with Et_2O (2 × 8 mL). The combined organic phases were dried over $MgSO_4$, filtered and concentrated under reduced pressure (not to dryness!)[a]. The crude reaction mixture was then purified by flash chromatography (SiO_2, 20:1 hexane:EtOAc). The obtained solid was washed further with cold pentane (2 × 5 mL) to yield the desired complex **7** as an off-white solid (234 mg, 45% yield).

^{1}H NMR (300 MHz, $CDCl_3$): δ 7.64 – 7.59 (m, 8H [$H_{9,13}$]), 7.53 (dd, *J* = 7.2, 1.7 Hz, 2H [H_5]), 7.36 – 7.26 (m, 12H [$H_{10,11,12}$]), 7.08 (dd, *J* = 7.4, 1.7 Hz, 2H [H_3]), 6.92 (t, *J* = 7.3 Hz, 2H [H_4]), 3.13 (s, 4H [H_1]).

^{13}C NMR (75 MHz, $CDCl_3$): δ 158.9 [C_q], 157.3 [C_q], 138.2 [C_9], 137.7 [C_5], 132.0 [C_2], 131.4 [C_3], 130.8 [C_{10}], 128.0 [C_{11}], 126.9 [C_4], 34.5 [C_1].[b]

HRMS (ESI): calc'd for $C_{38}H_{30}Bi_2O_1Na_1$ $[M+Na]^+$ 943.17965; found 943.179980.

EA: $C_{38}H_{30}Bi_2O{\cdot}0.5H_2O$, calc'd C 49.10, H 3.36, Bi 44.96 %; exp. C 49.30, H 3.27, Bi 45.12 %.

X-ray quality crystals were obtained from slow evaporation of a solution of complex **7** in CH_2Cl_2:hexane (1:5) at 23°C.

[a]Note: To avoid dismutation, which results in lower yields, it is advised to not to reach dryness.[4]

[b]Note: One quartenary carbon signal was not observed in the ^{13}C NMR spectra. To avoid a misassignment, the observable quaternary carbons were assigned as Cq.

3.4 Synthesis of (oxybis(2,1-phenylene))bis(diphenylbismuthane) (8)

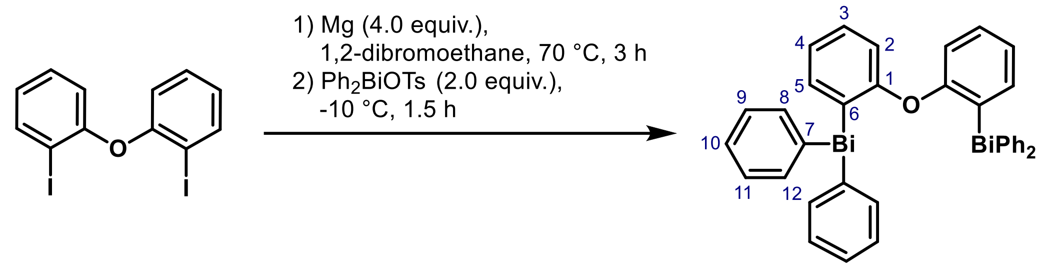

4 **8**

A flame-dried Schlenk-flask was charged with activated magnesium turnings (41.4 mg, 1.7 mmol, 4.0 equiv.) and anhydrous THF (0.5 mL) under Ar atmosphere, followed by addition of 1,2-dibromoethane (38.6 µL, 1.05 equiv.) and 10 mg (0.0237 mmol) of 2,2'-oxybis(iodobenzene) **(4)**. This mixture was gently heated with a heat gun (70 °C) and a solution of the remaining 2,2'-oxybis(iodobenzene) **(4)** (170 mg, 0.4028 mmol) in anhydrous THF (6.2 mL) was slowly added. The mixture was placed in an oil bath and heated at 70 °C for 3 h. Then, the solution was cooled to room temperature, additional 15 mL of anhydrous THF were added and the mixture was cooled to –10 °C. Finally, Ph_2BiOTs[4] (455.8 mg, 0.85 mmol, 2 equiv.) was added in one portion and the solution was left to stir for 1.5 h at –10 °C. The mixture was quenched with a saturated aqueous solution of $NaHCO_3$ and diluted with Et_2O, whereupon it was extracted twice with Et_2O (2 × 10 mL). The combined organic phases were dried over $MgSO_4$, filtered and concentrated under reduced pressure (not to dryness!).[a] The crude reaction mixture was then purified by flash chromatography (SiO_2, 20:1 hexane:EtOAc). The obtained solid was washed further with cold pentane (2 × 5 mL) to yield the desired complex **8** as an off-white solid (104 mg, 33% yield).

^{1}H NMR (400 MHz, $CDCl_3$): δ 7.68 – 7.61 (m, 10H, [$H_{5,8,12}$]), 7.38 – 7.28 (m, 12H, [$H_{9,10,11}$]), 7.23 (ddd, *J* = 8.1, 7.2, 1.7 Hz, 2H [H_3]), 7.04 (td, *J* = 7.3, 1.1 Hz, 2H (H_4)), 6.93 (dd, *J* = 8.1, 1.1 Hz, 2H (H_2)).

^{13}C NMR (101 MHz, $CDCl_3$): δ 159.6 [C_q], 155.4 [C_1], 138.9 [C_5], 137.9 [C_8], 130.4 [C_9], 129.6 [C_3], 127.6 [C_{10}], 126.5 [C_4], 117.4 [C_2].[b]

HRMS (ESI): calc'd for $C_{36}H_{28}O_1Bi_2Na_1$ $[M+Na]^+$ 917.16400; found 917.164080.

EA: $C_{36}H_{28}Bi_2O$, calc'd C 48.34, H 3.16, Bi 46.72 %; exp. C 48.24, H 3.35, Bi 46.61 %.

X-ray quality crystals were obtained a phase transfer diffusion (5:1) of pentane into a concentrated solution of complex **8** in Et_2O at 23 °C.

[a]Note: To avoid dismutation, which results in lower yields, it is advised to not to reach dryness.[4]

[b]Note: One quartenary carbon signal was not observed in the ^{13}C NMR spectra. To avoid a misassignment, the observable quaternary carbons were assigned as Cq.

4. Synthesis of Pentavalent Dibismuth Compounds 9-12

General Synthesis

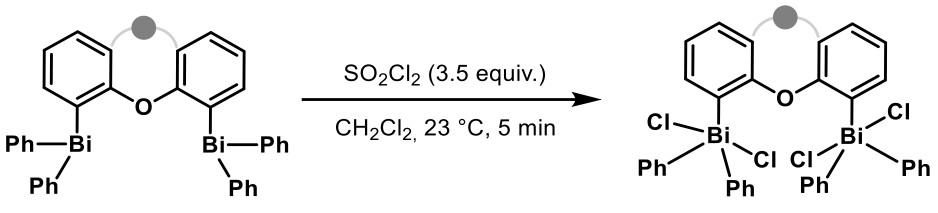

In a flame-dried Schlenk-flask under Ar atmosphere, the corresponding dibismuthane (1.0 equiv.) was dissolved in anhydrous CH_2Cl_2 (6 mL) and SO_2Cl_2 (3.5 equiv.) was added. After 5 min, the solvent was evaporated. The crude was washed with Et_2O (2 × 10 mL), affording the corresponding pentavalent dibismuth **9-12** as yellow solids.

4,6-bis(dichlorodiphenyl- λ^5-bismuthanyl)dibenzo[*b*,*d*]furan (9)

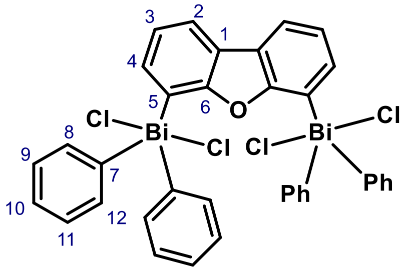

9

Yield: 201 mg (96%).

^{1}H NMR (400 MHz, $CDCl_3$): δ 8.50 – 8.45 (m, 8H [$H_{8,12}$]), 8.15 (dd, *J* = 7.6, 1.1 Hz, 2H [H_4]), 8.07 (dd, *J* = 7.9, 1.1 Hz, 2H [H_2]), 7.66 – 7.60 (m, 8H [$H_{9,11}$]), 7.56 (t, *J* = 7.7 Hz, 2H [H_3]), 7.53 – 7.47 (m, 4H [H_{10}]).

^{13}C NMR (101 MHz, $CDCl_3$): δ 154.9 [C_q], 154.6 [C_q], 141.9 [C_q], 134.3 [C_8], 132.4 [C_2], 131.9 [C_9], 131.6 [C_{10}], 126.9 [C_1], 125.8 [C_3], 124.0 [C_4].

HRMS (ESI): calc'd for $C_{36}H_{26}Bi_2Cl_4O_1Na_1$ $[M+Na]^+$ 1055.02376; found 1055.02316.

EA: $C_{36}H_{26}Bi_2Cl_4O_1 \cdot H_2O$, calc'd C 41.09, H 2.68, Bi 39.72, Cl 13.47%; exp. C 40.89, H 2.47, Bi 39.39, Cl 13.34 %.

X-ray quality crystals were obtained by vapour diffusion of a solution of complex **9** in CH_2Cl_2:pentane (1:5).

[b]Note: To avoid a misassignment, the observable quaternary carbons were assigned as Cq.

(9,9-dimethyl-9*H*-xanthene-4,5-diyl)bis(dichlorodiphenyl-λ^5-bismuthane) (10)

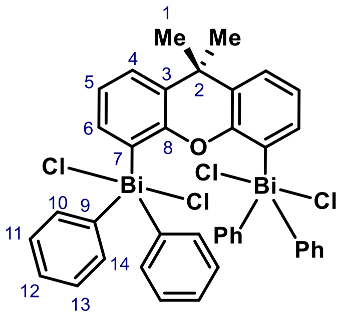

Yield: 194 mg (94%).

^{1}H NMR (300 MHz, $CDCl_3$): δ 8.15 (d, *J* = 7.7 Hz, 8H [$H_{10,14}$]), 7.94 (d, *J* = 8.0 Hz, 2H [H_6]), 7.59 (d, *J* = 7.6 Hz, 2H [H_4]), 7.40 (dd, *J* = 11.7, 7.1 Hz, 12H [$H_{11,12,13}$]), 7.27 (t, *J* = 7.3 Hz, 2H [H_5]), 1.75 (s, 6H [H_1]).

^{13}C NMR (75 MHz, $CDCl_3$): δ 159.3 [C_q], 150.1 [C_q], 134.8 [C_3], 134.0 [C_{10}], 132.5 [C_6], 131.4 [C_{11}], 130.7 [C_{12}], 128.1 [C_4], 126.2 [C_5], 36.9[C_2], 30.8[C_1].[b]

HRMS (ESI): calc'd for $C_{39}H_{32}Bi_2Cl_3O_1$ [M-Cl]$^+$ 1039.1121; found 1039.1117.

EA: $C_{39}H_{32}Bi_2Cl_4O_1$, calc'd C 43.52, H 3.00, Bi 38.83, Cl 13.17 %; exp. C 43.35, H 3.05, Bi 38.54, Cl 13.02 %.

X-ray quality crystals were obtained from slow evaporation of a solution of complex **10** in CH_2Cl_2:hexane (1:5).

[b]Note: One quartenary carbon signal was not observed in the ^{13}C NMR spectra. To avoid a misassignment, the observable quaternary carbons were assigned as Cq.

4,6-bis(dichlorodiphenyl- λ^5-bismuthanyl)-10,11-dihydrodibenzo[*b,f*]oxepine (11)

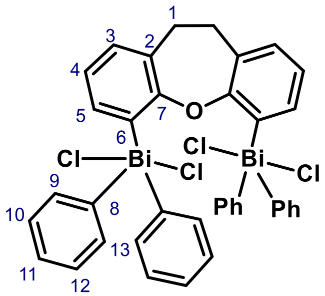

11

Yield: 210 mg (97%).

^{1}H NMR (400 MHz, $CDCl_3$): δ 8.23 – 8.18 (m, 8H, [$H_{9,13}$]), 7.98 (dd, *J* = 7.8, 1.6 Hz, 2H [H_5]), 7.57 – 7.51 (m, 8H [$H_{10,12}$]), 7.48 – 7.43 (m, 4H [H_{11}]), 7.28 – 7.25 (m, 2H [H_3]), 7.22 – 7.17 (m, 2H [H_4]), 3.48 – 3.05 (m, 4H [H_1]).

^{13}C NMR (101 MHz, $CDCl_3$): δ 155.4 [C_q], 153.1 [C_q], 152.8 [C_q], 136.0 [C_2], 134.1 [C_9], 133.7 [C_3], 132.0 [C_{10}], 131.9 [C_5], 131.2 [C_{11}], 124.6 [C_4], 36.8 [C_1].[b]

HRMS (ESI): calc'd for $C_{38}H_{30}Bi_2Cl_4O_1Na_1$ $[M+Na]^+$ 1083.05506; found 1083.056120.

EA: $C_{38}H_{30}Bi_2Cl_4O_1$, calc'd C 42.94, H 2.83, Bi 39.31, Cl 13.36 %; exp. C 42.96, H 2.85, Bi 39.34, Cl 13.35%.

X-ray quality crystals were obtained from liquid transfer diffusion of a mixture of C_6D_6:pentane (1:1) at 23 °C.

[b]Note: To avoid a misassignment, the observable quaternary carbons were assigned as Cq.

Oxybis(2,1-phenylene))bis(dichlorodiphenyl- λ^5-bismuthane) (12)

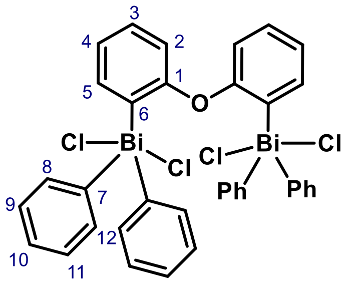

12

Yield: 195 mg (93%).

^{1}H NMR (400 MHz, $CDCl_3$): δ 8.51 – 8.45 (m, 8H [$H_{8,12}$]), 7.85 (dd, *J* = 7.9, 1.5 Hz, 2H [H_5]), 7.64 – 7.58 (m, 10H [$H_{3,9,11}$], 7.49 – 7.44 (m, 4H [H_{10}]), 7.44 – 7.40 (m, 2H [H_2]), 7.37 – 7.32 (m, 2H [H_4]).

^{13}C NMR (101 MHz, $CDCl_3$): δ 155.9 [C_q], 154.1 [C_q], 153.6 [C_q], 134.4 [C_8], 132.84 [C_2], 132.2 [C_9+C_5], 131.5 [C_{10}], 127.4 [C_4], 123.2 [C_3].[b]

HRMS (ESI): calc'd for $C_{36}H_{28}Cl_3Bi_2O_1$ $[M-Cl]^+$ 999.08079; found 999.07967.

EA: $C_{36}H_{28}Bi_2Cl_4O_1 \cdot H_2O$, calc'd C 41.01, H 2.87, Bi 39.64, Cl 13.45 %; exp. C 40.90, H 3.03, Bi 39.53, Cl 13.41 %

X-ray quality crystals were obtained from slow evaporation of a solution of complex **12** in CH_2Cl_2:hexane (1:5).

[b]Note: To avoid a misassignment, the observable quaternary carbons were assigned as Cq.

5. Low temperature and VT NMR analysis

5.1. Pentavalent Bi–(V) 10

^{1}H NMR of **10** (400 MHz in CD_2Cl_2) at 23 °C

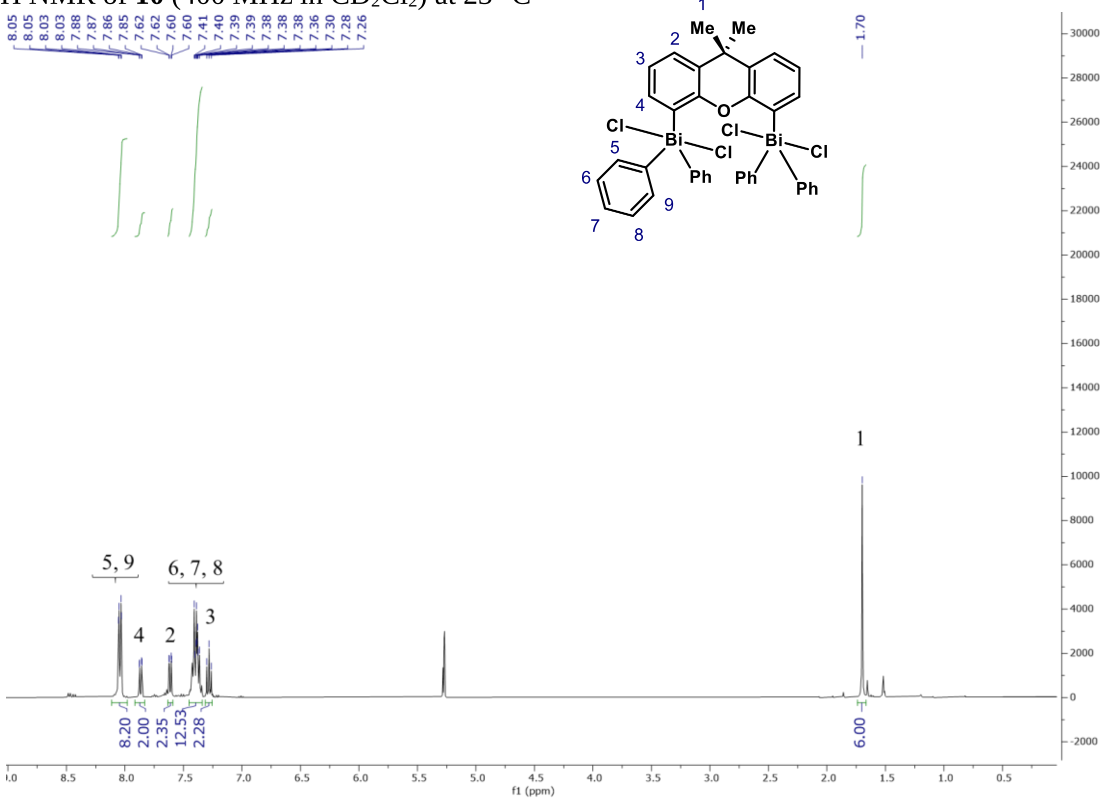

^{1}H NMR of **10** (400 MHz in CD_2Cl_2) at -90 °C

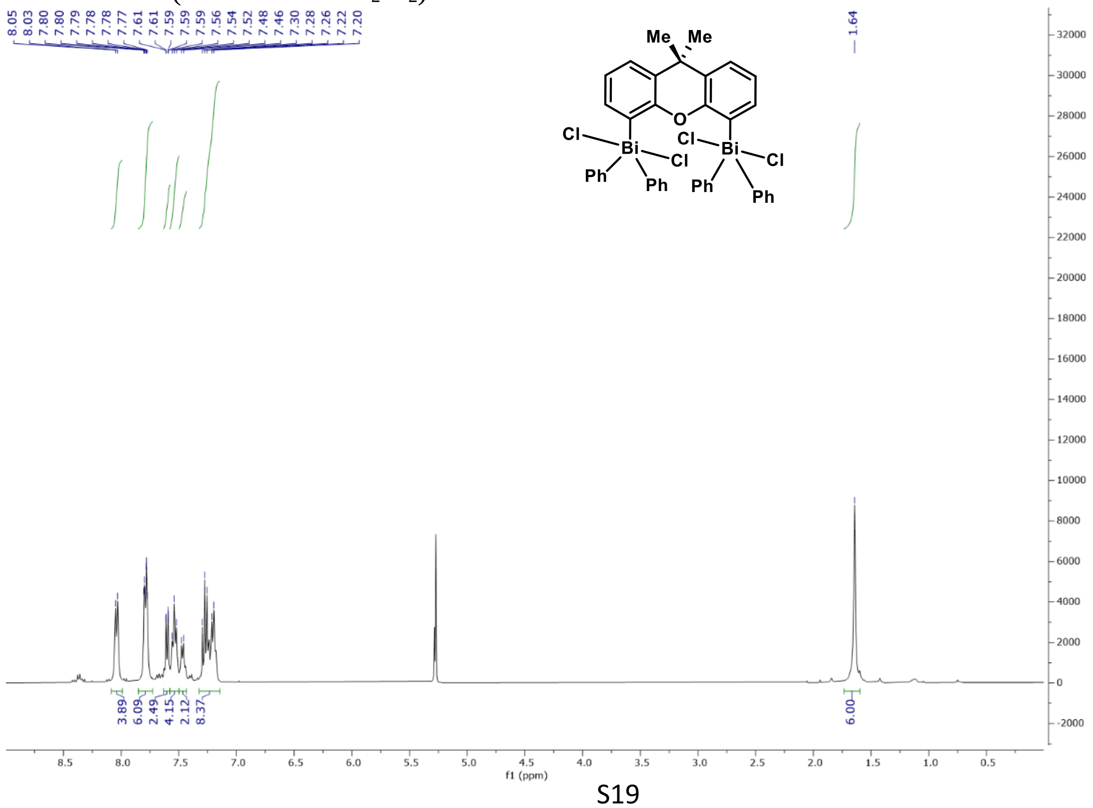

VT ^{1}H NMR of **10** (500 MHz in CD_2Cl_2) from 23 °C (bottom) to -90 °C (top)

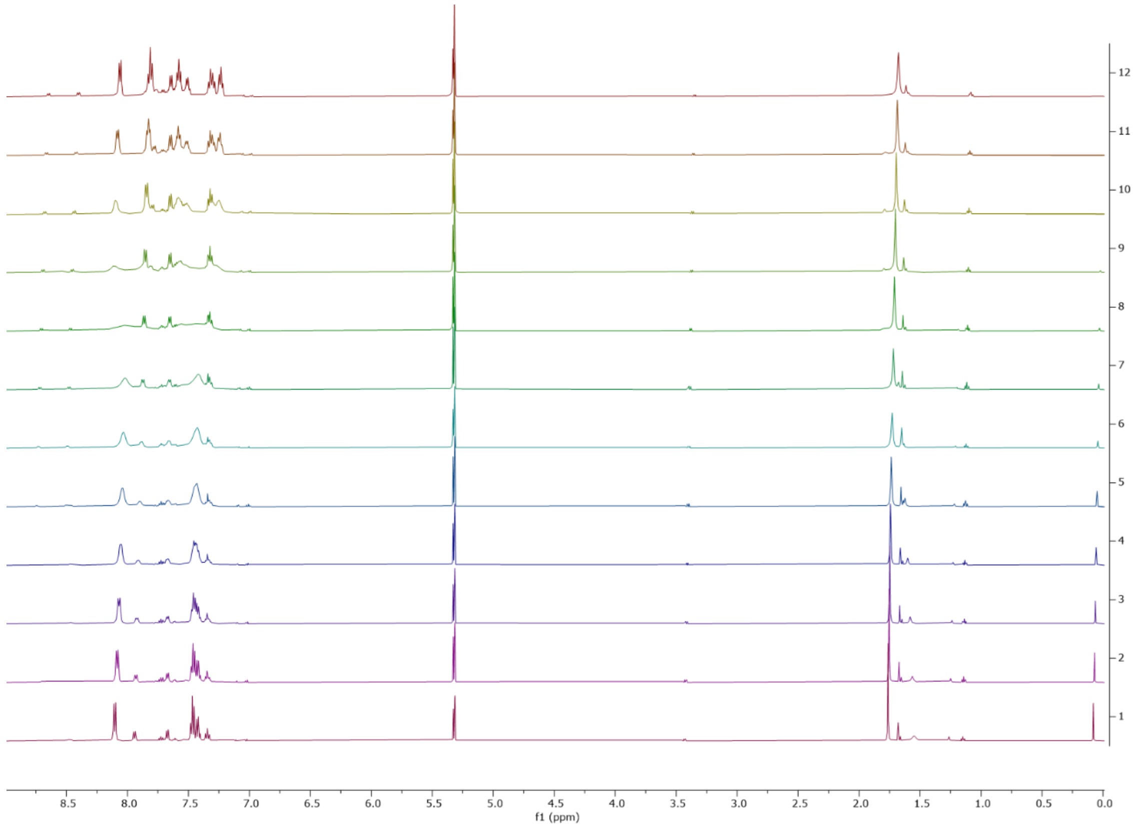

Zoom area: 10.00 – 6.50 ppm

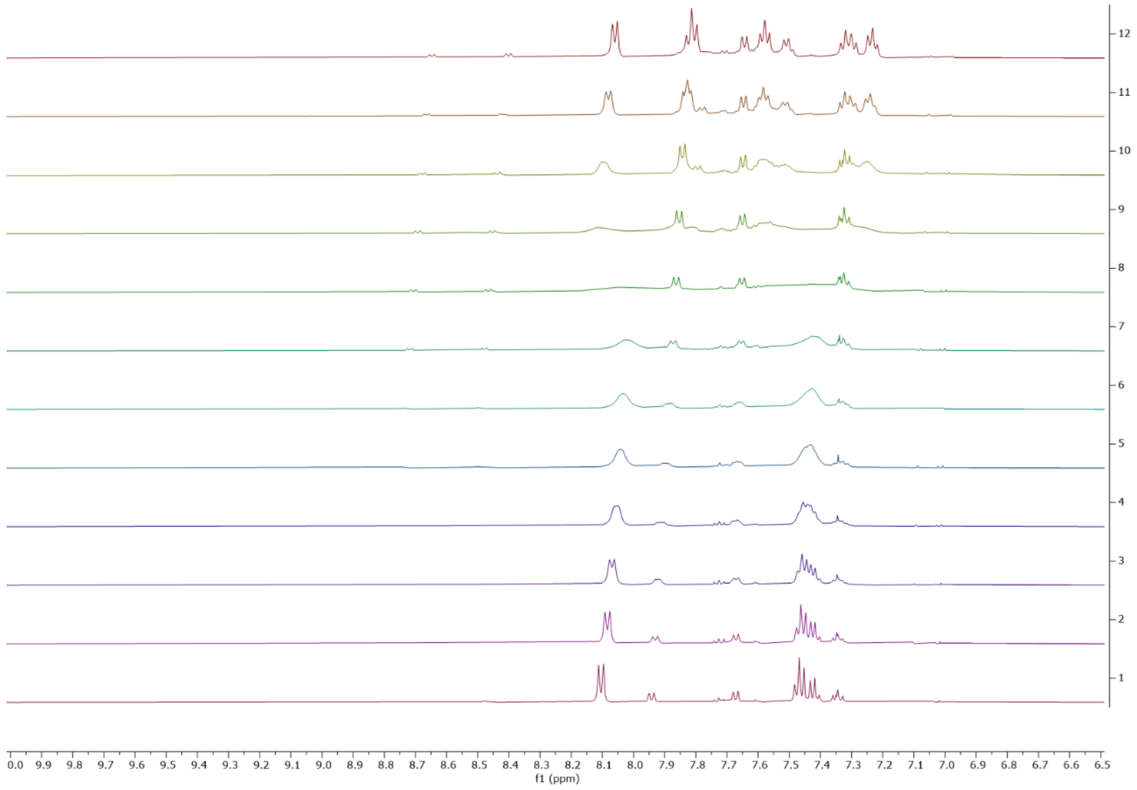

5.2. Pentavalent Bi–(V) 11

^{1}H NMR of **11** (500 MHz in CD_2Cl_2) at 23 °C

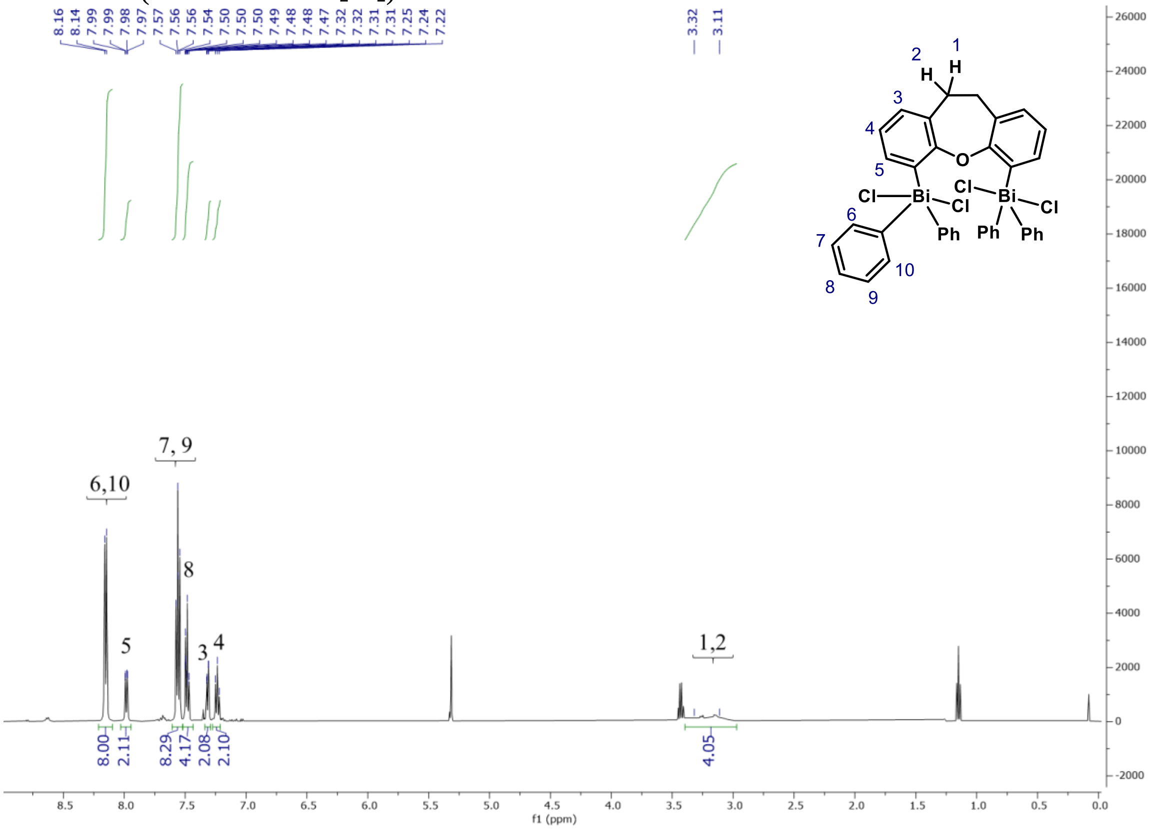

^{1}H NMR of **11** (500 MHz in CD_2Cl_2) at -90 °C

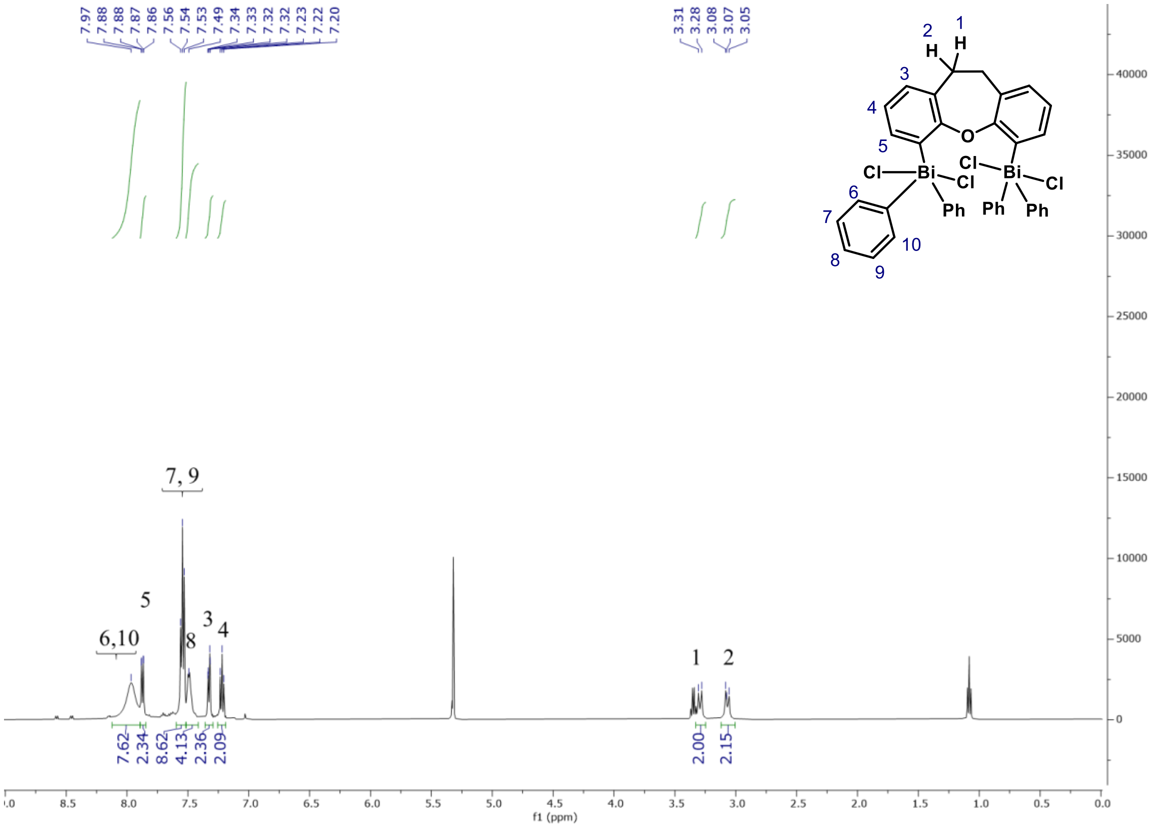

VT ^{1}H NMR of **11** (500 MHz in CD_2Cl_2) from 23 °C (bottom) to -90 °C (top)

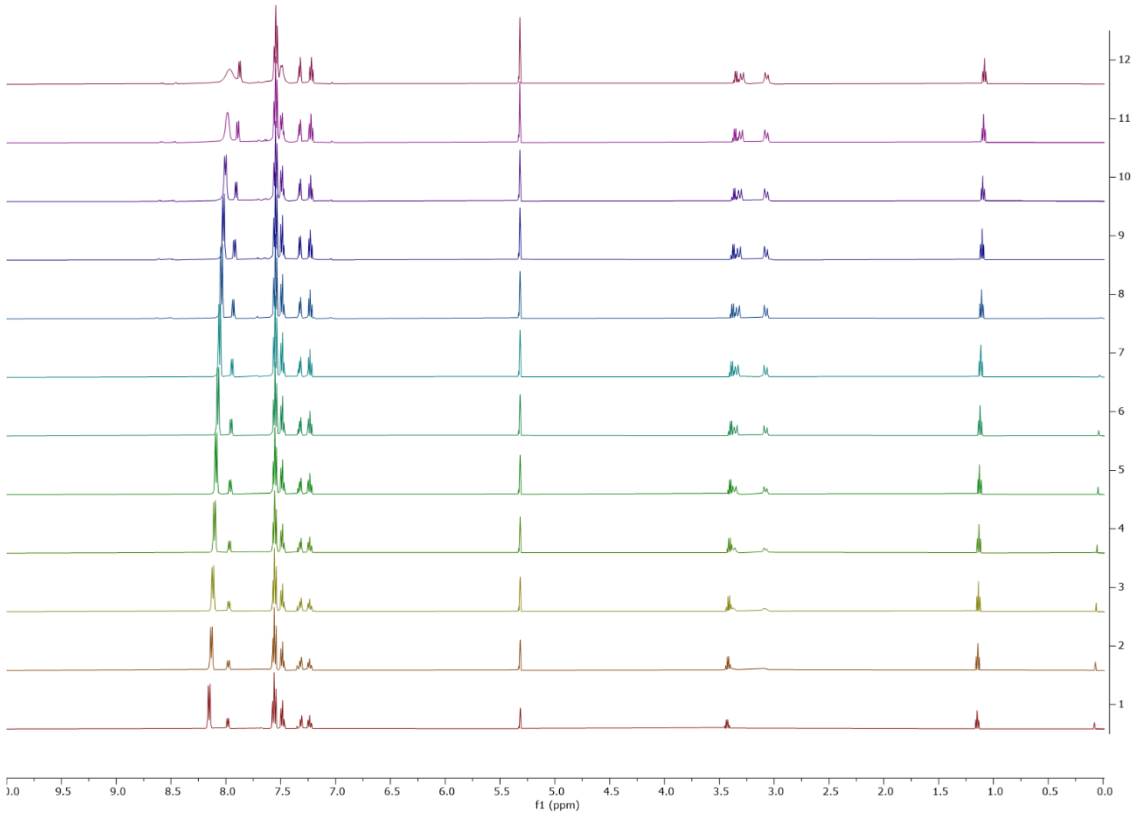

Zoom area: 10.00 – 6.50 ppm

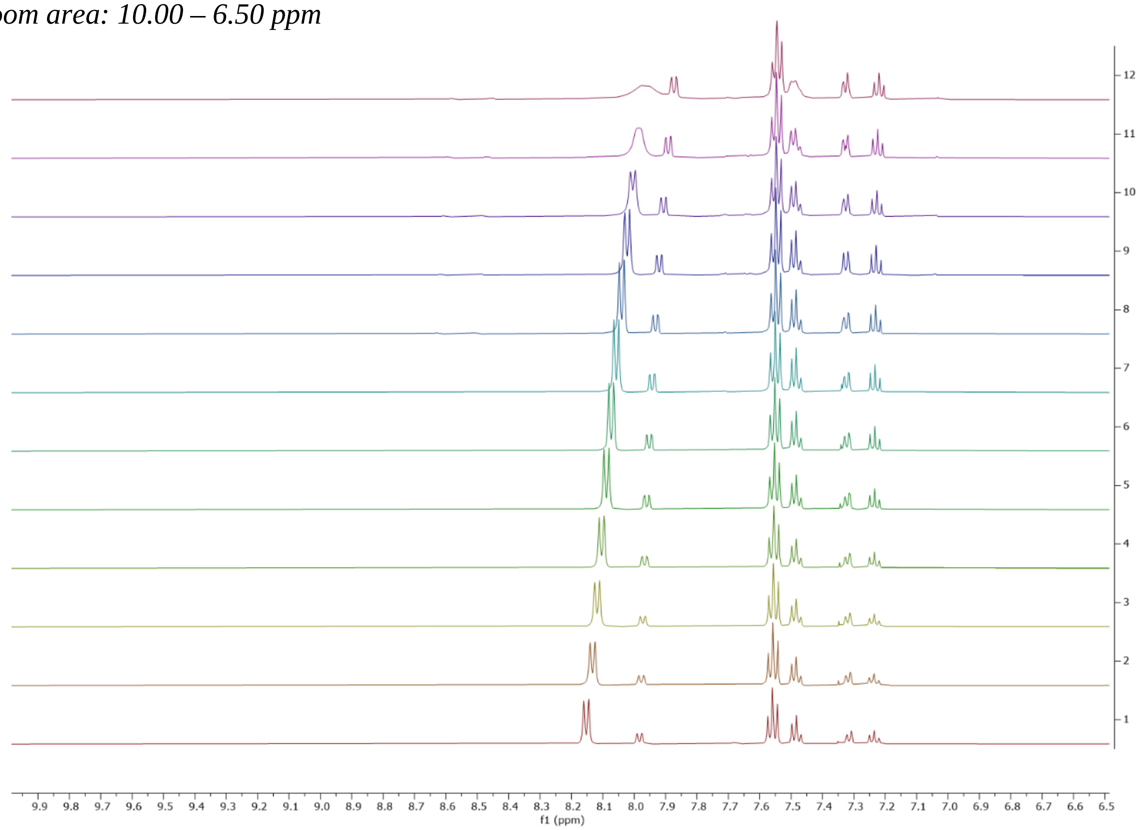

6. Stoichiometric experiments of 9-12 for the oxidative cleavage of 1,2-diphenylethane-1,2-diol (13)

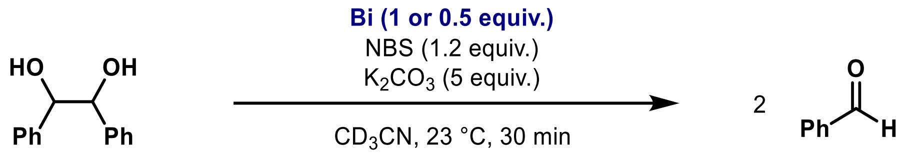

13 14

Entry[a]	Bismuth (V) reagent	Yield (%)[b]
1	Ph_3BiCl_2 (1 equiv.)	90
2	**9** (0.5 equiv.)	90
3	**10** (0.5 equiv.)	89
4	**11** (0.5 equiv.)	93
5	**12** (0.5 equiv.)	92

[a] Reaction conditions: **13** (0.12 mmol), **Bi–(V) reagent** (1 or 0.5 equiv.) NBS (1.2 equiv.), K_2CO_3 (5 equiv.) in 1.2 mL of CD_3CN [0.1 M] at 23 °C for 30 min. [b] Yields were determined by ^{1}H NMR using mesitylene as internal standard.

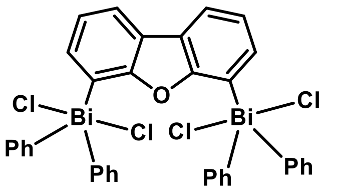

9

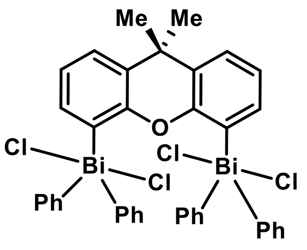

10

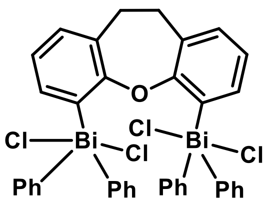

11

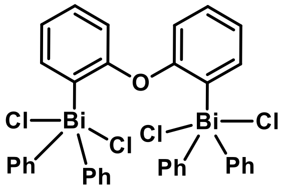

12

7. Kinetic experiments of 5-8 and $BiPh_3$ for Bi-catalyzed oxidative cleavage of 1,2-diphenylethane-1,2-diol (13)

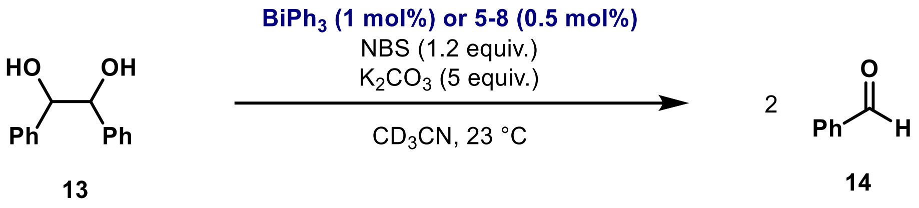

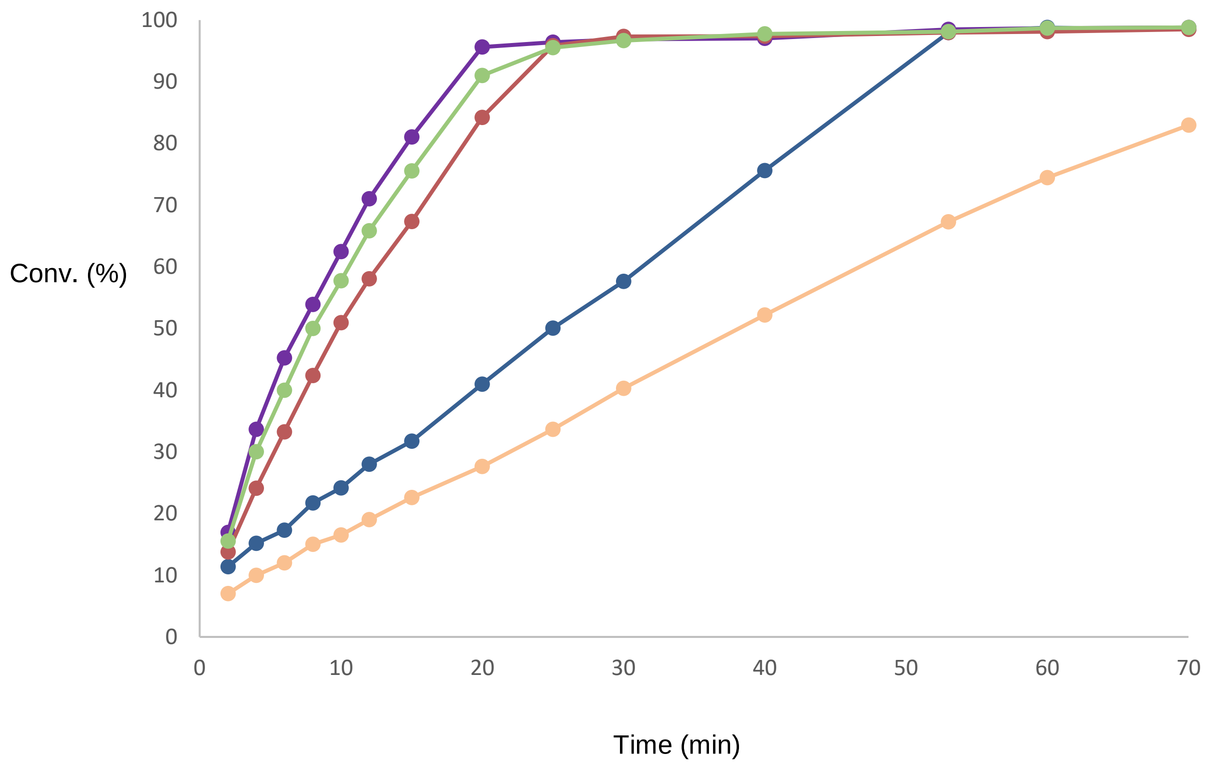

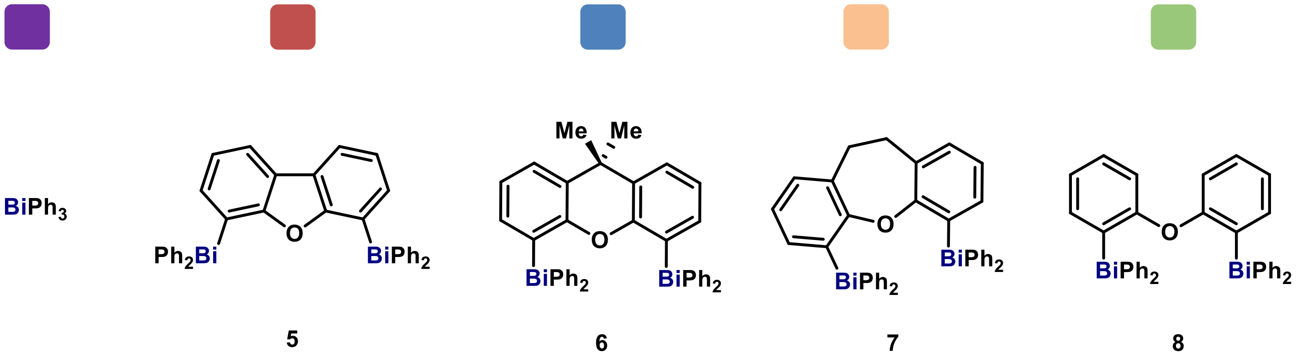

Barton´s proposed mechanism

Barton and co-workers proposed a mechanism for the Bi-catalyzed oxidative cleavage of 1,2-diols based on NMR spectroscopy and experimental evidences.[6] In the first step, the glycol reacts with NBS to form a hypobromite species, which acts as an oxidant of $BiPh_3$ to form a pentavalent Bi-alcoxy intermediate. The last step is a base-induced reductive elimination with cleavage of the C-C bond to the carbonyl derivatives and regenerating triphenylbismuth.

Barton's proposed mechanism (NBS-BiPh$_3$-K$_2$CO$_3$ system)

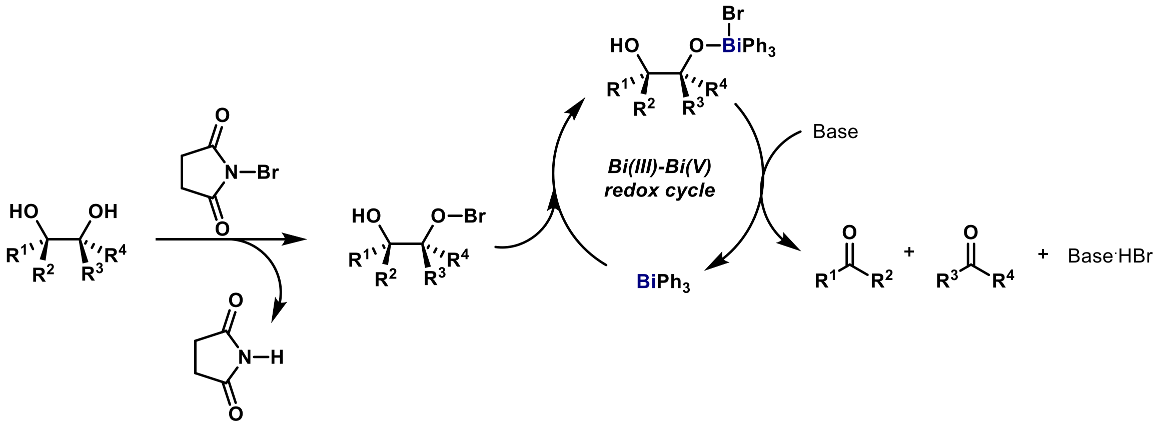

8. Scope of Bi-catalyzed oxidative cleavage of 1,2-diols

General procedure for Bi-catalyzed the oxidative cleavage of 1,2-diols

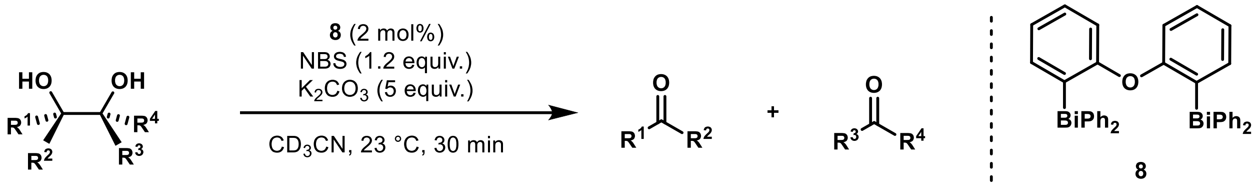

In a culture tube the corresponding 1,2-diol (0.12 mmol), K_2CO_3 (83 mg, 5.0 equiv.), dibismuthane **8** (2.1 mg, 2 mol%) and mesitylene (16.7 μL, 1.0 equiv.) were dissolved in 0.6 mL CD_3CN and stirred for 2 min. After that, a solution of NBS (25.6 mg, 1.2 equiv.) in 0.6 mL of CD_3CN was added dropwise and the reaction was left at 23 °C for the desired time (see Table 2 in the manuscript). An aliquot was taken and ^{1}H NMR was recorded to determine the NMR yield. The sample was returned to the reaction crude and solvent was evaporated. The reaction crude was purified via flash chromatography (SiO_2, 8:2 pentane:Et_2O) to afford the corresponding carbonyl compounds.

Benzaldehyde (14) (Table 2, entry 1)

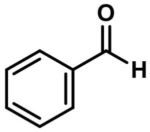

14

Yield: 22.5 mg **(**88%). Colorless oil.

^{1}H NMR (300 MHz, $CDCl_3$): δ 10.03 (s, 1H), 7.91 – 7.86 (m, 2H), 7.68 – 7.60 (m, 1H), 7.57 – 7.50 (m, 2H).

^{13}C NMR (75 MHz, $CDCl_3$): δ 192.3, 136.4, 134.4, 129.7, 129.0.

Spectroscopic data are in agreement with the reported values in the literature.[7]

Benzophenone (16) (Table 2, entry 2)

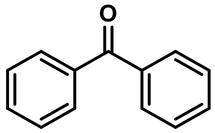

16

Yield: 29.7 mg (68%). White solid.

^{1}H NMR (300 MHz, $CDCl_3$): δ 7.84 – 7.78 (m, 4H), 7.59 (ddt, *J* = 8.4, 6.6, 1.4 Hz, 2H), 7.52 – 7.45 (m, 4H).

^{13}C NMR (75 MHz, $CDCl_3$): δ 196.69, 137.59, 132.36, 130.02, 128.24.

Spectroscopic data are in agreement with the reported values in the literature.[8]

Nonanal (18) (Table 2, entry 3)

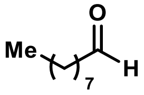

18

Yield: 11.3 mg **(**66%). Colorless oil.

^{1}H NMR (300 MHz, $CDCl_3$): δ 9.76 (t, *J* = 1.9 Hz, 1H), 2.41 (td, *J* = 7.4, 1.9 Hz, 2H), 1.63 (dd, *J* = 9.4, 5.3 Hz, 2H), 1.36 – 1.21 (m, 10H), 0.92 – 0.84 (m, 3H).

^{13}C NMR (75 MHz, $CDCl_3$): δ 202.91, 43.90, 31.77, 29.29, 29.16, 29.07, 22.64, 22.61, 22.08, 14.05.

Spectroscopic data are in agreement with the reported values in the literature.[9]

Benzaldehyde (14) (Table 2, entry 4)

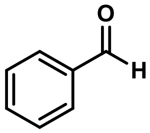

14

Yield: 11.3 mg (66%). Colorless oil.

2-((1*S*,3*S*)-3-acetyl-2,2-dimethylcyclobutyl)acetaldehyde (21) (Table 2, entry 5)

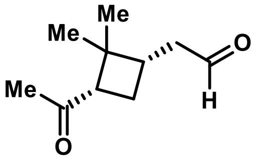

21

Yield: 19 mg **(**94%). Yellowish oil.

^{1}H NMR (300 MHz, $CDCl_3$): δ 9.74 (t, *J* = 1.5 Hz, 1H), 2.92 (dd, *J* = 9.9, 7.8 Hz, 1H), 2.51 – 2.36 (m, 3H), 2.04 (s, 3H), 2.01 – 1.92 (m, 2H), 1.34 (s, 3H), 0.84 (s, 3H).

^{13}C NMR (75 MHz, $CDCl_3$): δ 207.31, 201.33, 54.34, 45.11, 43.26, 35.78, 30.34, 30.13, 22.82, 17.63.

$[\boldsymbol{\alpha}]_{\boldsymbol{D}}^{\mathbf{20}}$ (CH_2Cl_2): + 61° (Lit. + 40°).[10]

Spectroscopic data are in agreement with the reported values in the literature.[11]

9. References

[1] H. Yueh, A. Voevodin and A. B. Beeler, *J. Flow Chem.* 2015, **5**, 155–159.

[2] B. A. Hess, A. S. Bailey, B. Bartusek and V. Boekelheide, *J. Am. Chem. Soc.* 1969, **91**, 1665–1672.

[3] A. R. Davalos, E. Sylvester and S. T. Diver, *Organometallics* 2019, **38**, 2338–2346.

[4] T. Louis-Goff, A. L. Rheingold and J. Hyvl, *Organometallics* 2020, **39**, 778–782.

[5] A. Buhling, P. C. J. Kamer and P. W. N. M. van Leeuwen, *Organometallics* 1997, **16**, 3027–3037.

[6] D. H. R. Barton, J.-P. Finet, W. B. Motherwell and C. Pichon, *Tetrahedron* 1986, **42**, 5627–5636.

[7] E. Prathibha, R. Rangasamy, A. Sridhar and K. Lakshmi, *ChemistrySelect* 2020, **5**, 988–993.

[8] Z. Shen, Z. Zhao, Y.-L. Ren, W. Liu, X. Tian, X. Zheng and B. Zhao, *ChemistrySelect* 2020, **5**, 14288–14291.

[9] S. Wertz and A. Studer, *Adv. Synth. Catal.* 2011, **353**, 69–72.

[10] H. E. Eschinazi, *J. Am. Chem. Soc.* 1959, **81**, 2905–2906.

[11] A. V. Iosub, S. Moravcik, C.-J. Wallentin and J. Bergman, *Org. Lett.* 2019, **21**, 7804–7808.

10. NMR spectra

^{1}H NMR (300 MHz, $CDCl_3$) of **3**

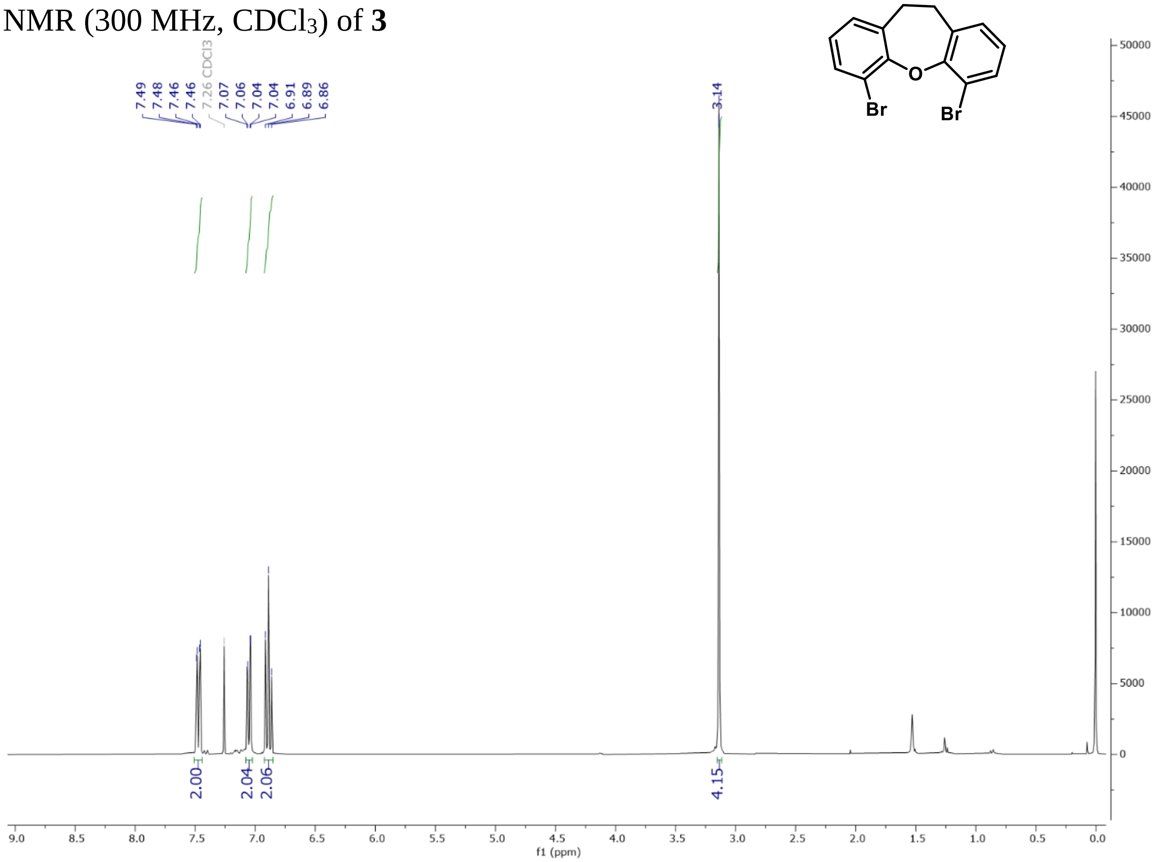

^{13}C NMR (75 MHz, $CDCl_3$) of **3**

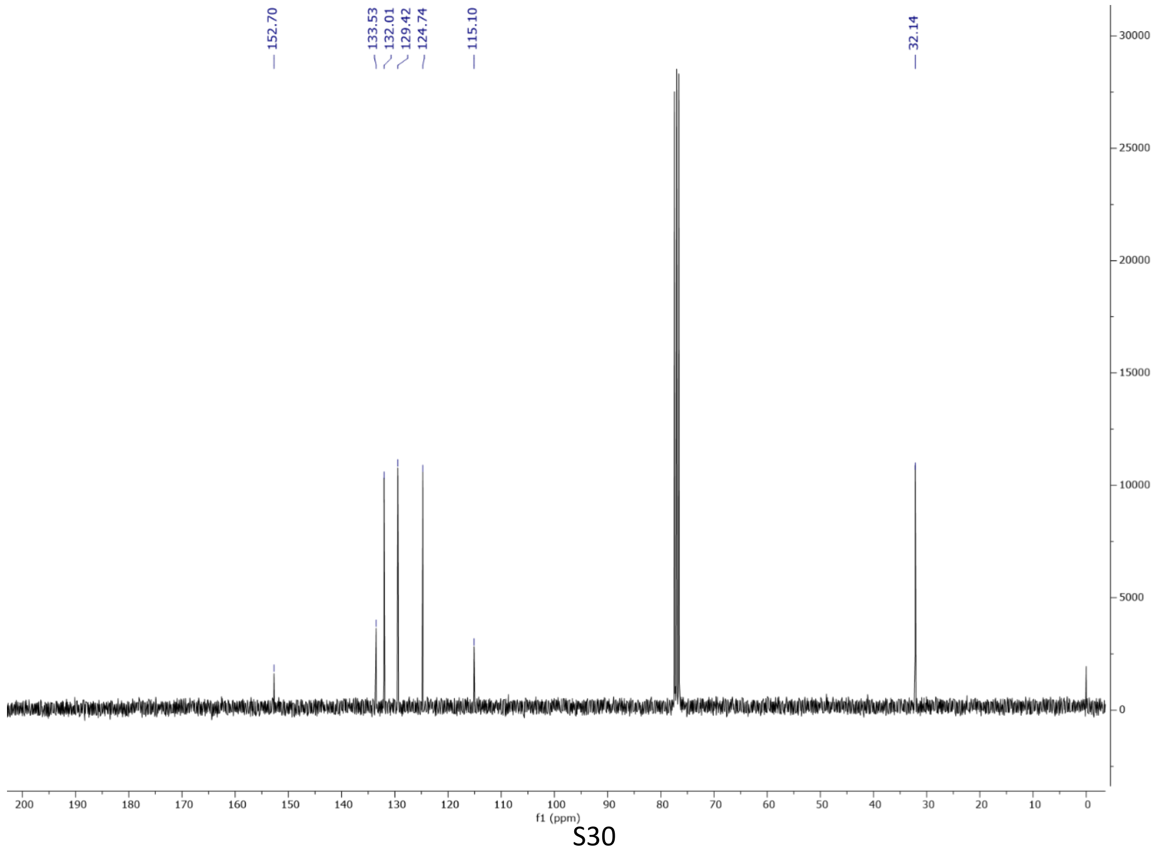

^{1}H NMR (300 MHz, $CDCl_3$) of **4**

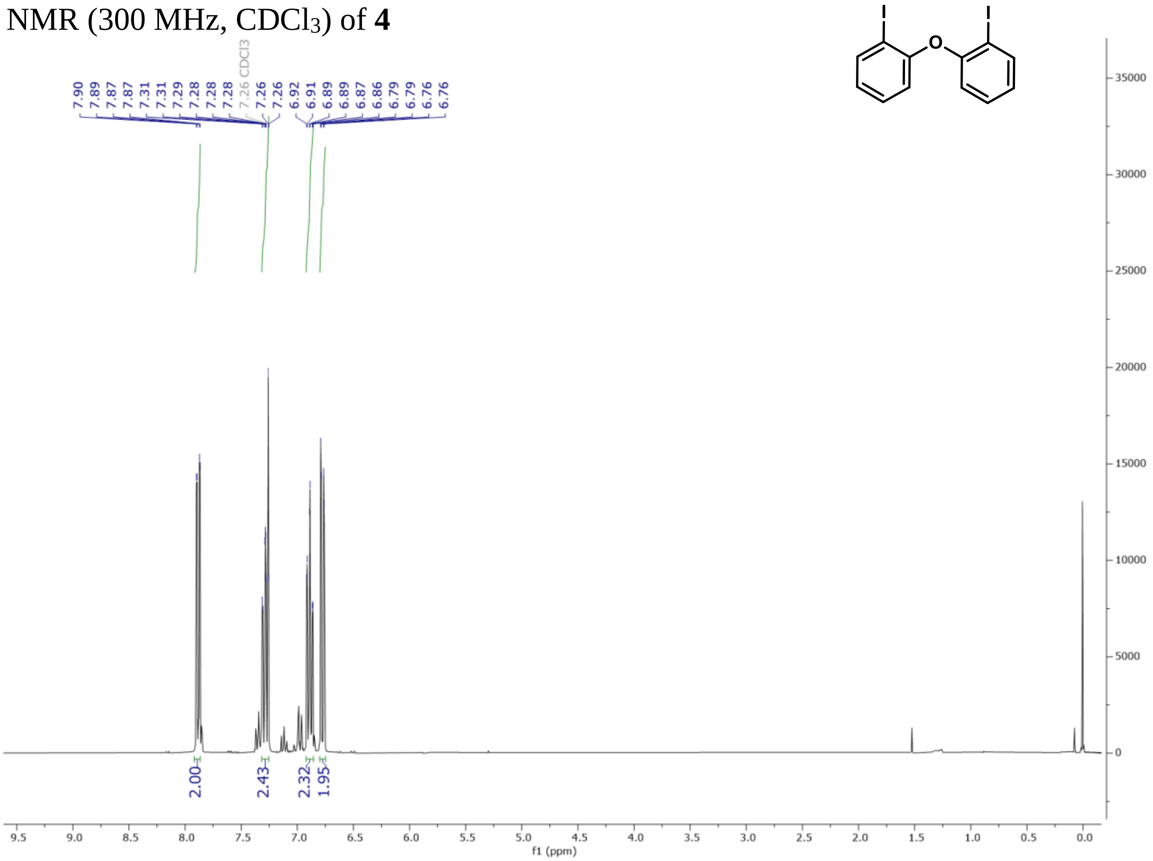

^{13}C NMR (75 MHz, $CDCl_3$) of

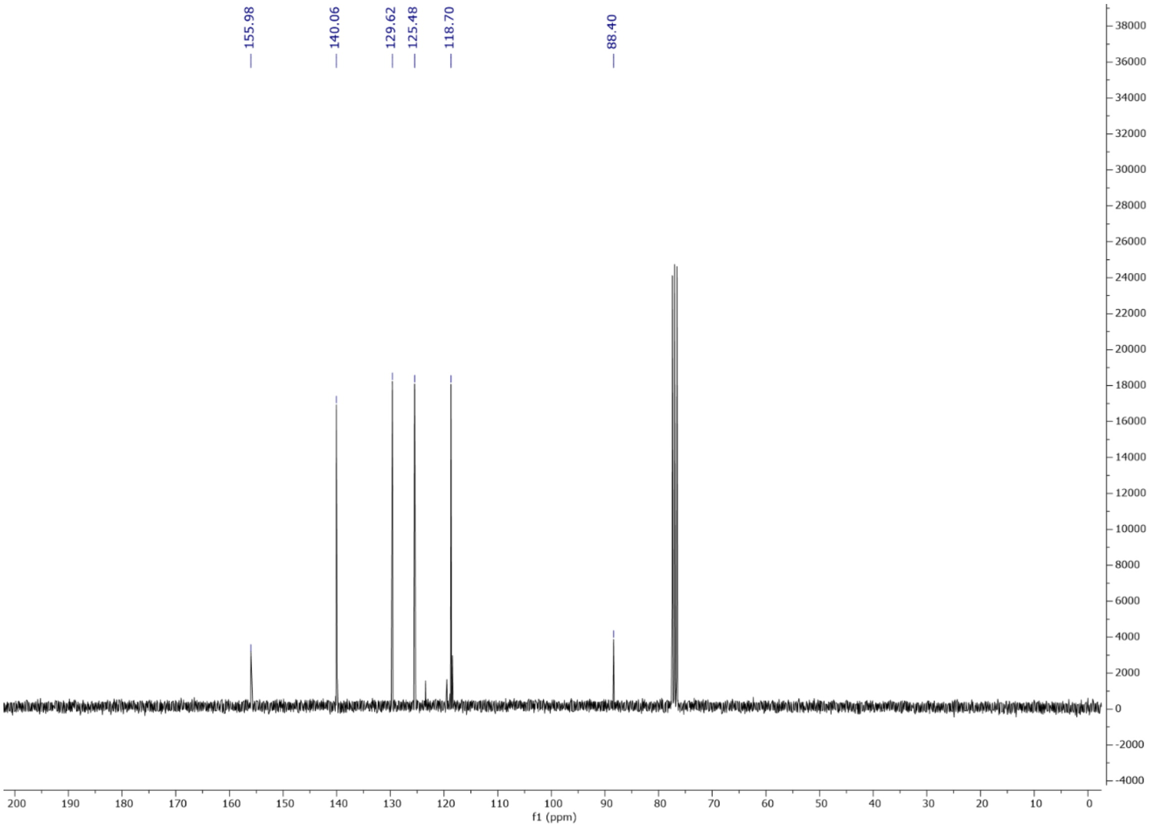

^{1}H NMR (300 MHz, $CDCl_3$) of **5**

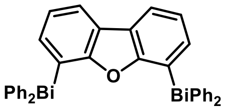

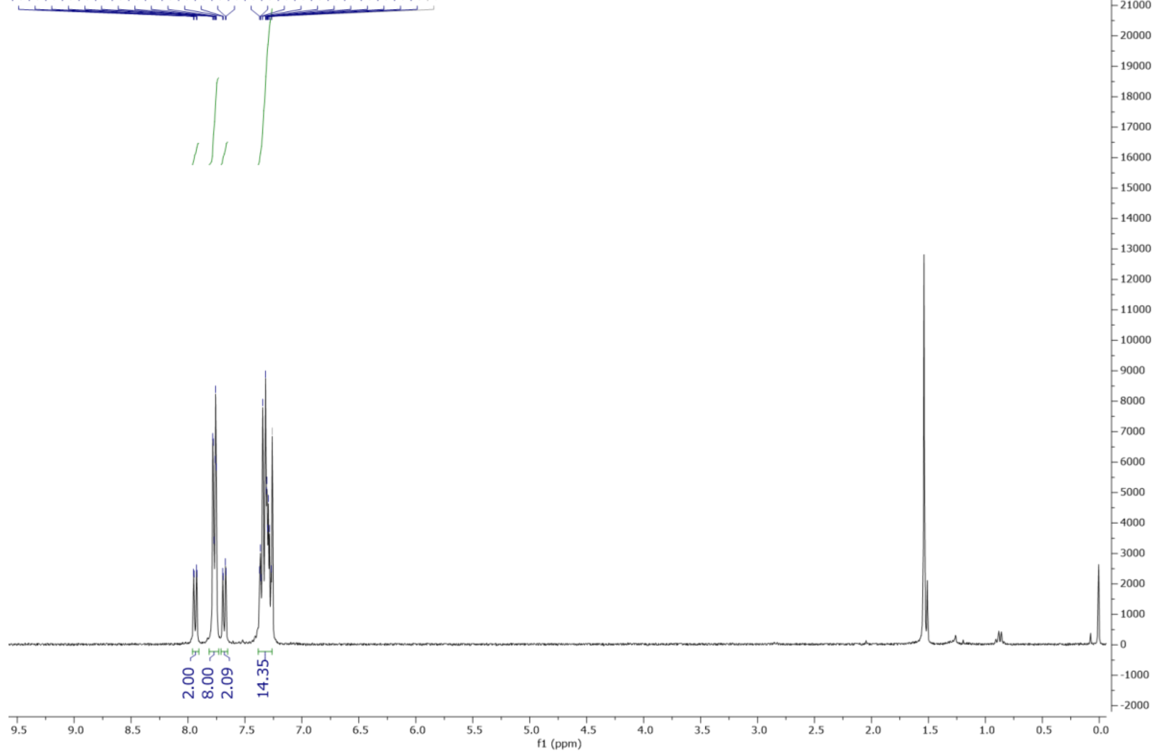

^{13}C NMR (75 MHz, $CDCl_3$) of **5**

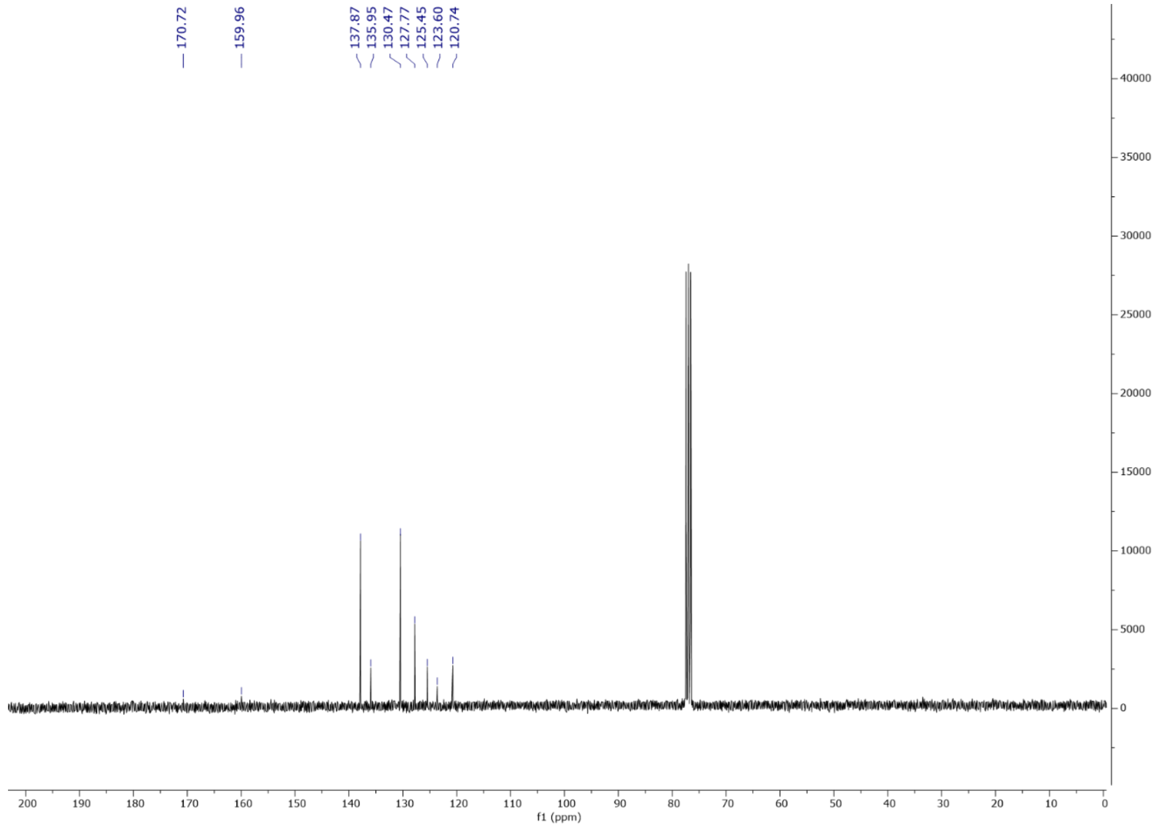

^{1}H NMR (300 MHz, $CDCl_3$) of **6**

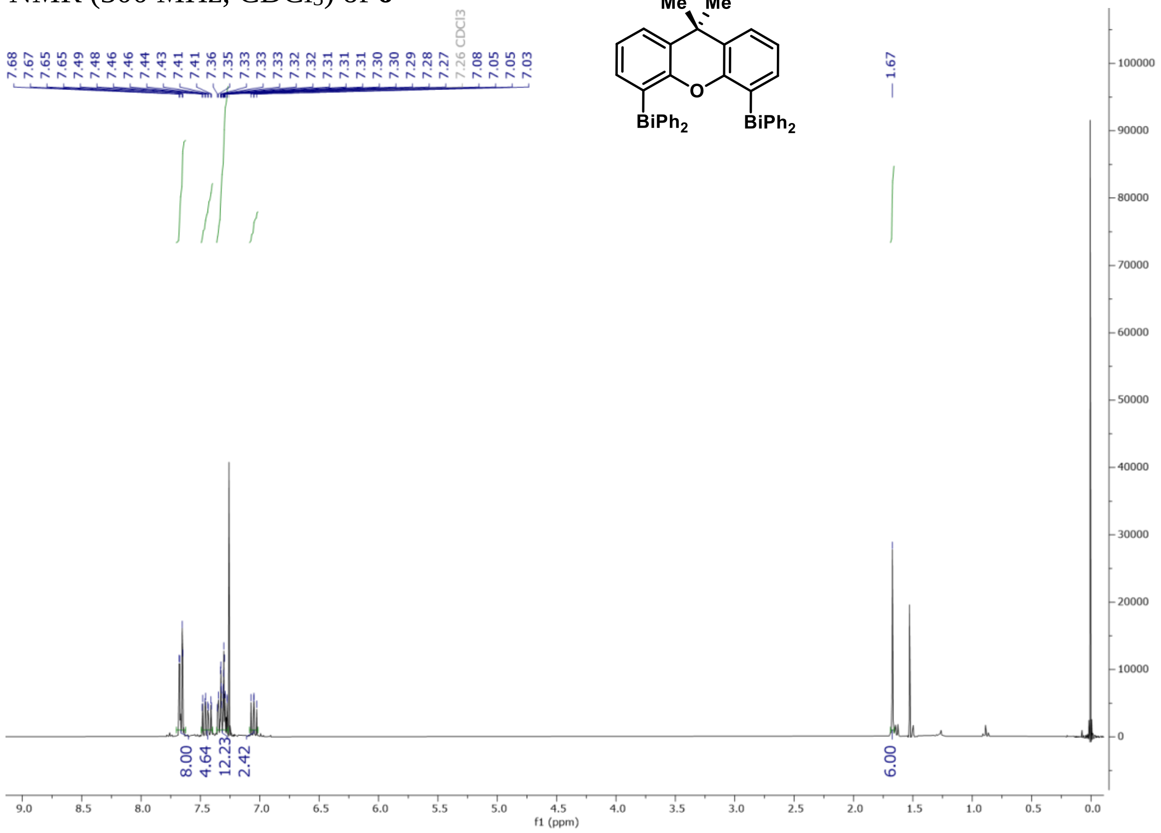

^{13}C NMR (75 MHz, $CDCl_3$) of **6**

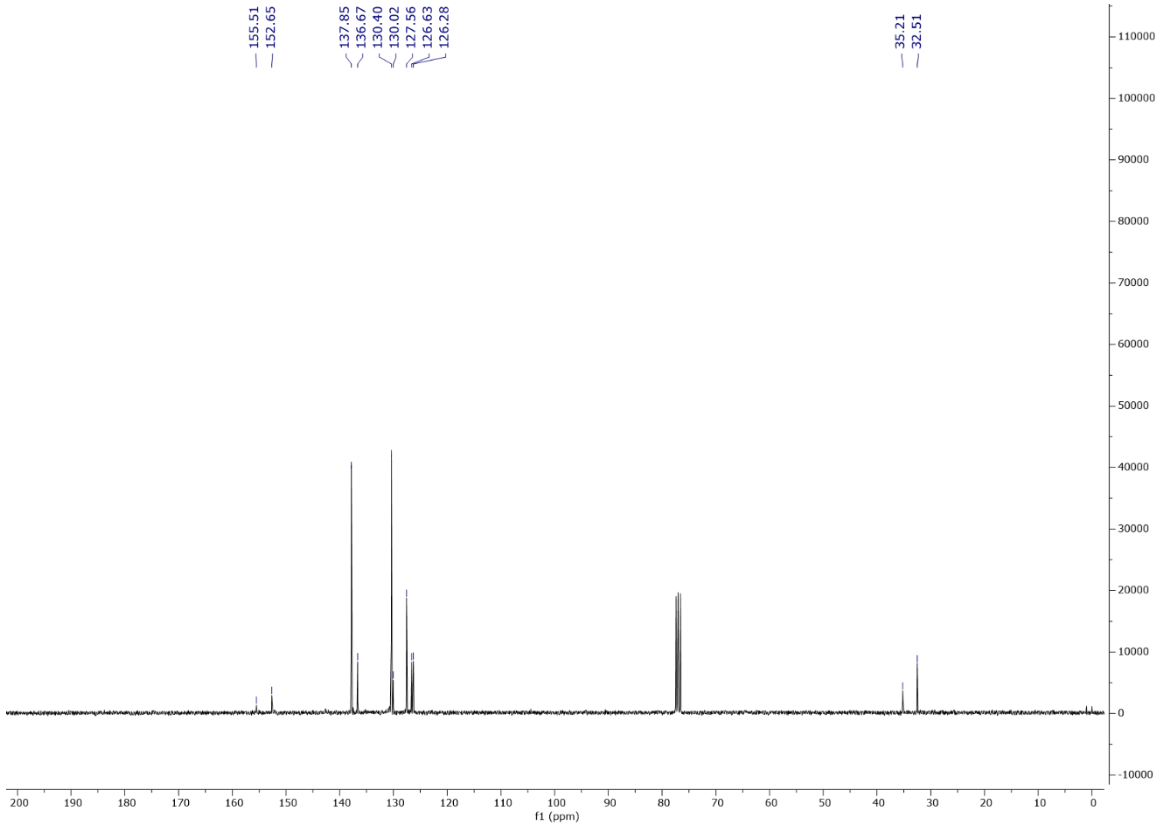

^{1}H NMR (300 MHz, $CDCl_3$) of **7**

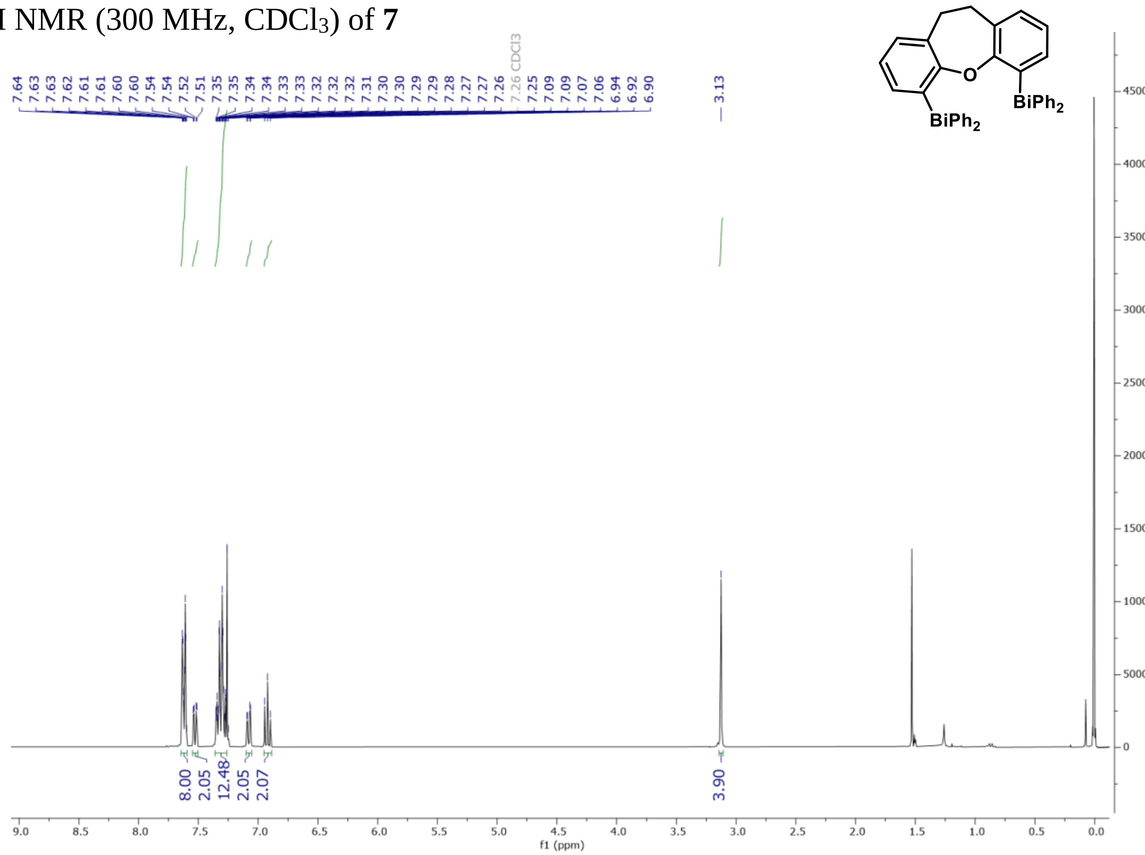

^{13}C NMR (75 MHz, $CDCl_3$) of **7**

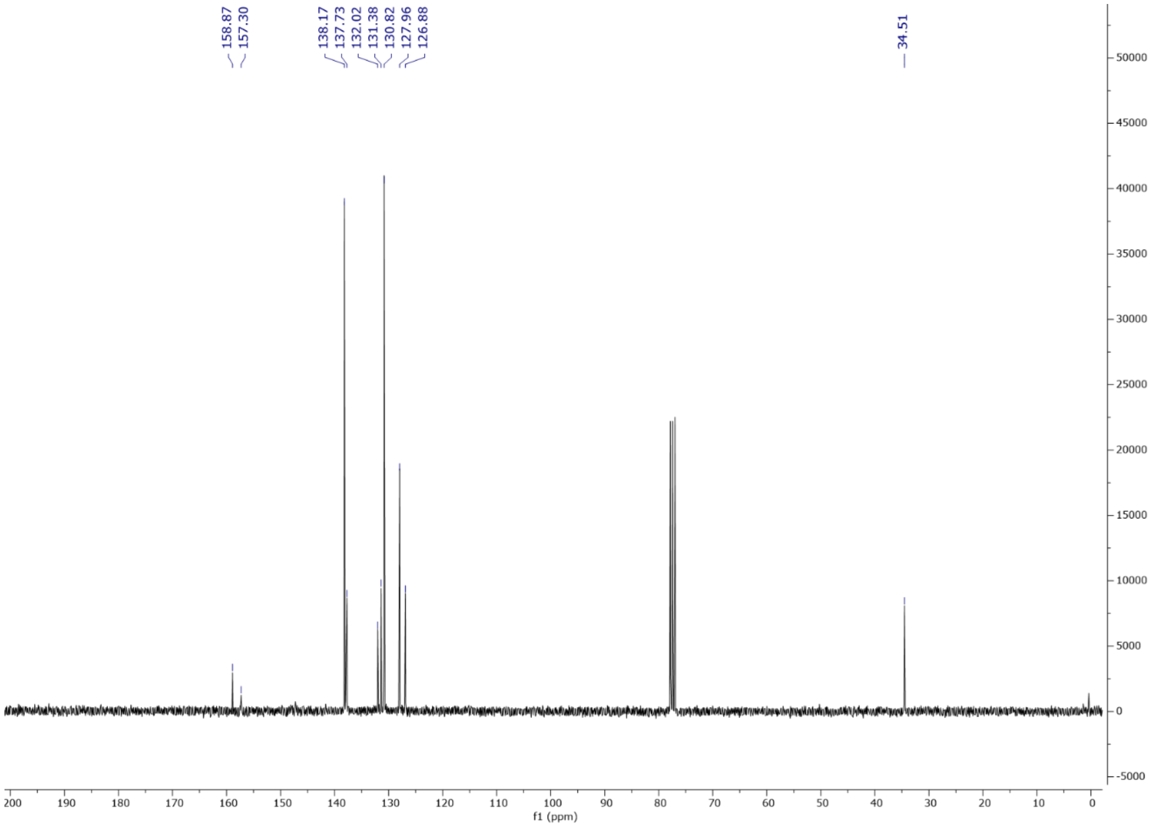

^{1}H NMR (400 MHz, $CDCl_3$) of **8**

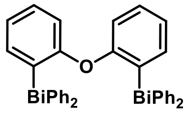

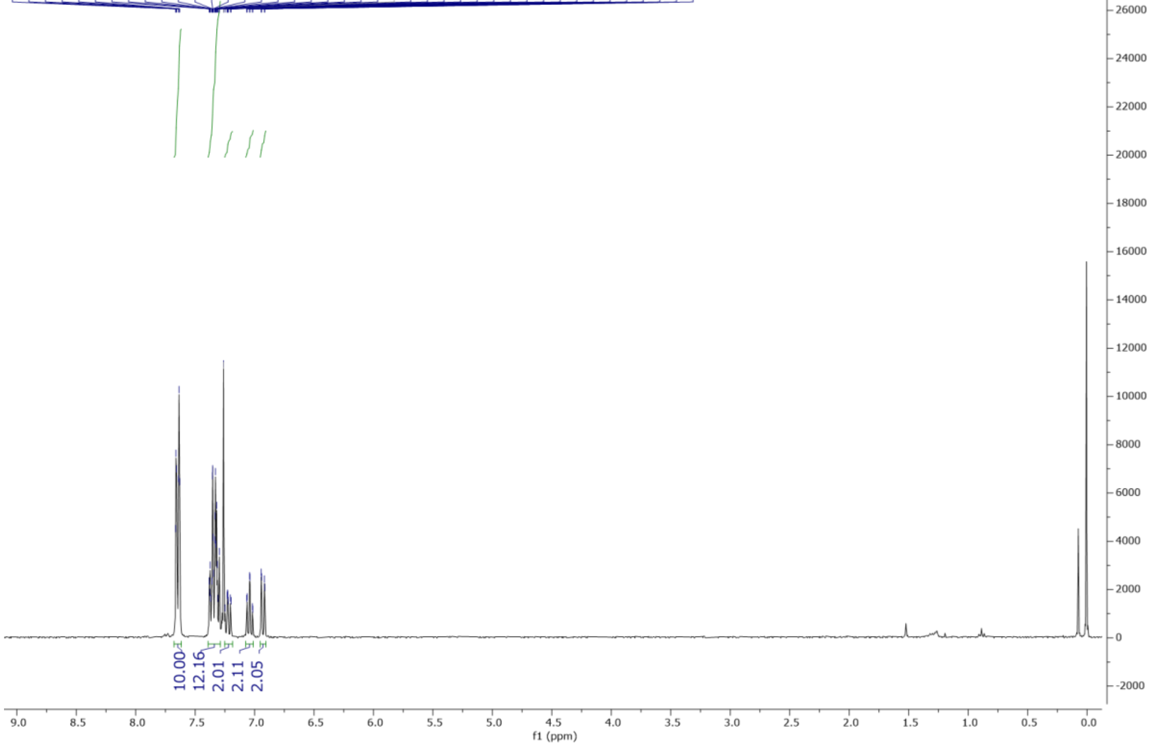

^{13}C NMR (101 MHz, $CDCl_3$) of **8**

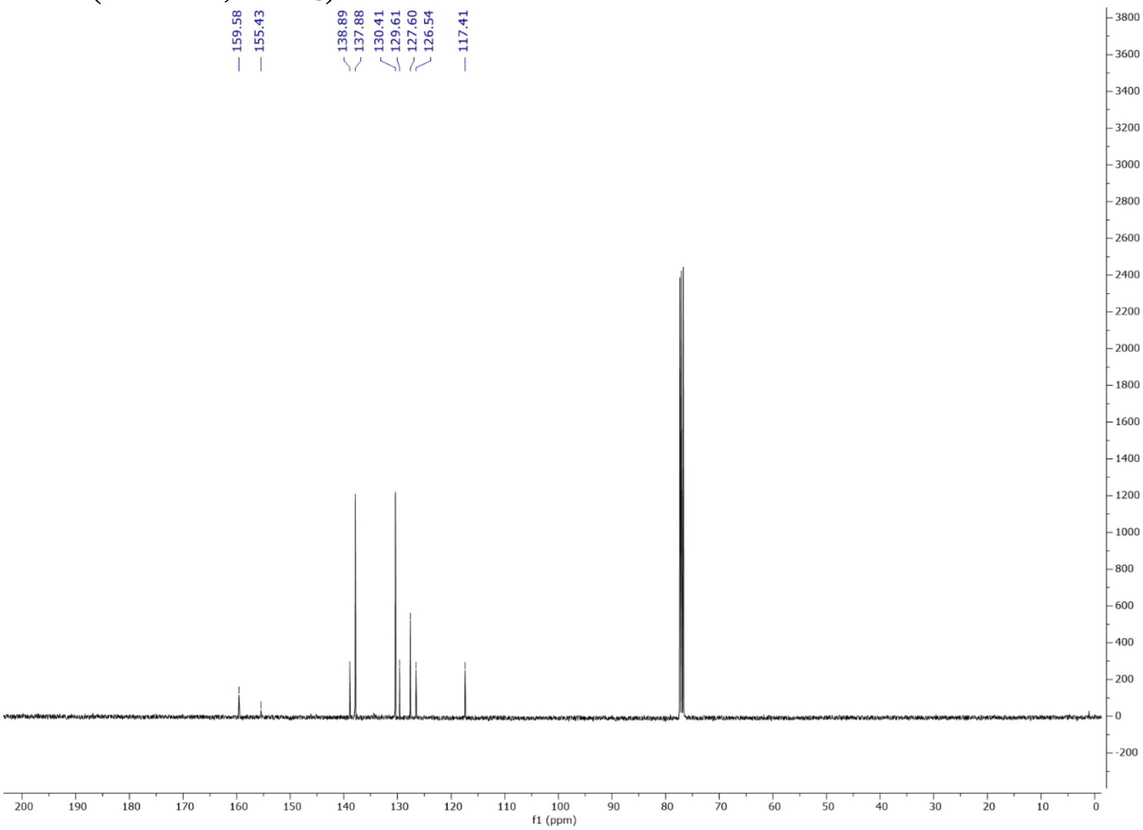

^{1}H NMR (400 MHz, $CDCl_3$) of **9**

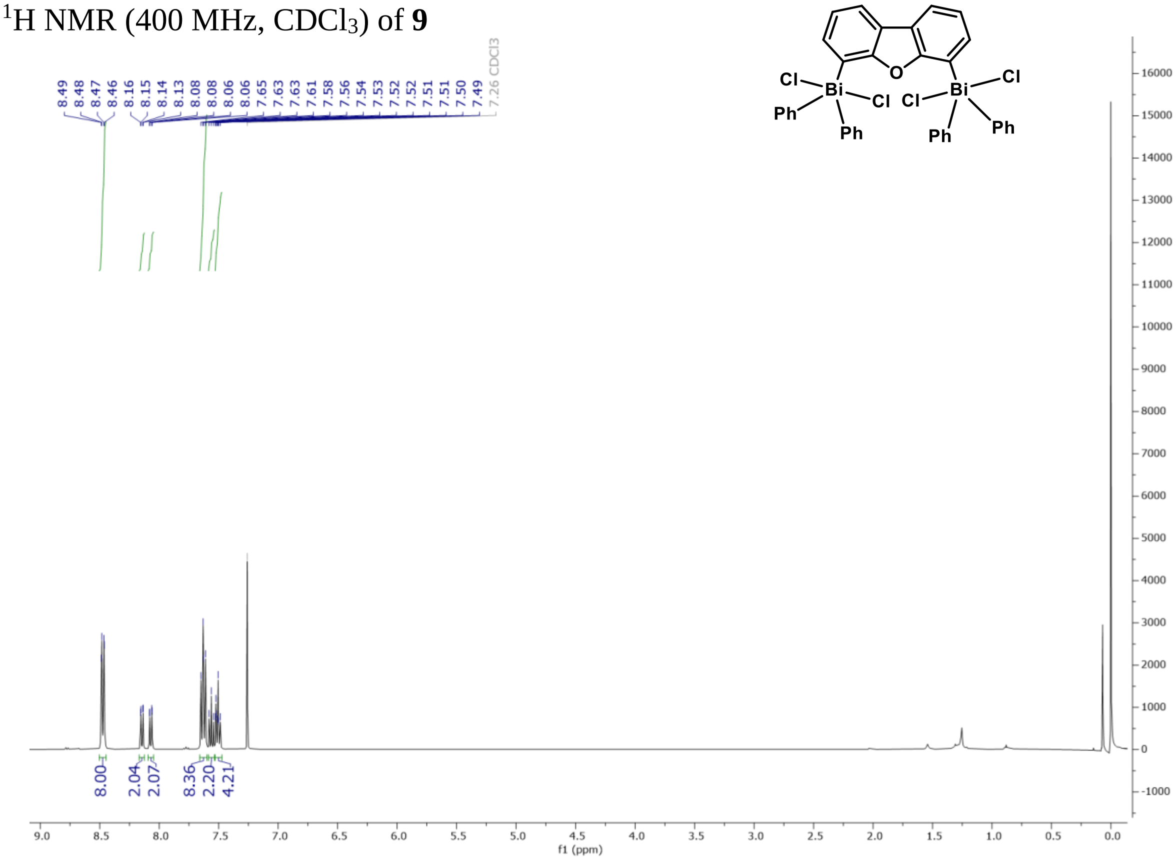

^{13}C NMR (101 MHz, $CDCl_3$) of **9**

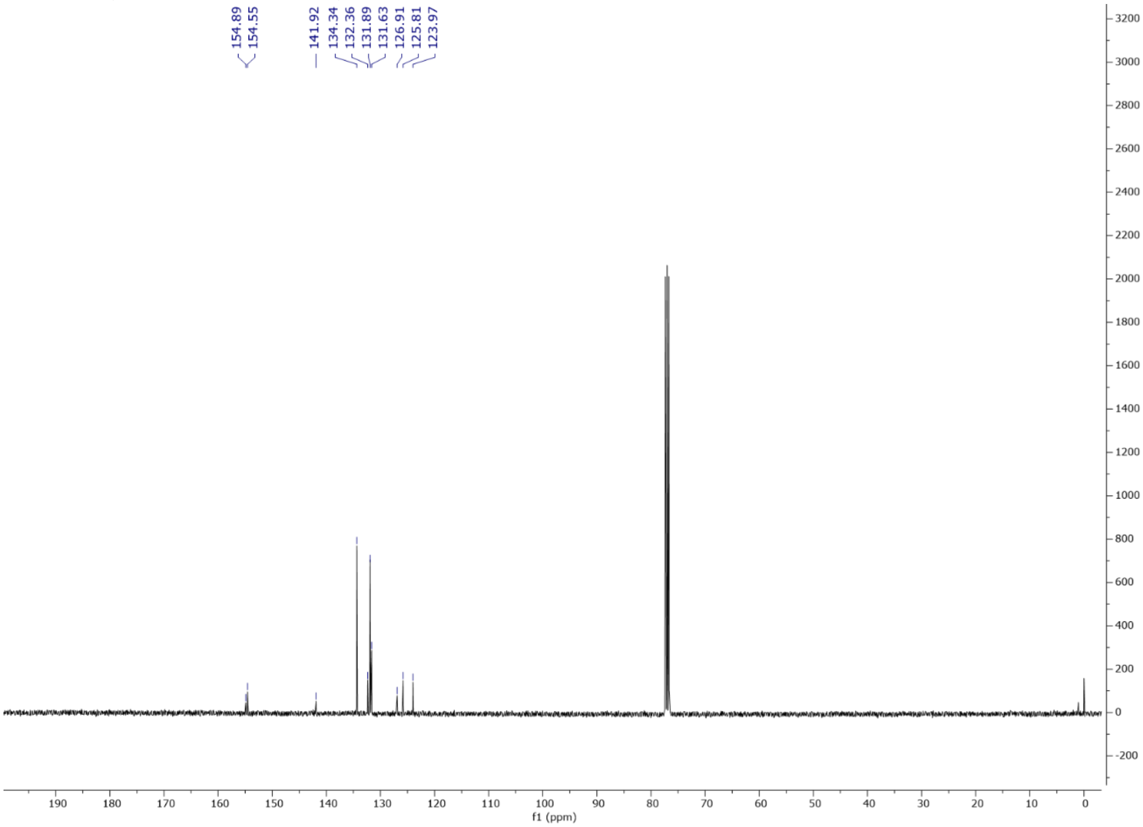

^{1}H NMR (300 MHz, $CDCl_3$) of **10**

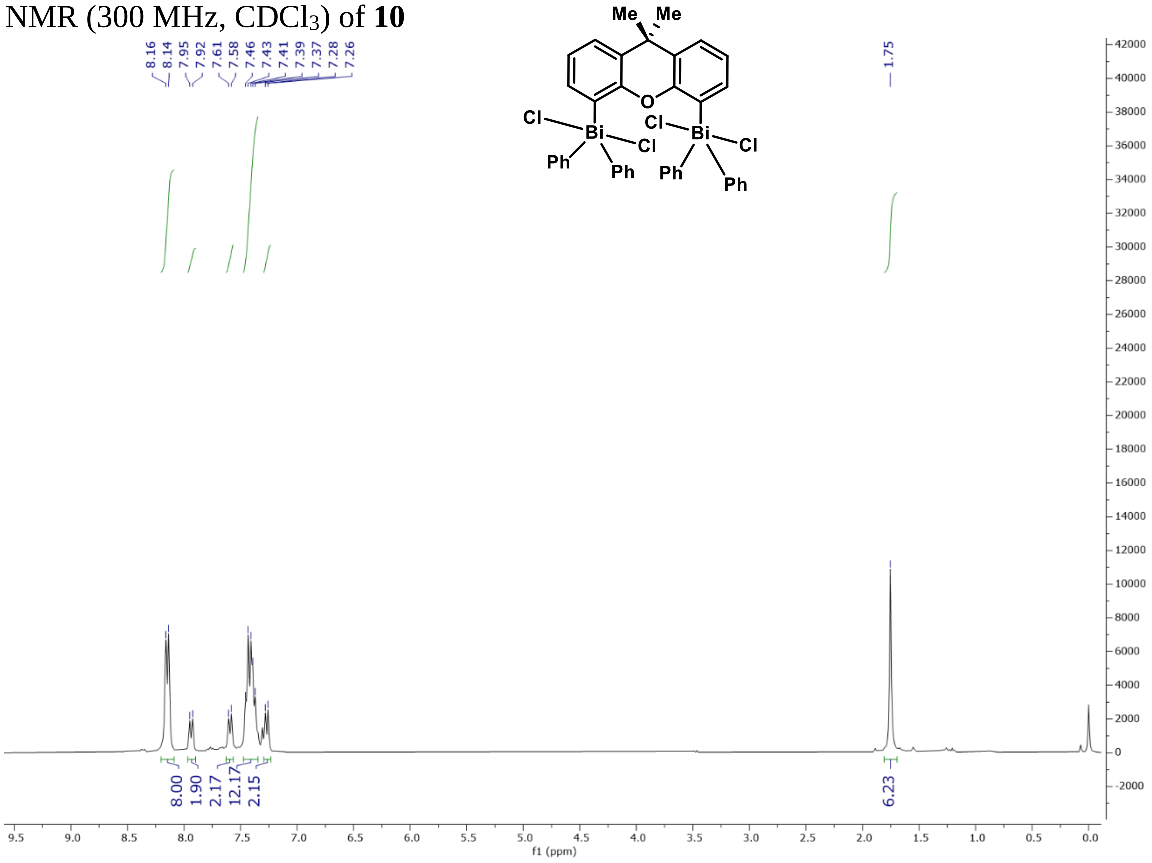

^{13}C NMR (75 MHz, $CDCl_3$) of **10**

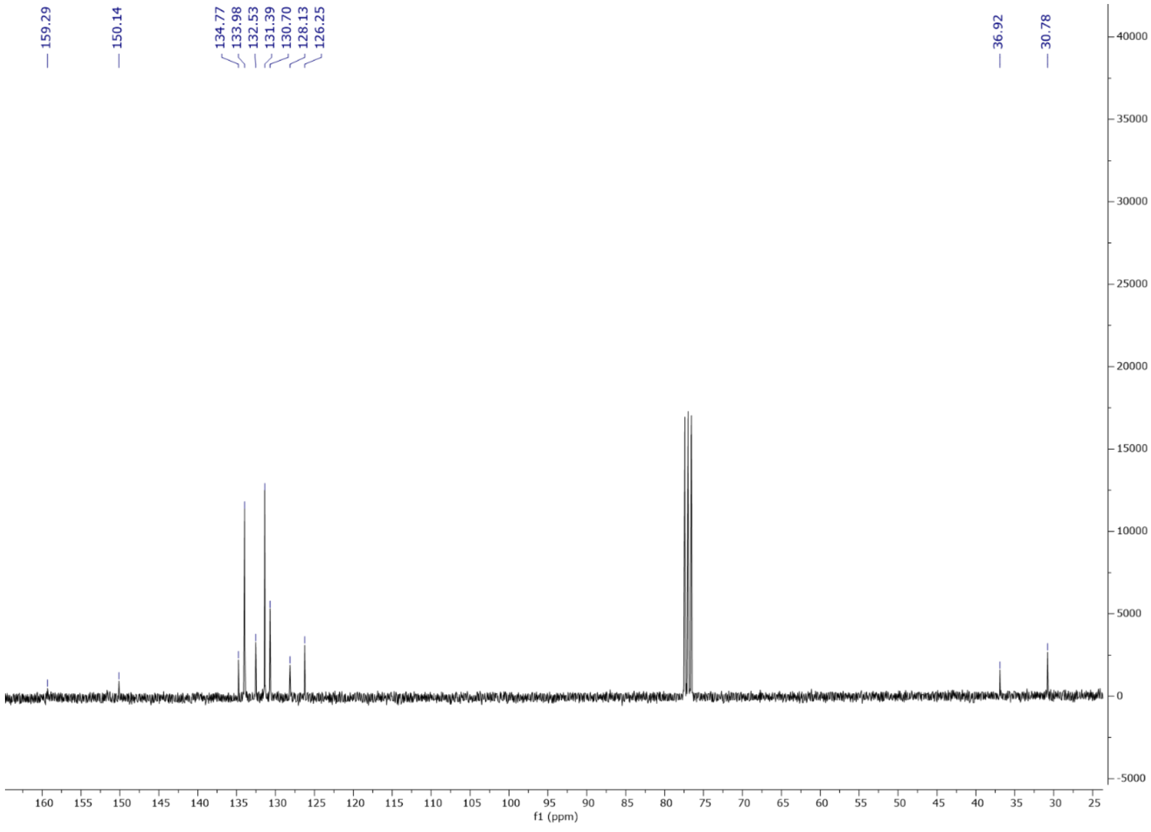

^{1}H NMR (400 MHz, $CDCl_3$) of **11**

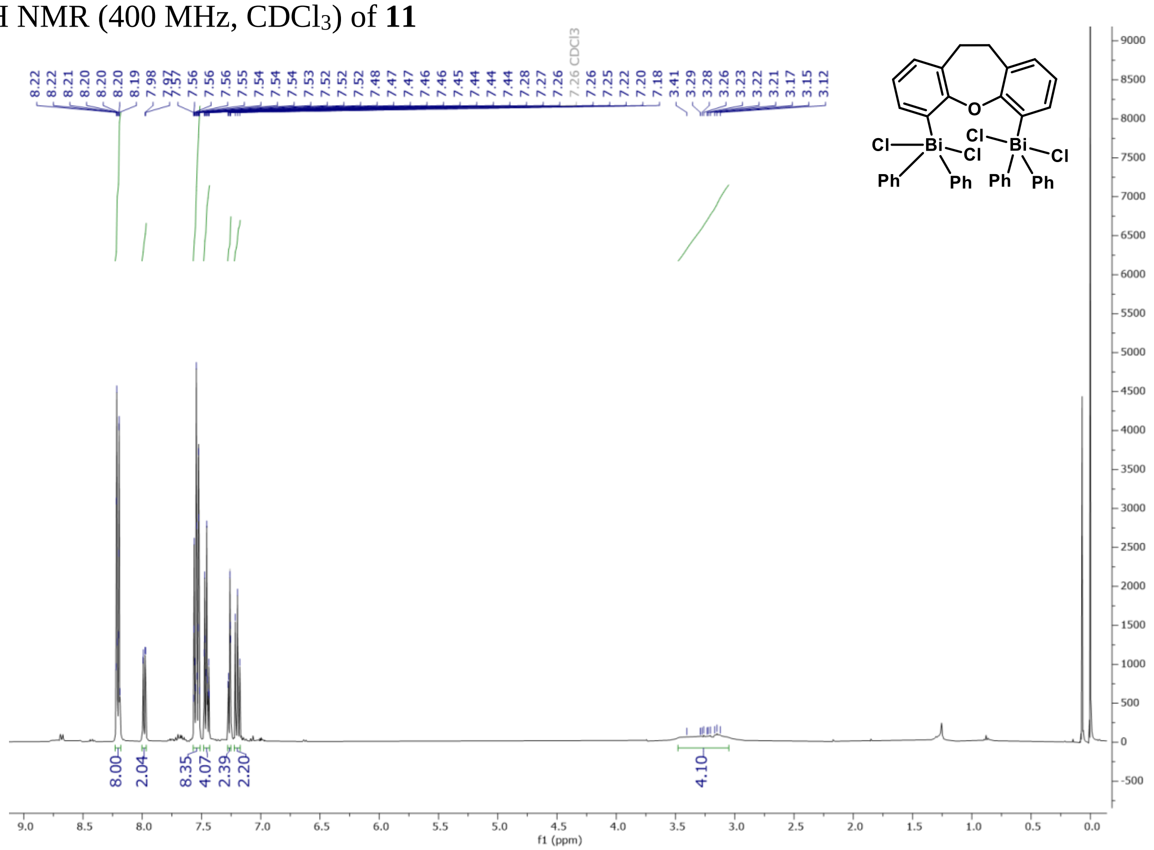

^{13}C NMR (101 MHz, $CDCl_3$) of **11**

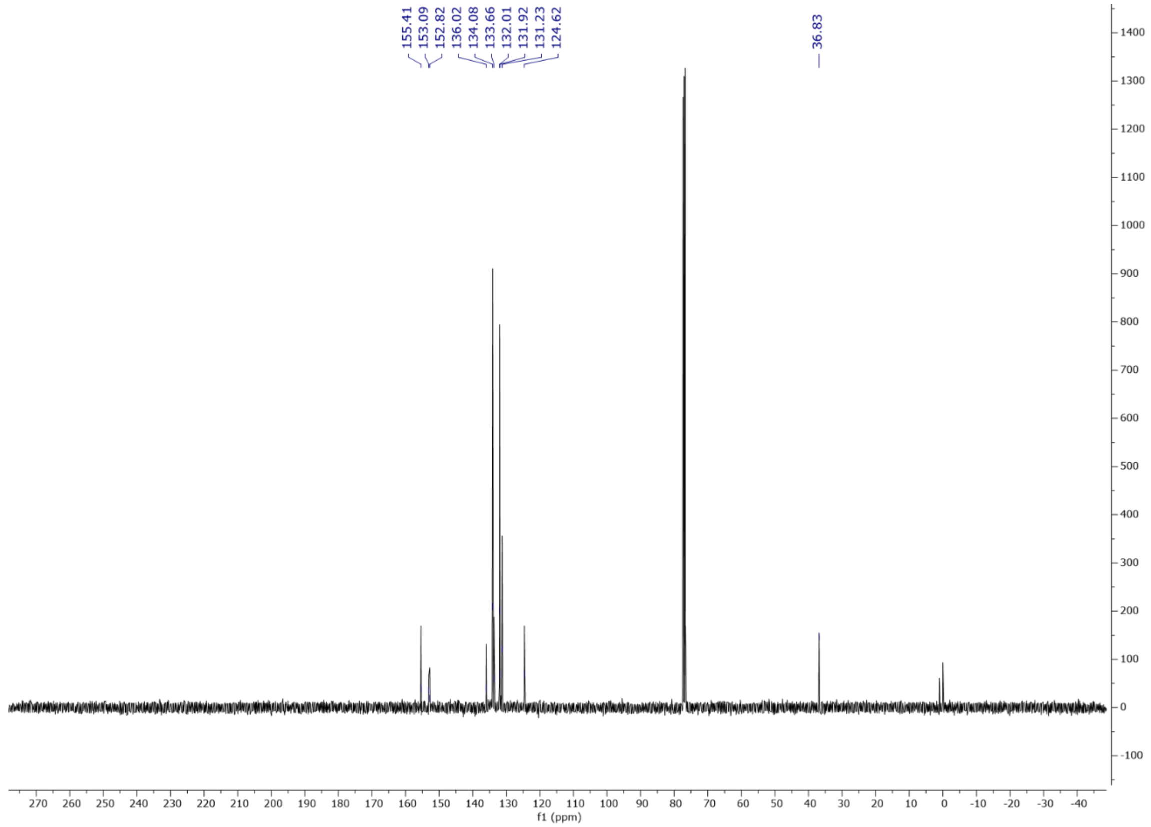

^{1}H NMR (400 MHz, $CDCl_3$) of **12**

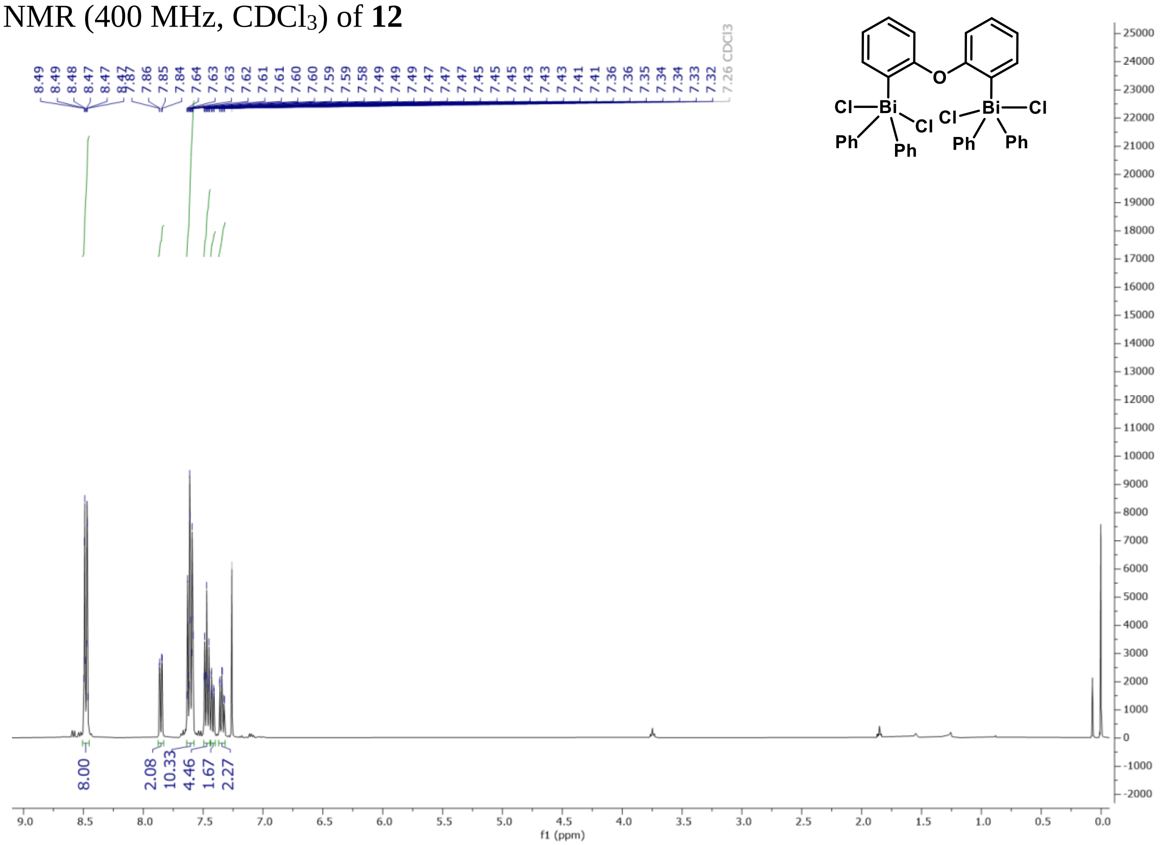

^{13}C NMR (101 MHz, $CDCl_3$) of **12**

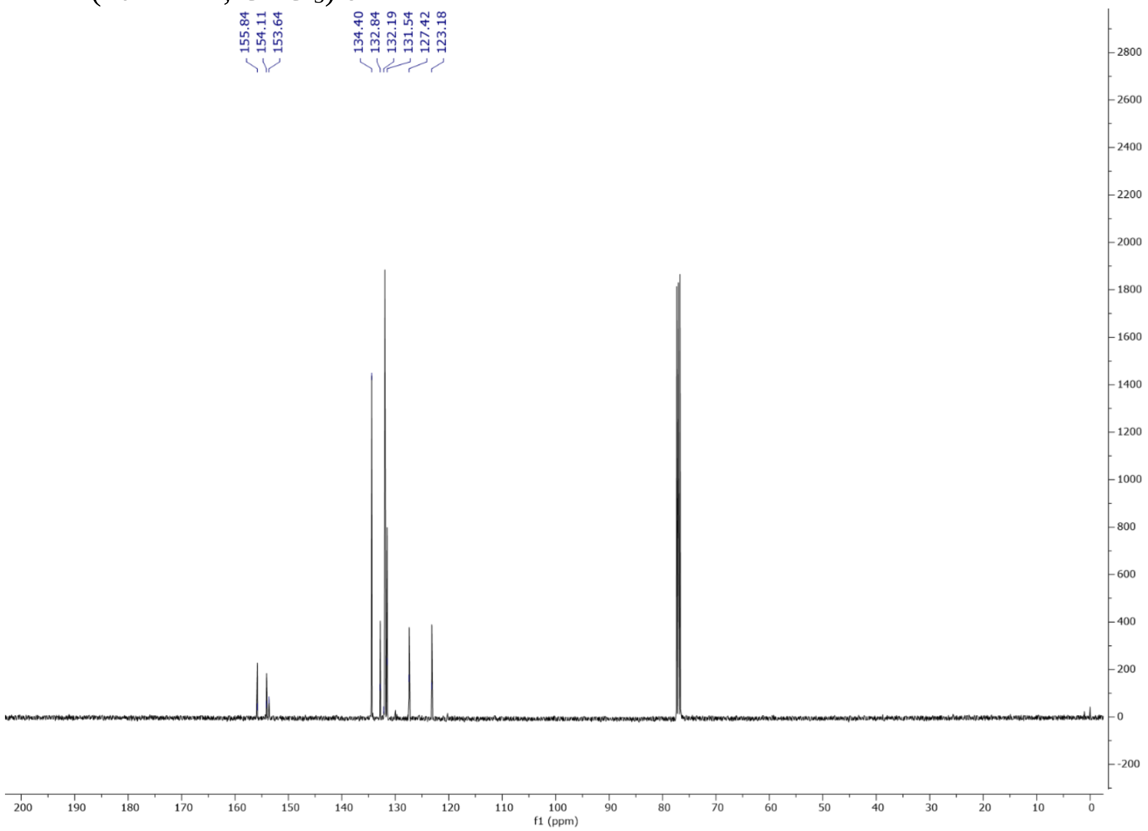

^{1}H NMR (300 MHz, $CDCl_3$) of **14**

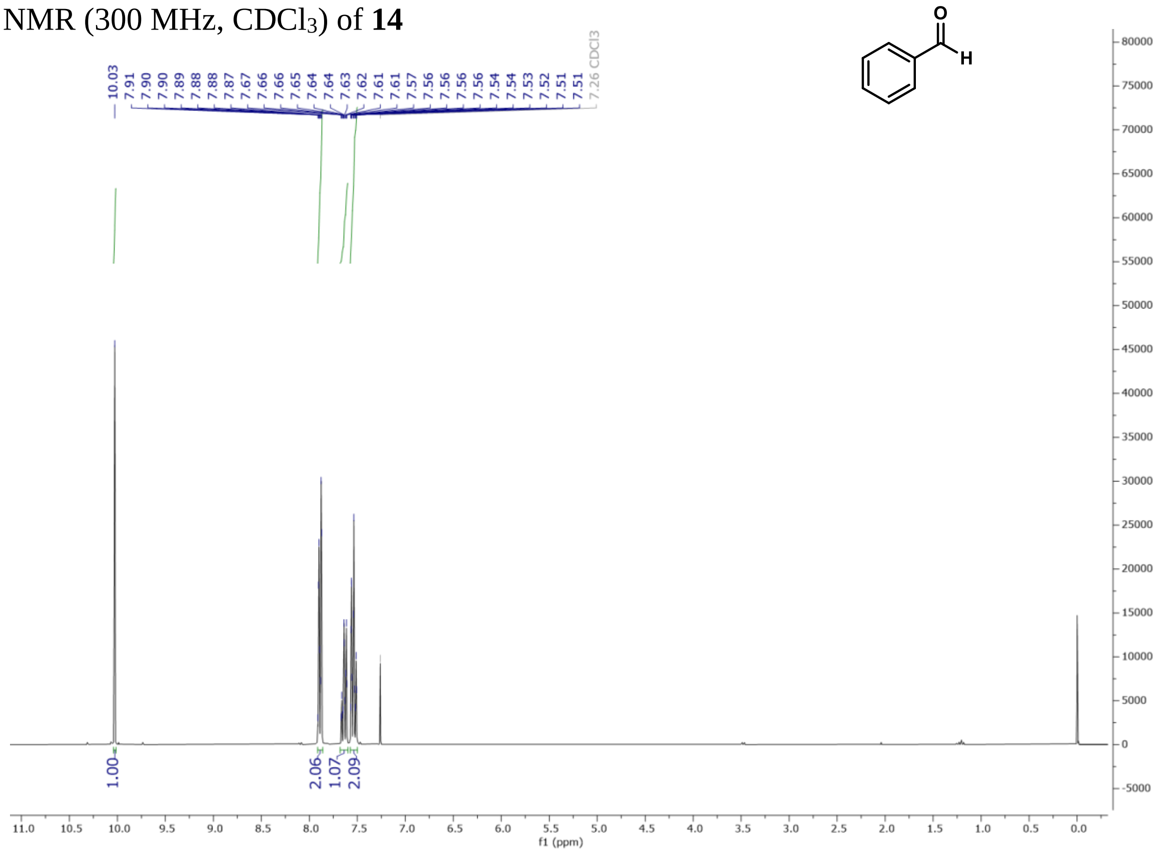

^{13}C NMR (75 MHz, $CDCl_3$) of **14**

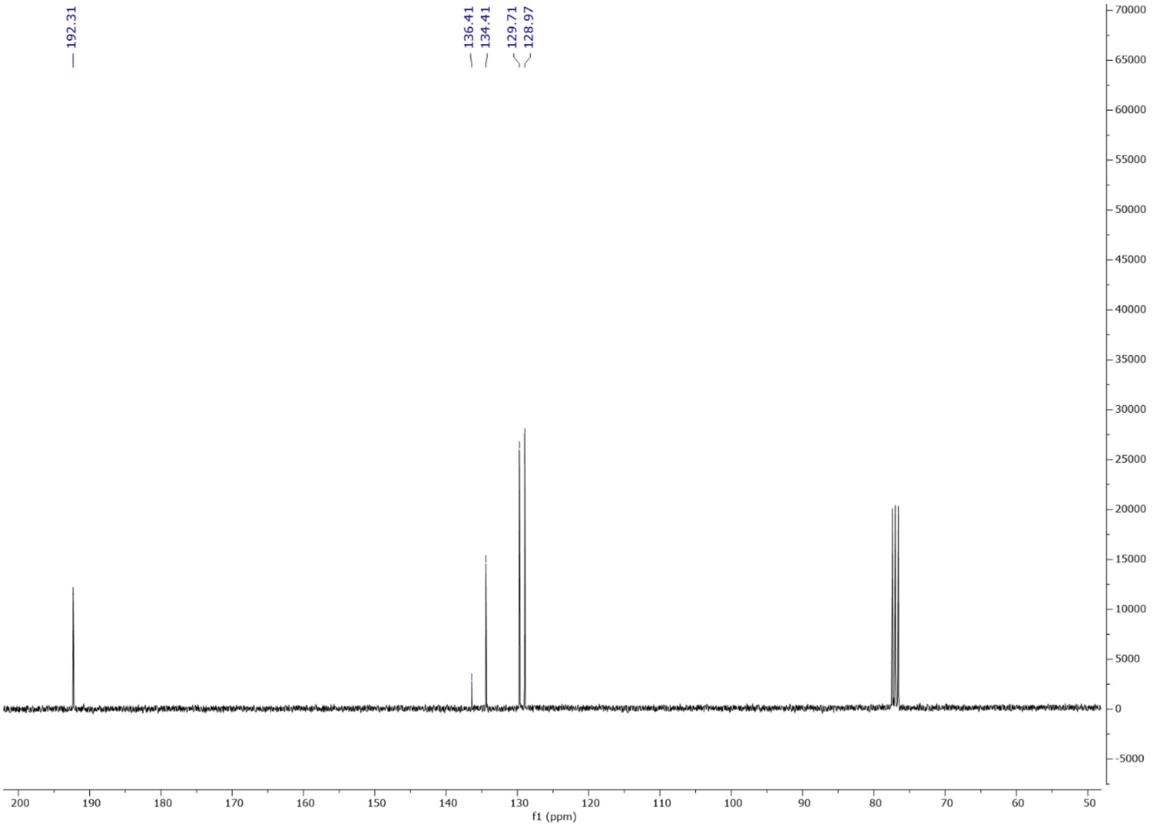

^{1}H NMR (300 MHz, $CDCl_3$) of **16**

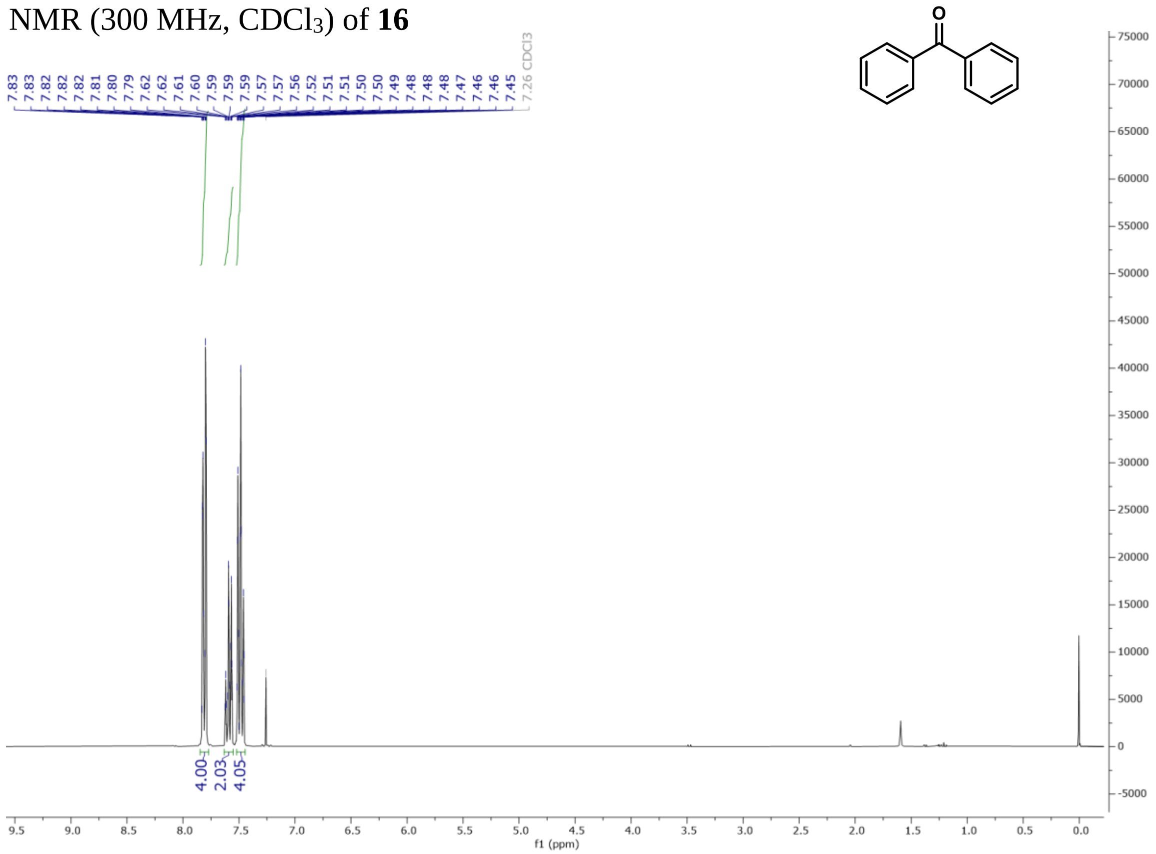

^{13}C NMR (75 MHz, $CDCl_3$) of **16**

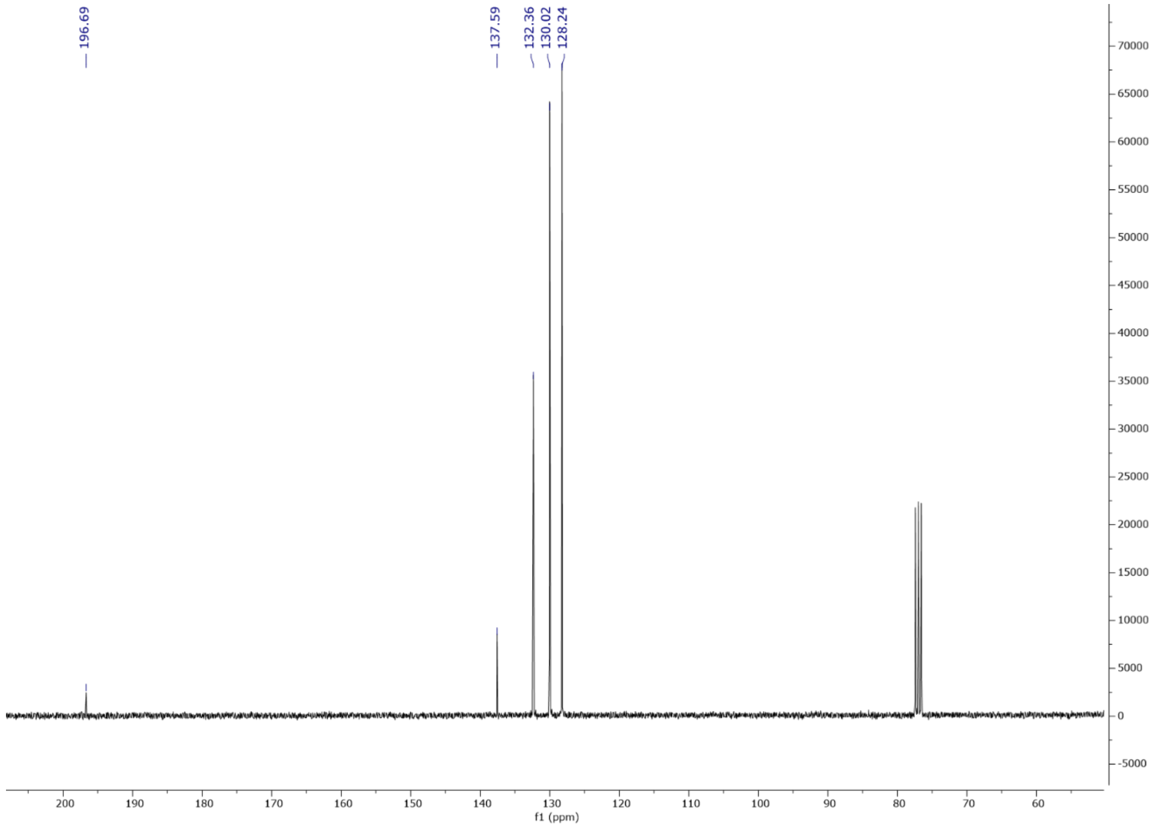

^{1}H NMR (300 MHz, $CDCl_3$) of **18**

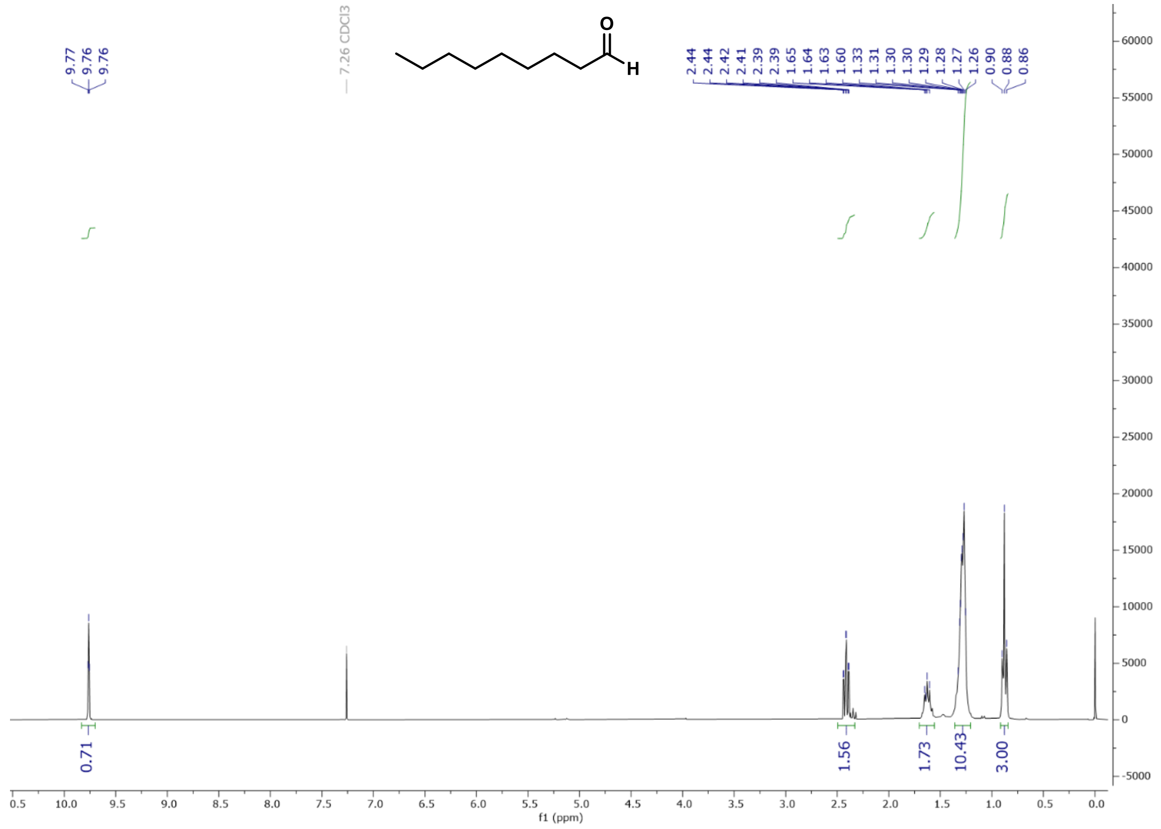

^{13}C NMR (75 MHz, $CDCl_3$) of **18**

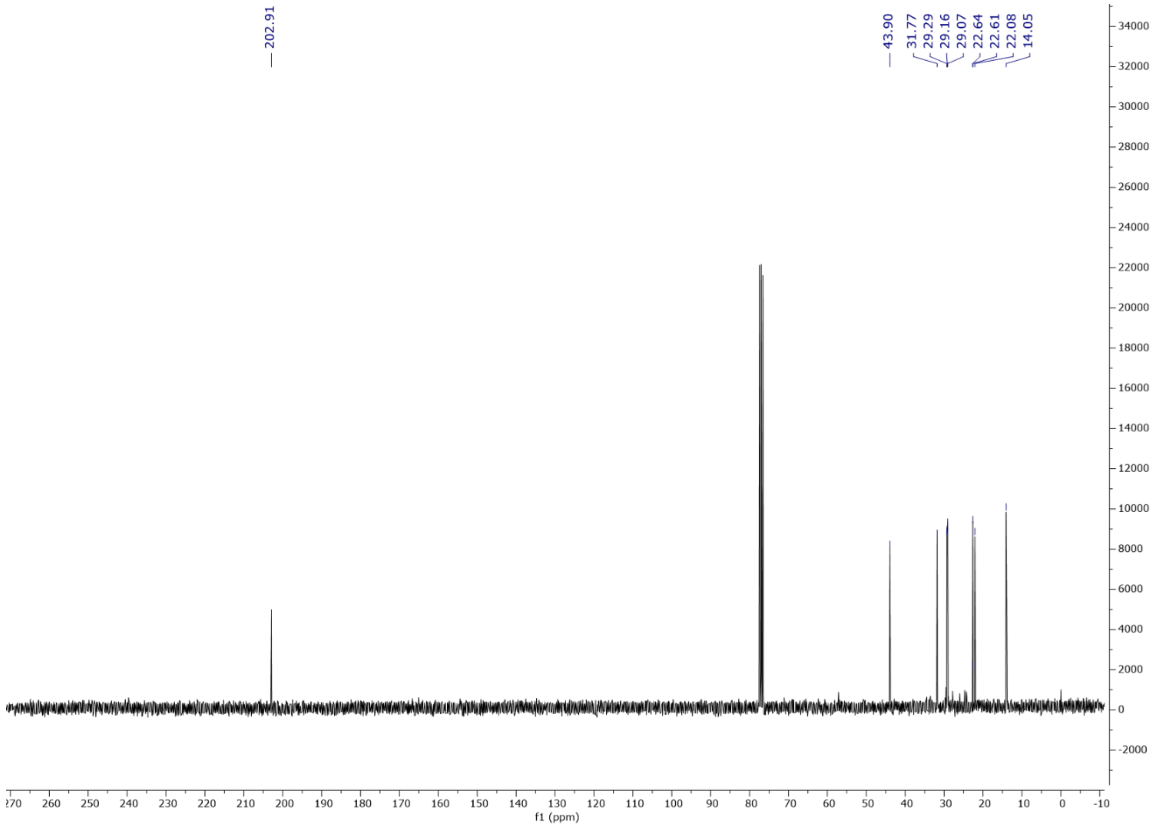

^{1}H NMR (300 MHz, $CDCl_3$) of **21**

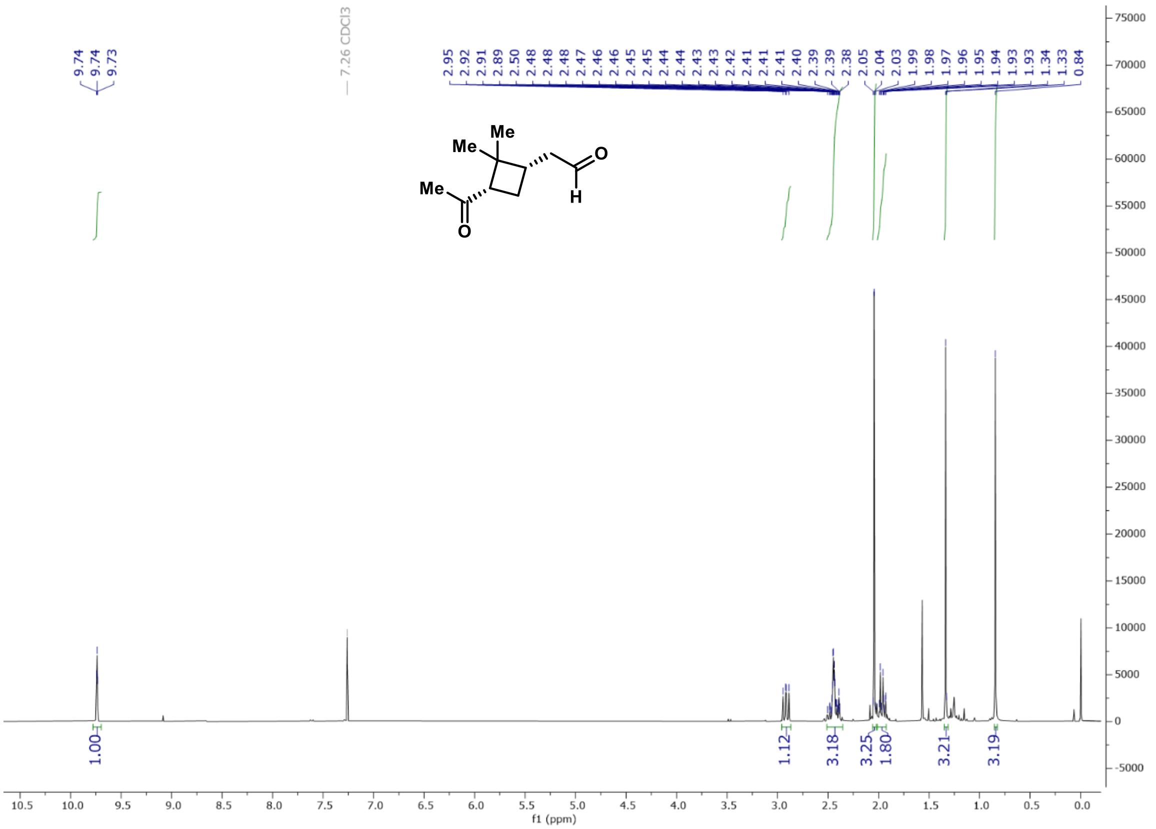

^{13}C NMR (75 MHz, $CDCl_3$) of **21**

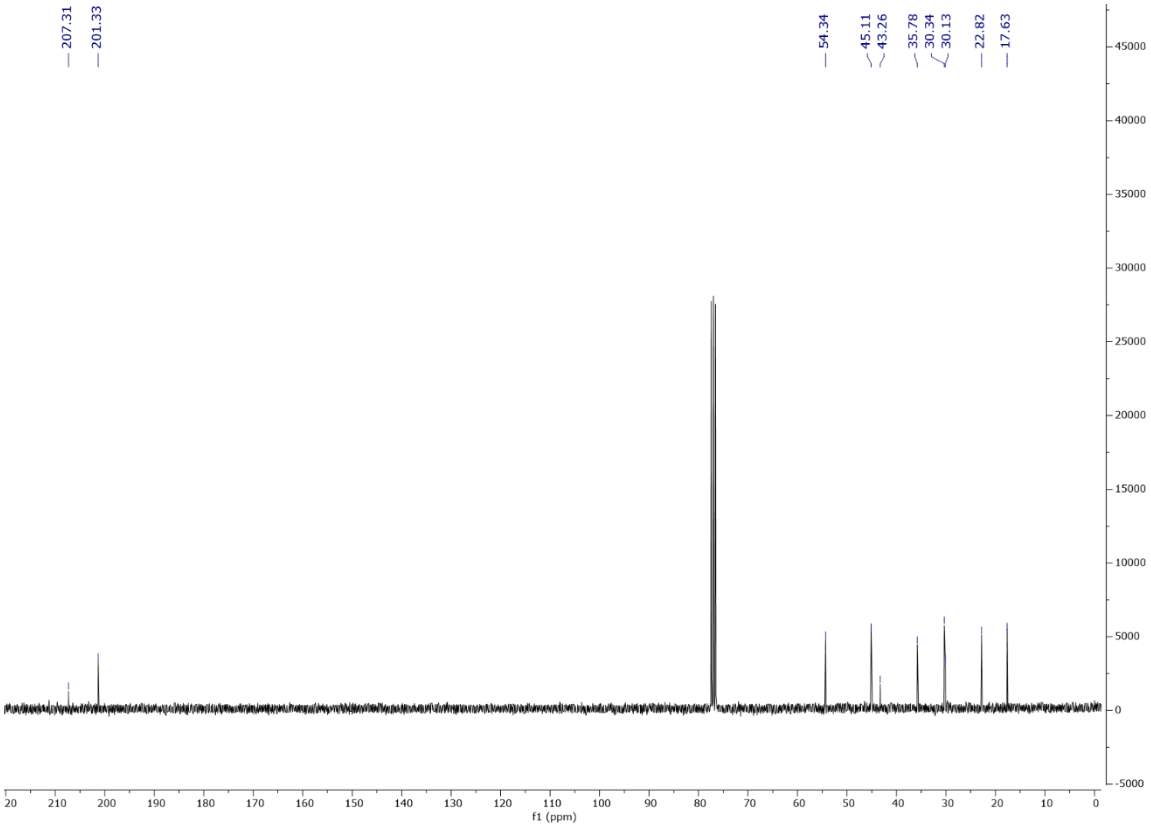

11. Xray single crystal analysis

Single crystal structure analysis of 5 (13712)

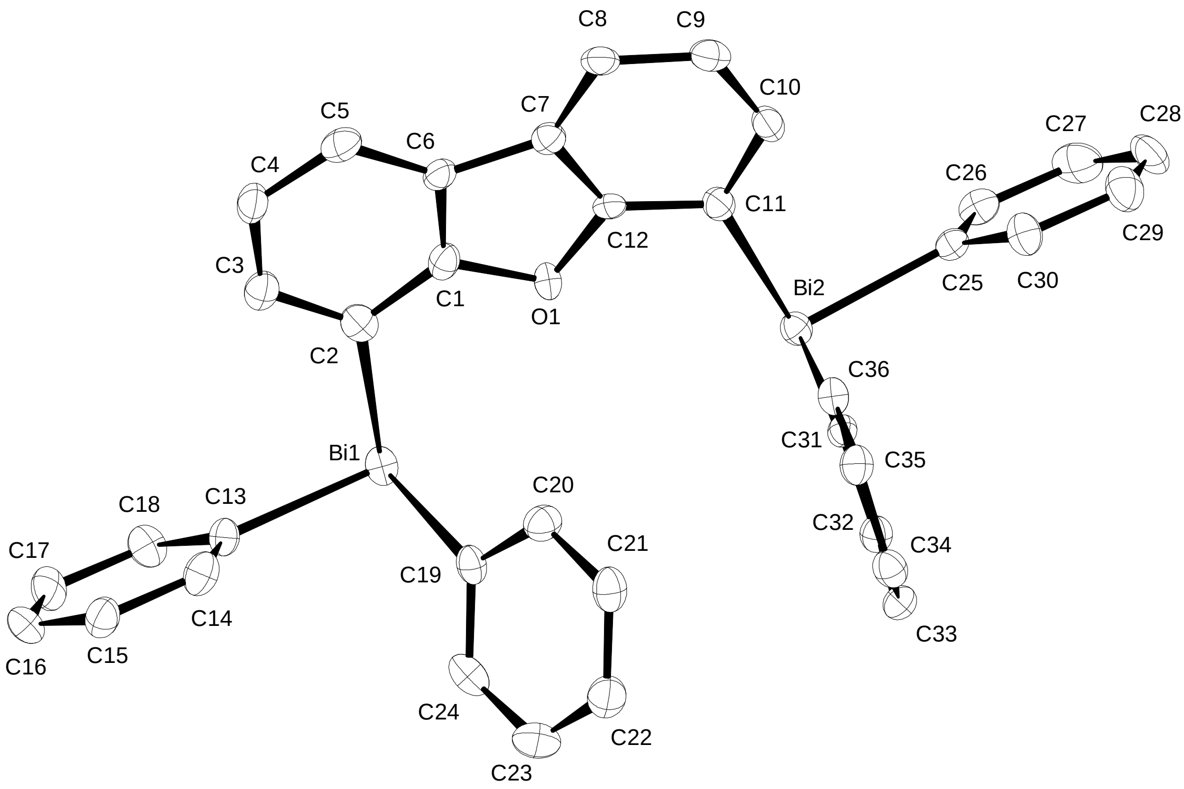

Figure 1. The molecular structure of complex **5**. H atoms have been removed for clarity.

X-ray Crystal Structure Analysis of complex 5: $C_{36}H_{26}Bi_2O$, M_r = 892.53 g mol^{-1}, colourless plate, crystal size 0.16 x 0.05 x 0.02 mm^3, orthorhombic, $P2_12_12_1$ [19], a = 6.1284(3) Å, b = 13.2853(8) Å, c = 34.731(3) Å, V = 2827.7(3) Å^3, T = 100(2) K, Z = 4, D_{calc} = 2.096 g·cm^3, λ = 0.71073 Å, $\mu(Mo\text{-}K_\alpha)$ = 12.457 mm^{-1}, Gaussian absorption correction (T_{min} = 0.17161, T_{max} = 0.77837), Bruker AXS Enraf-Nonius KappaCCD diffractometer with a FR591 rotating Mo-anode X-ray source, 2.802 < θ < 30.508°, 38138 measured reflections, 8618 independent reflections, 7693 reflections with $I > 2\sigma(I)$, R_{int} = 0.0522. The structure was solved by *SHELXS* and refined by full-matrix least-squares (*SHELXL*) against F^2 to R_1 = 0.0287 [$I > 2\sigma(I)$], wR_2 = 0.0604, 352 parameters. Absolute structure parameter Flack (x) = -0.044(6)

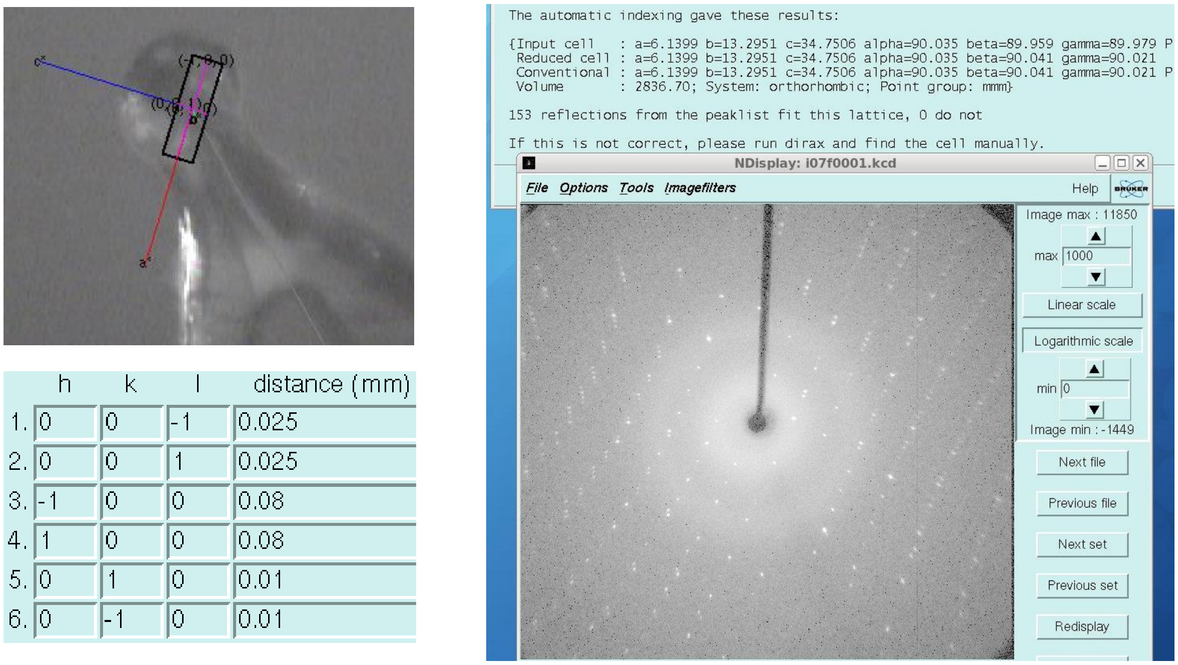

Figure 2. Crystal faces and unit cell determination of complex **5**.

INTENSITY STATISTICS FOR DATASET

Resolution	#Data	#Theory	%Complete	Redundancy	Mean I	Mean I/s	Rmerge	Rsigma
Inf - 2.60	174	182	95.6	5.57	133.10	41.53	0.0417	0.0194
2.60 - 1.73	420	420	100.0	6.09	106.45	41.07	0.0370	0.0200
1.73 - 1.37	587	587	100.0	6.00	68.90	34.90	0.0386	0.0220
1.37 - 1.19	584	584	100.0	5.73	51.66	31.40	0.0430	0.0249
1.19 - 1.08	610	610	100.0	5.29	42.94	26.93	0.0453	0.0286
1.08 - 1.00	599	599	100.0	4.91	35.62	22.89	0.0499	0.0332
1.00 - 0.95	502	502	100.0	4.69	29.04	20.79	0.0540	0.0382
0.95 - 0.90	604	604	100.0	4.41	28.21	19.38	0.0561	0.0419
0.90 - 0.86	587	587	100.0	4.14	19.86	15.36	0.0672	0.0529
0.86 - 0.83	553	553	100.0	4.08	20.22	15.00	0.0737	0.0550
0.83 - 0.80	595	595	100.0	3.82	15.55	12.04	0.0853	0.0690
0.80 - 0.77	672	672	100.0	3.65	15.49	11.15	0.0899	0.0736
0.77 - 0.75	564	564	100.0	3.56	12.98	9.92	0.1066	0.0866
0.75 - 0.73	589	589	100.0	3.37	12.18	8.91	0.1102	0.0960
0.73 - 0.71	671	673	99.7	3.21	10.65	7.79	0.1377	0.1149
0.71 - 0.69	715	718	99.6	3.09	9.17	6.50	0.1570	0.1393
0.69 - 0.68	398	400	99.5	3.02	8.15	5.77	0.1671	0.1620
0.68 - 0.66	901	905	99.6	2.96	7.78	5.24	0.1880	0.1790
0.66 - 0.65	489	494	99.0	2.84	6.96	4.47	0.2065	0.2190
0.65 - 0.64	516	522	98.9	2.70	5.69	3.45	0.2487	0.2950
0.64 - 0.63	262	276	94.9	2.55	5.92	3.29	0.2413	0.3034
0.73 - 0.63	3952	3988	99.1	2.95	8.06	5.50	0.1763	0.1757
Inf - 0.63	11592	11636	99.6	4.02	26.44	15.54	0.0575	0.0498

A resolution cut off (SHEL 99 0.7) was applied to suppress poorly measured intensities at higher diffraction angles. Complete .cif-data of the compound are available under the CCDC number **CCDC-2063973**.

Table 1. Crystal data and structure refinement.

Identification code	13712	
Empirical formula	$C_{36} H_{26} Bi_2 O$	
Color	colourless	
Formula weight	892.53 g · mol^{-1}	
Temperature	100(2) K	
Wavelength	0.71073 Å	
Crystal system	ORTHORHOMBIC	
Space group	$P2_12_12_1$, (No. 19)	
Unit cell dimensions	a = 6.1284(3) Å	α= 90°.
	b = 13.2853(8) Å	β= 90°.
	c = 34.731(3) Å	γ = 90°.
Volume	2827.7(3) Å^3	
Z	4	
Density (calculated)	2.096 Mg · m^{-3}	
Absorption coefficient	12.457 mm^{-1}	
F(000)	1664 e	
Crystal size	0.16 x 0.05 x 0.02 mm^3	
θ range for data collection	2.802 to 30.508°.	
Index ranges	-8 ≤ h ≤ 8, -18≤ k ≤ 18, -45 ≤ l ≤ 49	
Reflections collected	38138	
Independent reflections	8618 [R_{int} = 0.0522]	
Reflections with I>2σ(I)	7693	
Completeness to θ = 25.242°	99.8 %	
Absorption correction	Gaussian	
Max. and min. transmission	0.78 and 0.17	
Refinement method	Full-matrix least-squares on F^2	
Data / restraints / parameters	8618 / 0 / 352	
Goodness-of-fit on F^2	1.069	
Final R indices [I>2σ(I)]	R_1 = 0.0287	wR^2 = 0.0571
R indices (all data)	R_1 = 0.0374	wR^2 = 0.0604
Absolute structure parameter	-0.044(6)	
Largest diff. peak and hole	1.2 and -1.9 e · Å^{-3}	

Table 2. Bond lengths [Å] and angles [°].

Bi(1)-C(2)	2.262(7)	Bi(1)-C(13)	2.244(6)
Bi(1)-C(19)	2.253(7)	Bi(2)-C(11)	2.252(7)
Bi(2)-C(25)	2.243(7)	Bi(2)-C(31)	2.248(7)
O(1)-C(1)	1.397(8)	O(1)-C(12)	1.387(8)
C(1)-C(2)	1.374(9)	C(1)-C(6)	1.392(10)
C(2)-C(3)	1.387(10)	C(3)-C(4)	1.403(10)
C(4)-C(5)	1.397(10)	C(5)-C(6)	1.394(10)
C(6)-C(7)	1.456(9)	C(7)-C(8)	1.403(9)
C(7)-C(12)	1.392(9)	C(8)-C(9)	1.392(10)
C(9)-C(10)	1.385(10)	C(10)-C(11)	1.410(9)
C(11)-C(12)	1.383(9)	C(13)-C(14)	1.397(9)
C(13)-C(18)	1.389(9)	C(14)-C(15)	1.393(10)
C(15)-C(16)	1.397(10)	C(16)-C(17)	1.391(11)
C(17)-C(18)	1.381(10)	C(19)-C(20)	1.392(9)
C(19)-C(24)	1.396(11)	C(20)-C(21)	1.392(9)
C(21)-C(22)	1.365(10)	C(22)-C(23)	1.383(12)
C(23)-C(24)	1.393(12)	C(25)-C(26)	1.400(9)
C(25)-C(30)	1.378(9)	C(26)-C(27)	1.379(10)
C(27)-C(28)	1.369(12)	C(28)-C(29)	1.384(11)
C(29)-C(30)	1.405(10)	C(31)-C(32)	1.391(10)
C(31)-C(36)	1.393(9)	C(32)-C(33)	1.397(10)
C(33)-C(34)	1.382(9)	C(34)-C(35)	1.393(9)
C(35)-C(36)	1.394(10)		
C(13)-Bi(1)-C(2)	93.7(2)	C(13)-Bi(1)-C(19)	93.8(2)
C(19)-Bi(1)-C(2)	96.3(3)	C(25)-Bi(2)-C(11)	95.8(2)
C(25)-Bi(2)-C(31)	96.4(2)	C(31)-Bi(2)-C(11)	94.0(2)
C(12)-O(1)-C(1)	105.4(5)	C(2)-C(1)-O(1)	123.2(6)
C(2)-C(1)-C(6)	125.6(6)	C(6)-C(1)-O(1)	111.2(6)
C(1)-C(2)-Bi(1)	121.0(5)	C(1)-C(2)-C(3)	115.4(7)
C(3)-C(2)-Bi(1)	123.6(5)	C(2)-C(3)-C(4)	121.4(7)
C(5)-C(4)-C(3)	121.3(6)	C(6)-C(5)-C(4)	118.1(7)
C(1)-C(6)-C(5)	118.1(7)	C(1)-C(6)-C(7)	106.0(6)
C(5)-C(6)-C(7)	135.9(7)	C(8)-C(7)-C(6)	135.8(7)

C(12)-C(7)-C(6)	105.7(6)	C(12)-C(7)-C(8)	118.4(6)
C(9)-C(8)-C(7)	117.5(7)	C(10)-C(9)-C(8)	122.3(7)
C(9)-C(10)-C(11)	121.6(7)	C(10)-C(11)-Bi(2)	127.9(5)
C(12)-C(11)-Bi(2)	117.6(5)	C(12)-C(11)-C(10)	114.4(7)
O(1)-C(12)-C(7)	111.7(6)	C(11)-C(12)-O(1)	122.6(6)
C(11)-C(12)-C(7)	125.7(6)	C(14)-C(13)-Bi(1)	122.5(5)
C(18)-C(13)-Bi(1)	118.8(5)	C(18)-C(13)-C(14)	118.7(6)
C(15)-C(14)-C(13)	120.5(6)	C(14)-C(15)-C(16)	119.8(7)
C(17)-C(16)-C(15)	119.8(7)	C(18)-C(17)-C(16)	119.9(7)
C(17)-C(18)-C(13)	121.3(7)	C(20)-C(19)-Bi(1)	124.0(5)
C(20)-C(19)-C(24)	118.0(7)	C(24)-C(19)-Bi(1)	117.1(5)
C(19)-C(20)-C(21)	120.8(7)	C(22)-C(21)-C(20)	120.6(7)
C(21)-C(22)-C(23)	119.8(7)	C(22)-C(23)-C(24)	120.1(8)
C(23)-C(24)-C(19)	120.7(7)	C(26)-C(25)-Bi(2)	116.6(5)
C(30)-C(25)-Bi(2)	124.2(5)	C(30)-C(25)-C(26)	119.0(7)
C(27)-C(26)-C(25)	120.4(7)	C(28)-C(27)-C(26)	120.4(7)
C(27)-C(28)-C(29)	120.4(7)	C(28)-C(29)-C(30)	119.5(7)
C(25)-C(30)-C(29)	120.3(7)	C(32)-C(31)-Bi(2)	118.4(5)
C(32)-C(31)-C(36)	119.2(6)	C(36)-C(31)-Bi(2)	122.3(5)
C(31)-C(32)-C(33)	120.2(6)	C(34)-C(33)-C(32)	120.7(7)
C(33)-C(34)-C(35)	119.1(6)	C(34)-C(35)-C(36)	120.5(6)
C(31)-C(36)-C(35)	120.2(7)		

Single crystal structure analysis of 6 (13015)

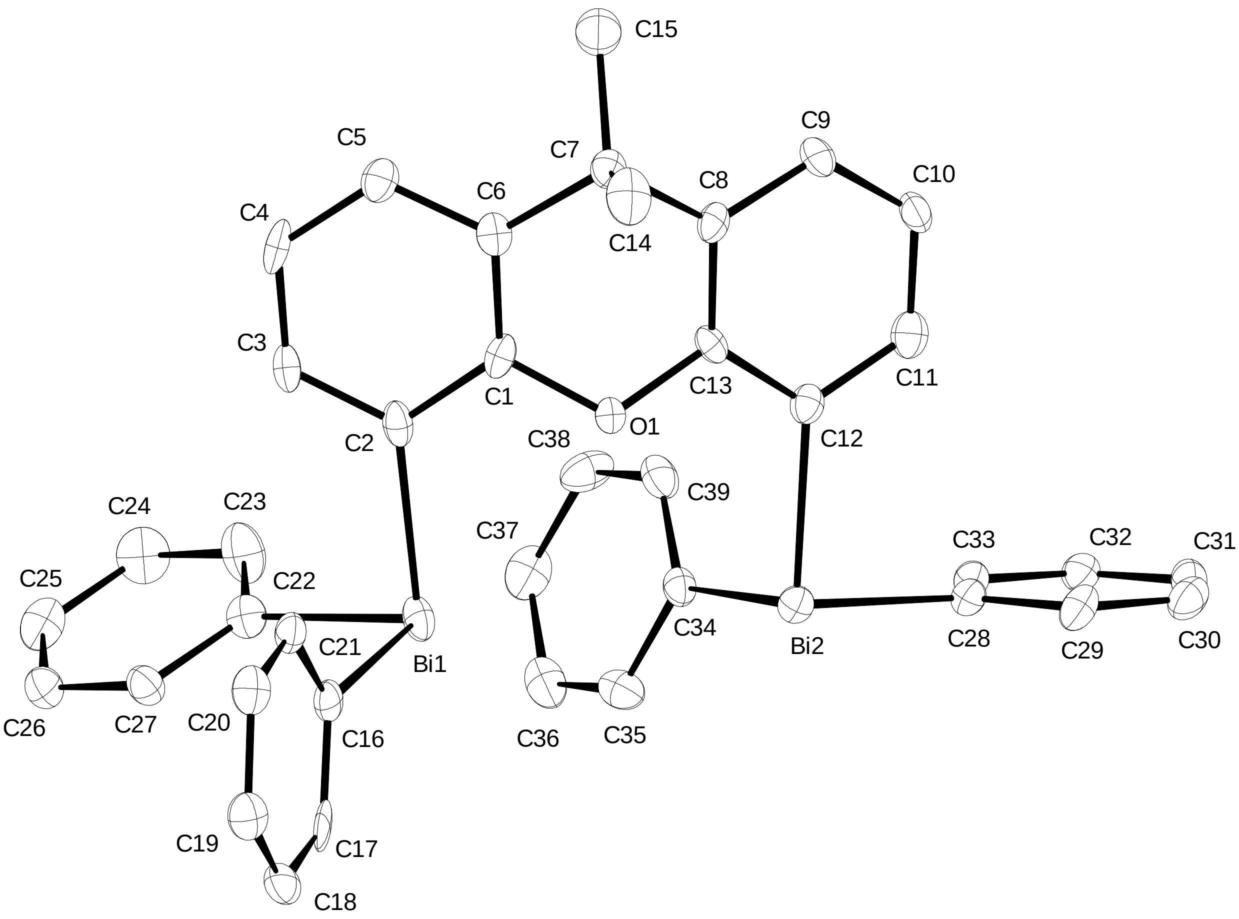

Figure 3. The molecular structure of complex **6**. H atoms have been removed for clarity.

X-ray Crystal Structure Analysis of complex 6: $C_{39}H_{32}Bi_2O$, M_r = 934.60 g mol^{-1}, colourless prism, crystal size 0.035 x 0.031 x 0.021 mm^3, orthorhombic, *F*dd2 [43], a = 37.8761(11) Å, b = 51.1850(17) Å, c = 6.6097(2) Å, V = 12814.1(7) Å^3, T = 100(2) K, Z = 16, D_{calc} = 1.938 g·cm^3, λ = 0.71073 Å, $\mu(Mo\text{-}K_\alpha)$ = 11.000 mm^{-1}, Gaussian absorption correction ($T_{\min}$ = 0.72004, $T_{\max}$ = 0.85446), Bruker-AXS Mach3 diffractometer with APEX-II detector and IμS microfocus Mo-anode X-ray source, 1.338 < θ < 32.028°, 112958 measured reflections, 11150 independent reflections, 9565 reflections with $I > 2\sigma(I)$, R_{int} = 0.0665. The structure was solved by *SHELXT* and refined by full-matrix least-squares (*SHELXL*) against F^2 to R_1 = 0.0288 [$I > 2\sigma(I)$], wR_2 = 0.0410, 381 parameters. Absolute structure parameter Flack (x) = -0.013(4)

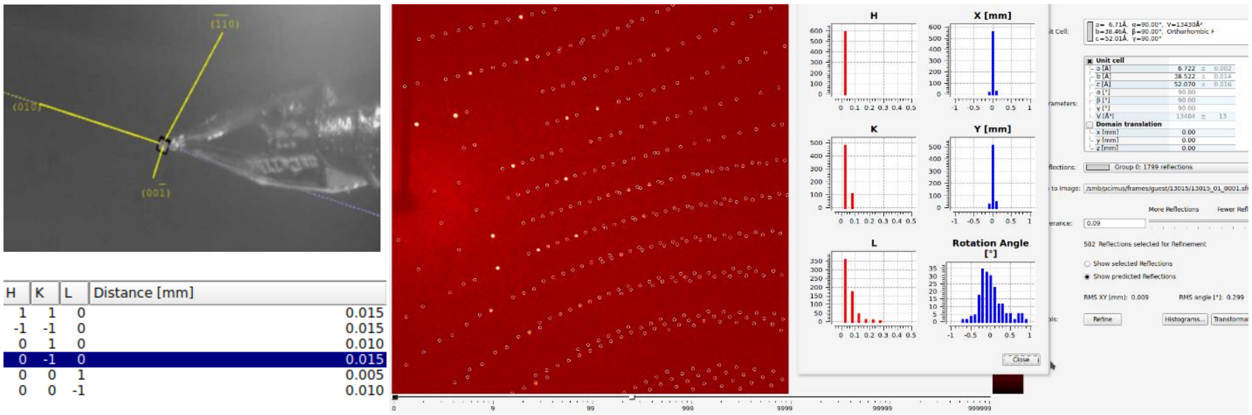

Figure 4. Crystal faces and unit cell determination of complex **6**.

INTENSITY STATISTICS FOR DATASET

Resolution	#Data	#Theory	%Complete	Redundancy	Mean I	Mean I/s	Rmerge	Rsigma
Inf - 2.66	200	201	99.5	16.96	95.19	68.76	0.0220	0.0116
2.66 - 1.75	463	463	100.0	18.49	70.61	65.58	0.0274	0.0125
1.75 - 1.38	671	671	100.0	18.31	45.20	52.21	0.0388	0.0156
1.38 - 1.20	695	695	100.0	18.26	37.69	45.16	0.0481	0.0180
1.20 - 1.09	645	645	100.0	16.62	27.11	34.02	0.0651	0.0239
1.09 - 1.01	703	703	100.0	12.42	25.28	25.97	0.0736	0.0309
1.01 - 0.95	656	656	100.0	10.20	21.96	21.40	0.0885	0.0399
0.95 - 0.90	714	714	100.0	8.84	17.50	16.32	0.1038	0.0517
0.90 - 0.86	682	682	100.0	7.95	14.48	13.41	0.1218	0.0650
0.86 - 0.83	592	592	100.0	7.70	12.60	11.82	0.1369	0.0759
0.83 - 0.80	703	703	100.0	7.15	12.03	10.28	0.1521	0.0841
0.80 - 0.78	541	541	100.0	7.03	10.33	8.97	0.1652	0.0981
0.78 - 0.75	897	897	100.0	6.82	9.33	8.03	0.1877	0.1123
0.75 - 0.73	664	664	100.0	6.61	7.82	6.62	0.2204	0.1371
0.73 - 0.71	783	784	99.9	6.26	7.07	5.81	0.2493	0.1573
0.71 - 0.70	438	438	100.0	6.16	6.44	5.20	0.2553	0.1765
0.70 - 0.68	875	876	99.9	6.04	5.64	4.60	0.2969	0.2071
0.68 - 0.67	476	479	99.4	5.84	5.22	4.11	0.3323	0.2332
0.67 - 0.66	484	492	98.4	5.71	4.70	3.69	0.3623	0.2616
0.66 - 0.64	1164	1194	97.5	4.90	3.96	2.87	0.3944	0.3500
0.64 - 0.63	144	456	31.6	0.57	2.74	1.14	0.4023	1.0221
0.73 - 0.63	4364	4719	92.5	5.22	5.28	4.15	0.3079	0.2421
Inf - 0.63	13190	13546	97.4	9.10	18.07	17.77	0.0694	0.0565

Complete .cif-data of the compound are available under the CCDC number **CCDC-2063975**.

Table 3. Crystal data and structure refinement.

Identification code	13015	
Empirical formula	$C_{39} H_{32} Bi_2 O$	
Color	colourless	
Formula weight	934.60 g · mol^{-1}	
Temperature	100(2) K	
Wavelength	0.71073 Å	
Crystal system	ORTHORHOMBIC	
Space group	*F*dd2, (No. 43)	
Unit cell dimensions	a = 37.8761(11) Å	α= 90°.
	b = 51.1850(17) Å	β= 90°.
	c = 6.6097(2) Å	γ = 90°.
Volume	12814.1(7) Å^3	
Z	16	
Density (calculated)	1.938 Mg · m^{-3}	
Absorption coefficient	11.000 mm^{-1}	
F(000)	7040 e	
Crystal size	0.035 x 0.031 x 0.021 mm^3	
θ range for data collection	1.338 to 32.028°.	
Index ranges	$-56 \le h \le 56$, $-76 \le k \le 76$, $-9 \le l \le 9$	
Reflections collected	112958	
Independent reflections	11150 [R_{int} = 0.0665]	
Reflections with I>2σ(I)	9565	
Completeness to θ = 25.242°	100.0 %	
Absorption correction	Gaussian	
Max. and min. transmission	0.85 and 0.72	
Refinement method	Full-matrix least-squares on F^2	
Data / restraints / parameters	11150 / 1 / 381	
Goodness-of-fit on F^2	1.046	
Final R indices [I>2σ(I)]	R_1 = 0.0288	wR^2 = 0.0415
R indices (all data)	R_1 = 0.0410	wR^2 = 0.0437
Absolute structure parameter	-0.013(4)	
Largest diff. peak and hole	0.9 and -1.3 e · Å^{-3}	

Table 4. Bond lengths [Å] and angles [°].

Bi(1)-C(2)	2.268(6)	Bi(1)-C(16)	2.246(5)
Bi(1)-C(22)	2.262(5)	Bi(2)-C(12)	2.255(5)
Bi(2)-C(28)	2.242(5)	Bi(2)-C(34)	2.252(5)
O(1)-C(1)	1.387(6)	O(1)-C(13)	1.391(6)
C(1)-C(2)	1.399(7)	C(1)-C(6)	1.400(7)
C(2)-C(3)	1.369(8)	C(3)-C(4)	1.391(8)
C(4)-C(5)	1.391(8)	C(5)-C(6)	1.396(8)
C(6)-C(7)	1.522(7)	C(7)-C(8)	1.523(7)
C(7)-C(14)	1.541(8)	C(7)-C(15)	1.530(8)
C(8)-C(9)	1.403(7)	C(8)-C(13)	1.390(7)
C(9)-C(10)	1.392(8)	C(10)-C(11)	1.389(8)
C(11)-C(12)	1.400(7)	C(12)-C(13)	1.394(7)
C(16)-C(17)	1.383(8)	C(16)-C(21)	1.392(7)
C(17)-C(18)	1.399(8)	C(18)-C(19)	1.375(7)
C(19)-C(20)	1.392(8)	C(20)-C(21)	1.391(7)
C(22)-C(23)	1.394(8)	C(22)-C(27)	1.395(7)
C(23)-C(24)	1.379(8)	C(24)-C(25)	1.394(8)
C(25)-C(26)	1.381(8)	C(26)-C(27)	1.391(8)
C(28)-C(29)	1.399(8)	C(28)-C(33)	1.400(7)
C(29)-C(30)	1.383(8)	C(30)-C(31)	1.395(8)
C(31)-C(32)	1.382(8)	C(32)-C(33)	1.388(7)
C(34)-C(35)	1.396(7)	C(34)-C(39)	1.392(8)
C(35)-C(36)	1.387(8)	C(36)-C(37)	1.387(9)
C(37)-C(38)	1.388(8)	C(38)-C(39)	1.370(8)
C(16)-Bi(1)-C(2)	96.16(19)	C(16)-Bi(1)-C(22)	93.82(18)
C(22)-Bi(1)-C(2)	93.60(19)	C(28)-Bi(2)-C(12)	90.36(19)
C(28)-Bi(2)-C(34)	93.06(19)	C(34)-Bi(2)-C(12)	94.8(2)
C(1)-O(1)-C(13)	116.5(4)	O(1)-C(1)-C(2)	116.8(5)
O(1)-C(1)-C(6)	120.4(5)	C(2)-C(1)-C(6)	122.8(5)
C(1)-C(2)-Bi(1)	119.9(4)	C(3)-C(2)-Bi(1)	121.5(4)
C(3)-C(2)-C(1)	118.6(5)	C(2)-C(3)-C(4)	120.5(5)
C(3)-C(4)-C(5)	120.3(5)	C(4)-C(5)-C(6)	120.9(5)
C(1)-C(6)-C(7)	119.7(5)	C(5)-C(6)-C(1)	116.9(5)

C(5)-C(6)-C(7)	123.4(5)	C(6)-C(7)-C(8)	107.4(4)
C(6)-C(7)-C(14)	108.0(4)	C(6)-C(7)-C(15)	112.0(5)
C(8)-C(7)-C(14)	108.0(5)	C(8)-C(7)-C(15)	112.0(5)
C(15)-C(7)-C(14)	109.2(4)	C(9)-C(8)-C(7)	123.3(5)
C(13)-C(8)-C(7)	119.7(5)	C(13)-C(8)-C(9)	116.9(5)
C(10)-C(9)-C(8)	121.3(5)	C(11)-C(10)-C(9)	119.5(5)
C(10)-C(11)-C(12)	121.6(5)	C(11)-C(12)-Bi(2)	123.2(4)
C(13)-C(12)-Bi(2)	120.0(4)	C(13)-C(12)-C(11)	116.7(5)
O(1)-C(13)-C(12)	115.2(4)	C(8)-C(13)-O(1)	120.7(4)
C(8)-C(13)-C(12)	124.1(5)	C(17)-C(16)-Bi(1)	117.7(4)
C(17)-C(16)-C(21)	119.0(5)	C(21)-C(16)-Bi(1)	123.0(4)
C(16)-C(17)-C(18)	121.1(5)	C(19)-C(18)-C(17)	119.7(6)
C(18)-C(19)-C(20)	119.8(5)	C(21)-C(20)-C(19)	120.4(5)
C(20)-C(21)-C(16)	120.1(5)	C(23)-C(22)-Bi(1)	119.6(4)
C(23)-C(22)-C(27)	118.3(5)	C(27)-C(22)-Bi(1)	121.9(4)
C(24)-C(23)-C(22)	121.6(6)	C(23)-C(24)-C(25)	119.7(6)
C(26)-C(25)-C(24)	119.4(5)	C(25)-C(26)-C(27)	120.8(5)
C(26)-C(27)-C(22)	120.2(5)	C(29)-C(28)-Bi(2)	119.0(4)
C(29)-C(28)-C(33)	118.0(5)	C(33)-C(28)-Bi(2)	122.9(4)
C(30)-C(29)-C(28)	121.3(5)	C(29)-C(30)-C(31)	119.7(5)
C(32)-C(31)-C(30)	119.8(5)	C(31)-C(32)-C(33)	120.4(5)
C(32)-C(33)-C(28)	120.7(5)	C(35)-C(34)-Bi(2)	118.7(4)
C(39)-C(34)-Bi(2)	122.9(4)	C(39)-C(34)-C(35)	118.4(5)
C(36)-C(35)-C(34)	120.9(6)	C(35)-C(36)-C(37)	120.1(5)
C(36)-C(37)-C(38)	118.7(5)	C(39)-C(38)-C(37)	121.5(6)
C(38)-C(39)-C(34)	120.4(5)		

Single crystal structure analysis of 7 (13364)

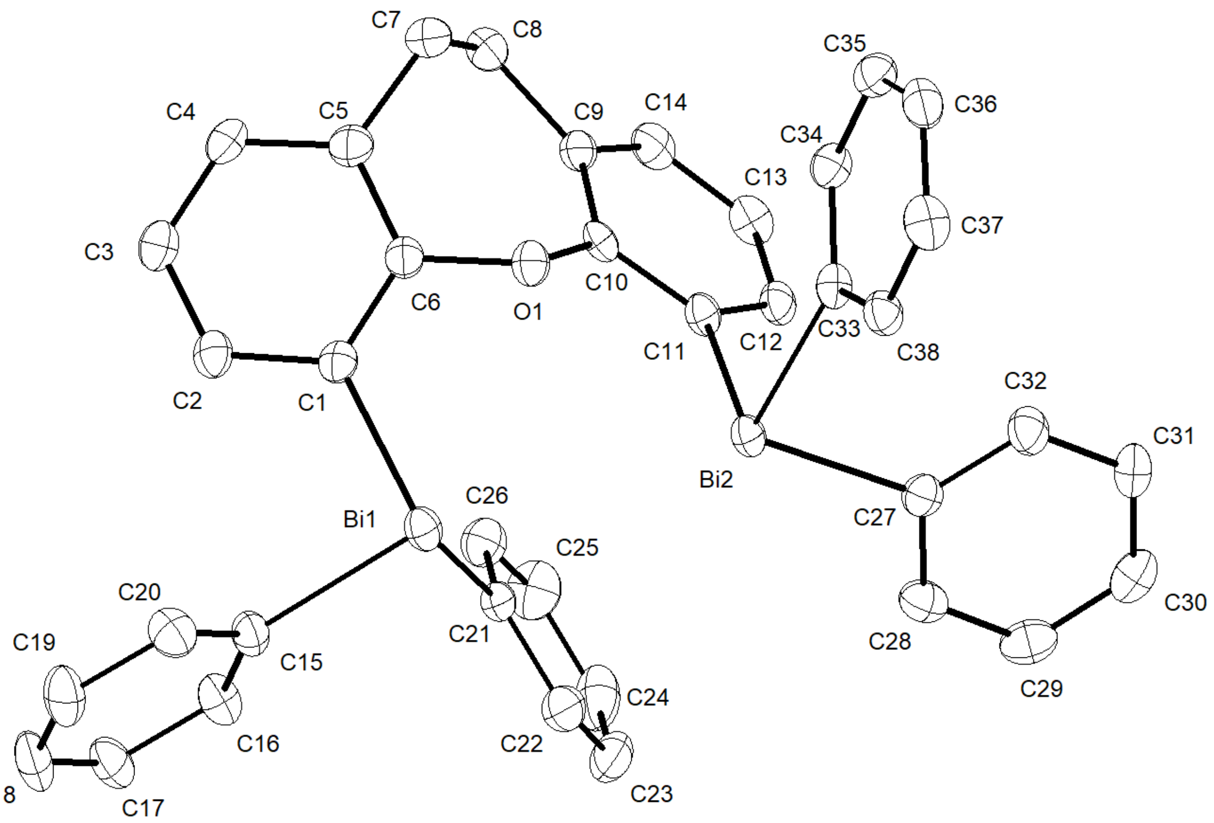

Figure 5. The molecular structure of complex **7**. H atoms have been removed for clarity.

X-ray Crystal Structure Analysis of complex 7: C_{38} H_{30} Bi_2 O, M_r = 920.58 g mol^{-1}, colourless prism, crystal size 0.07 x 0.05 x 0.04 mm^3, triclinic, *P*-1 [2], a = 10.6951(17) Å, b = 10.8286(12) Å, c = 13.7009(8) Å, α = 83.462(6) °, β = 88.592(9) °, γ = 79.178(12) °, *V* = 1548.4(3) Å^3, *T* = 100(2) K, *Z* = 2, D_{calc} = 1.975 g·cm^3, λ = 0.71073 Å, $\mu(Mo\text{-}K_{\alpha})$ = 11.378 mm^{-1}, Gaussian absorption correction ($T_{\min}$ = 0.47746, $T_{\max}$ = 0.68309), Bruker AXS Enraf-Nonius KappaCCD diffractometer with a FR591 rotating Mo-anode X-ray source, 2.768 < θ < 33.080°, 47337 measured reflections, 11728 independent reflections, 8776 reflections with $I > 2\sigma(I)$, R_{int} = 0.0502. The structure was solved by *SHELXT* and refined by full-matrix least-squares (*SHELXL*) against F^2 to R_1 = 0.0314 [$I > 2\sigma(I)$], wR_2 = 0.0553, 370 parameters.

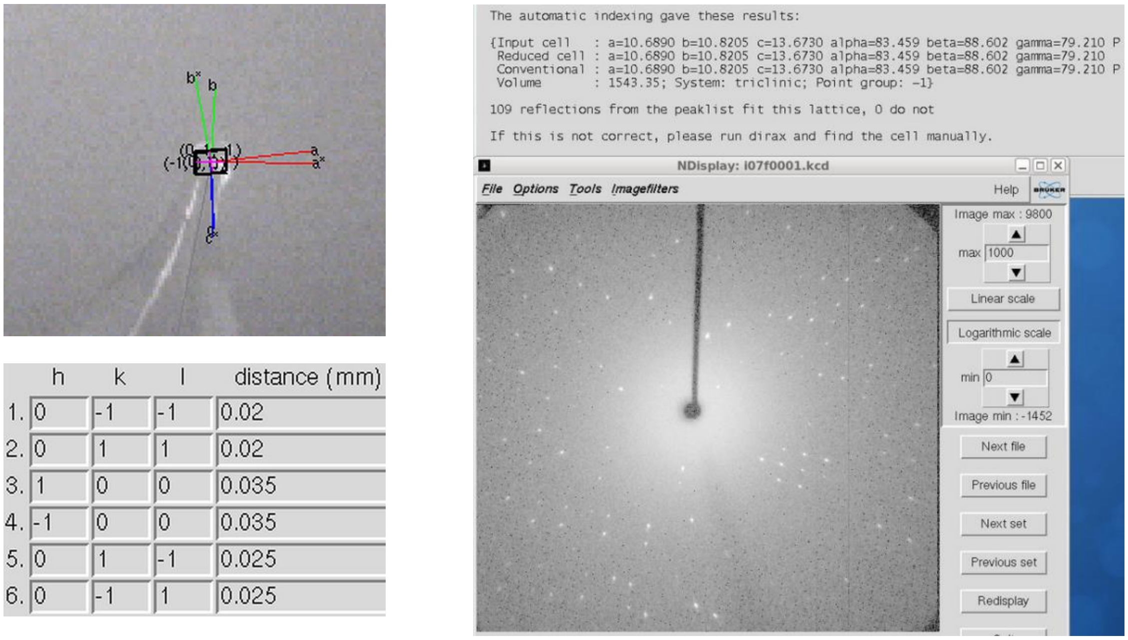

Figure 6. Crystal faces and unit cell determination of complex **7**.

INTENSITY STATISTICS FOR DATASET

Resolution	#Data	#Theory	%Complete	Redundancy	Mean I	Mean I/s	Rmerge	Rsigma
Inf - 2.60	176	184	95.7	8.72	140.68	63.91	0.0360	0.0124
2.60 - 1.75	419	419	100.0	6.29	102.07	46.48	0.0312	0.0163
1.75 - 1.40	584	584	100.0	5.68	73.63	38.69	0.0337	0.0190
1.40 - 1.22	597	597	100.0	5.35	51.08	31.55	0.0365	0.0224
1.22 - 1.11	591	591	100.0	5.04	43.27	27.54	0.0371	0.0252
1.11 - 1.03	595	595	100.0	4.85	36.74	24.02	0.0396	0.0288
1.03 - 0.97	564	564	100.0	4.55	29.10	20.28	0.0455	0.0338
0.97 - 0.92	628	628	100.0	4.44	26.32	18.54	0.0491	0.0380
0.92 - 0.88	599	599	100.0	4.23	23.14	16.89	0.0563	0.0436
0.88 - 0.84	710	710	100.0	3.94	18.12	13.57	0.0673	0.0534
0.84 - 0.82	412	412	100.0	3.81	17.62	12.66	0.0667	0.0575
0.82 - 0.79	707	707	100.0	3.72	13.90	10.81	0.0828	0.0697
0.79 - 0.77	504	504	100.0	3.48	13.18	9.90	0.0883	0.0779
0.77 - 0.75	590	590	100.0	3.42	11.46	8.66	0.1088	0.0905
0.75 - 0.73	663	663	100.0	3.26	11.17	7.89	0.1150	0.0996
0.73 - 0.71	717	717	100.0	3.10	9.60	6.91	0.1343	0.1220
0.71 - 0.70	403	403	100.0	3.08	9.82	6.74	0.1283	0.1272
0.70 - 0.68	834	834	100.0	2.94	7.09	4.87	0.1727	0.1846
0.68 - 0.67	483	483	100.0	2.84	6.68	4.24	0.2018	0.2225
0.67 - 0.66	488	488	100.0	2.78	6.32	3.84	0.2072	0.2548
0.66 - 0.65	464	482	96.3	2.60	5.41	3.03	0.2528	0.3300
0.75 - 0.65	4052	4070	99.6	2.96	8.14	5.50	0.1557	0.1669
Inf - 0.65	11728	11754	99.8	4.03	26.57	16.27	0.0489	0.0449

Complete .cif-data of the compound are available under the CCDC number **CCDC-2063978**.

Table 5. Crystal data and structure refinement.

Identification code	13364	
Empirical formula	$C_{38} H_{30} Bi_2 O$	
Color	colourless	
Formula weight	920.58 g·mol^{-1}	
Temperature	100(2) K	
Wavelength	0.71073 Å	
Crystal system	TRICLINIC	
Space group	*P*-1, (No. 2)	
Unit cell dimensions	a = 10.6951(17) Å	α= 83.462(6)°.
	b = 10.8286(12) Å	β= 88.592(9)°.
	c = 13.7009(8) Å	γ = 79.178(12)°.
Volume	1548.4(3) Å^3	
Z	2	
Density (calculated)	1.975 Mg·m^{-3}	
Absorption coefficient	11.378 mm^{-1}	
F(000)	864 e	
Crystal size	0.07 x 0.05 x 0.04 mm^3	
θ range for data collection	2.768 to 33.080°.	
Index ranges	$-16 \le h \le 16$, $-16\le k \le 16$, $-21 \le l \le 21$	
Reflections collected	47337	
Independent reflections	11728 [R_{int} = 0.0502]	
Reflections with I>2σ(I)	8776	
Completeness to θ = 25.242°	99.9 %	
Absorption correction	Gaussian	
Max. and min. transmission	0.68309 and 0.47746	
Refinement method	Full-matrix least-squares on F^2	
Data / restraints / parameters	11728 / 0 / 370	
Goodness-of-fit on F^2	1.011	
Final R indices [I>2σ(I)]	R_1 = 0.0314	wR^2 = 0.0553
R indices (all data)	R_1 = 0.0555	wR^2 = 0.0613
Extinction coefficient	n/a	
Largest diff. peak and hole	1.339 and -1.781 e·Å^{-3}	

Table 6. Bond lengths [Å] and angles [°].

Bi(2)-C(27)	2.259(4)	Bi(2)-C(33)	2.262(3)
Bi(2)-C(11)	2.252(3)	Bi(1)-C(21)	2.247(4)
Bi(1)-C(1)	2.251(3)	Bi(1)-C(15)	2.255(3)
O(1)-C(10)	1.413(4)	O(1)-C(6)	1.415(4)
C(21)-C(26)	1.393(5)	C(21)-C(22)	1.397(5)
C(27)-C(28)	1.394(5)	C(27)-C(32)	1.386(5)
C(10)-C(9)	1.405(5)	C(10)-C(11)	1.404(5)
C(30)-H(30)	0.9500	C(30)-C(29)	1.388(6)
C(30)-C(31)	1.378(6)	C(1)-C(6)	1.390(5)
C(1)-C(2)	1.403(5)	C(33)-C(34)	1.390(5)
C(33)-C(38)	1.400(5)	C(12)-H(12)	0.9500
C(12)-C(11)	1.387(5)	C(12)-C(13)	1.397(5)
C(34)-H(34)	0.9500	C(34)-C(35)	1.393(5)
C(9)-C(8)	1.523(5)	C(9)-C(14)	1.394(5)
C(28)-H(28)	0.9500	C(28)-C(29)	1.395(6)
C(13)-H(13)	0.9500	C(13)-C(14)	1.373(5)
C(26)-H(26)	0.9500	C(26)-C(25)	1.387(6)
C(6)-C(5)	1.393(5)	C(2)-H(2)	0.9500
C(2)-C(3)	1.383(5)	C(22)-H(22)	0.9500
C(22)-C(23)	1.387(6)	C(29)-H(29)	0.9500
C(15)-C(20)	1.396(5)	C(15)-C(16)	1.388(5)
C(7)-H(7A)	0.9900	C(7)-H(7B)	0.9900
C(7)-C(8)	1.529(6)	C(7)-C(5)	1.496(5)
C(31)-H(31)	0.9500	C(31)-C(32)	1.384(5)
C(20)-H(20)	0.9500	C(20)-C(19)	1.396(5)
C(19)-H(19)	0.9500	C(19)-C(18)	1.379(6)
C(32)-H(32)	0.9500	C(16)-H(16)	0.9500
C(16)-C(17)	1.392(5)	C(38)-H(38)	0.9500
C(38)-C(37)	1.385(5)	C(8)-H(8A)	0.9900
C(8)-H(8B)	0.9900	C(5)-C(4)	1.395(5)
C(35)-H(35)	0.9500	C(35)-C(36)	1.386(5)
C(14)-H(14)	0.9500	C(25)-H(25)	0.9500
C(25)-C(24)	1.392(6)	C(36)-H(36)	0.9500
C(36)-C(37)	1.381(6)	C(17)-H(17)	0.9500

C(17)-C(18)	1.396(6)	C(23)-H(23)	0.9500
C(23)-C(24)	1.383(7)	C(37)-H(37)	0.9500
C(18)-H(18)	0.9500	C(3)-H(3)	0.9500
C(3)-C(4)	1.389(5)	C(24)-H(24)	0.9500
C(4)-H(4)	0.9500		

C(27)-Bi(2)-C(33)	94.34(12)	C(11)-Bi(2)-C(27)	92.72(12)
C(11)-Bi(2)-C(33)	93.21(12)	C(21)-Bi(1)-C(1)	97.62(12)
C(21)-Bi(1)-C(15)	94.52(13)	C(1)-Bi(1)-C(15)	92.32(12)
C(10)-O(1)-C(6)	118.8(3)	C(26)-C(21)-Bi(1)	122.8(3)
C(26)-C(21)-C(22)	118.9(4)	C(22)-C(21)-Bi(1)	118.4(3)
C(28)-C(27)-Bi(2)	118.8(3)	C(32)-C(27)-Bi(2)	123.0(3)
C(32)-C(27)-C(28)	118.2(3)	C(9)-C(10)-O(1)	125.0(3)
C(11)-C(10)-O(1)	114.0(3)	C(11)-C(10)-C(9)	121.0(3)
C(29)-C(30)-H(30)	120.2	C(31)-C(30)-H(30)	120.2
C(31)-C(30)-C(29)	119.6(4)	C(6)-C(1)-Bi(1)	121.3(2)
C(6)-C(1)-C(2)	117.6(3)	C(2)-C(1)-Bi(1)	120.5(2)
C(34)-C(33)-Bi(2)	122.9(2)	C(34)-C(33)-C(38)	118.2(3)
C(38)-C(33)-Bi(2)	118.7(3)	C(11)-C(12)-H(12)	119.8
C(11)-C(12)-C(13)	120.4(3)	C(13)-C(12)-H(12)	119.8
C(33)-C(34)-H(34)	119.6	C(33)-C(34)-C(35)	120.9(3)
C(35)-C(34)-H(34)	119.6	C(10)-C(9)-C(8)	126.3(3)
C(14)-C(9)-C(10)	117.1(3)	C(14)-C(9)-C(8)	116.4(3)
C(10)-C(11)-Bi(2)	118.8(2)	C(12)-C(11)-Bi(2)	121.8(3)
C(12)-C(11)-C(10)	119.3(3)	C(27)-C(28)-H(28)	119.5
C(27)-C(28)-C(29)	121.0(4)	C(29)-C(28)-H(28)	119.5
C(12)-C(13)-H(13)	120.4	C(14)-C(13)-C(12)	119.1(3)
C(14)-C(13)-H(13)	120.4	C(21)-C(26)-H(26)	119.8
C(25)-C(26)-C(21)	120.4(4)	C(25)-C(26)-H(26)	119.8
C(1)-C(6)-O(1)	118.6(3)	C(1)-C(6)-C(5)	122.6(3)
C(5)-C(6)-O(1)	118.7(3)	C(1)-C(2)-H(2)	119.6
C(3)-C(2)-C(1)	120.7(3)	C(3)-C(2)-H(2)	119.6
C(21)-C(22)-H(22)	119.7	C(23)-C(22)-C(21)	120.6(4)
C(23)-C(22)-H(22)	119.7	C(30)-C(29)-C(28)	119.6(4)
C(30)-C(29)-H(29)	120.2	C(28)-C(29)-H(29)	120.2
C(20)-C(15)-Bi(1)	118.5(3)	C(16)-C(15)-Bi(1)	122.3(3)

C(16)-C(15)-C(20)	119.2(3)	H(7A)-C(7)-H(7B)	108.3
C(8)-C(7)-H(7A)	109.9	C(8)-C(7)-H(7B)	109.9
C(5)-C(7)-H(7A)	109.9	C(5)-C(7)-H(7B)	109.9
C(5)-C(7)-C(8)	108.8(3)	C(30)-C(31)-H(31)	119.7
C(30)-C(31)-C(32)	120.5(4)	C(32)-C(31)-H(31)	119.7
C(15)-C(20)-H(20)	119.9	C(15)-C(20)-C(19)	120.3(4)
C(19)-C(20)-H(20)	119.9	C(20)-C(19)-H(19)	119.8
C(18)-C(19)-C(20)	120.4(4)	C(18)-C(19)-H(19)	119.8
C(27)-C(32)-H(32)	119.5	C(31)-C(32)-C(27)	121.0(4)
C(31)-C(32)-H(32)	119.5	C(15)-C(16)-H(16)	119.8
C(15)-C(16)-C(17)	120.3(4)	C(17)-C(16)-H(16)	119.8
C(33)-C(38)-H(38)	119.5	C(37)-C(38)-C(33)	120.9(3)
C(37)-C(38)-H(38)	119.5	C(9)-C(8)-C(7)	115.0(3)
C(9)-C(8)-H(8A)	108.5	C(9)-C(8)-H(8B)	108.5
C(7)-C(8)-H(8A)	108.5	C(7)-C(8)-H(8B)	108.5
H(8A)-C(8)-H(8B)	107.5	C(6)-C(5)-C(7)	119.5(3)
C(6)-C(5)-C(4)	118.1(3)	C(4)-C(5)-C(7)	122.1(3)
C(34)-C(35)-H(35)	120.0	C(36)-C(35)-C(34)	120.1(4)
C(36)-C(35)-H(35)	120.0	C(9)-C(14)-H(14)	118.6
C(13)-C(14)-C(9)	122.7(3)	C(13)-C(14)-H(14)	118.6
C(26)-C(25)-H(25)	119.8	C(26)-C(25)-C(24)	120.3(4)
C(24)-C(25)-H(25)	119.8	C(35)-C(36)-H(36)	120.1
C(37)-C(36)-C(35)	119.7(3)	C(37)-C(36)-H(36)	120.1
C(16)-C(17)-H(17)	119.9	C(16)-C(17)-C(18)	120.3(4)
C(18)-C(17)-H(17)	119.9	C(22)-C(23)-H(23)	119.9
C(24)-C(23)-C(22)	120.2(4)	C(24)-C(23)-H(23)	119.9
C(38)-C(37)-H(37)	119.9	C(36)-C(37)-C(38)	120.2(3)
C(36)-C(37)-H(37)	119.9	C(19)-C(18)-C(17)	119.5(4)
C(19)-C(18)-H(18)	120.2	C(17)-C(18)-H(18)	120.2
C(2)-C(3)-H(3)	119.8	C(2)-C(3)-C(4)	120.4(3)
C(4)-C(3)-H(3)	119.8	C(25)-C(24)-H(24)	120.2
C(23)-C(24)-C(25)	119.6(4)	C(23)-C(24)-H(24)	120.2
C(5)-C(4)-H(4)	119.8	C(3)-C(4)-C(5)	120.4(3)
C(3)-C(4)-H(4)	119.8		

Single crystal structure analysis of 8 (13443)

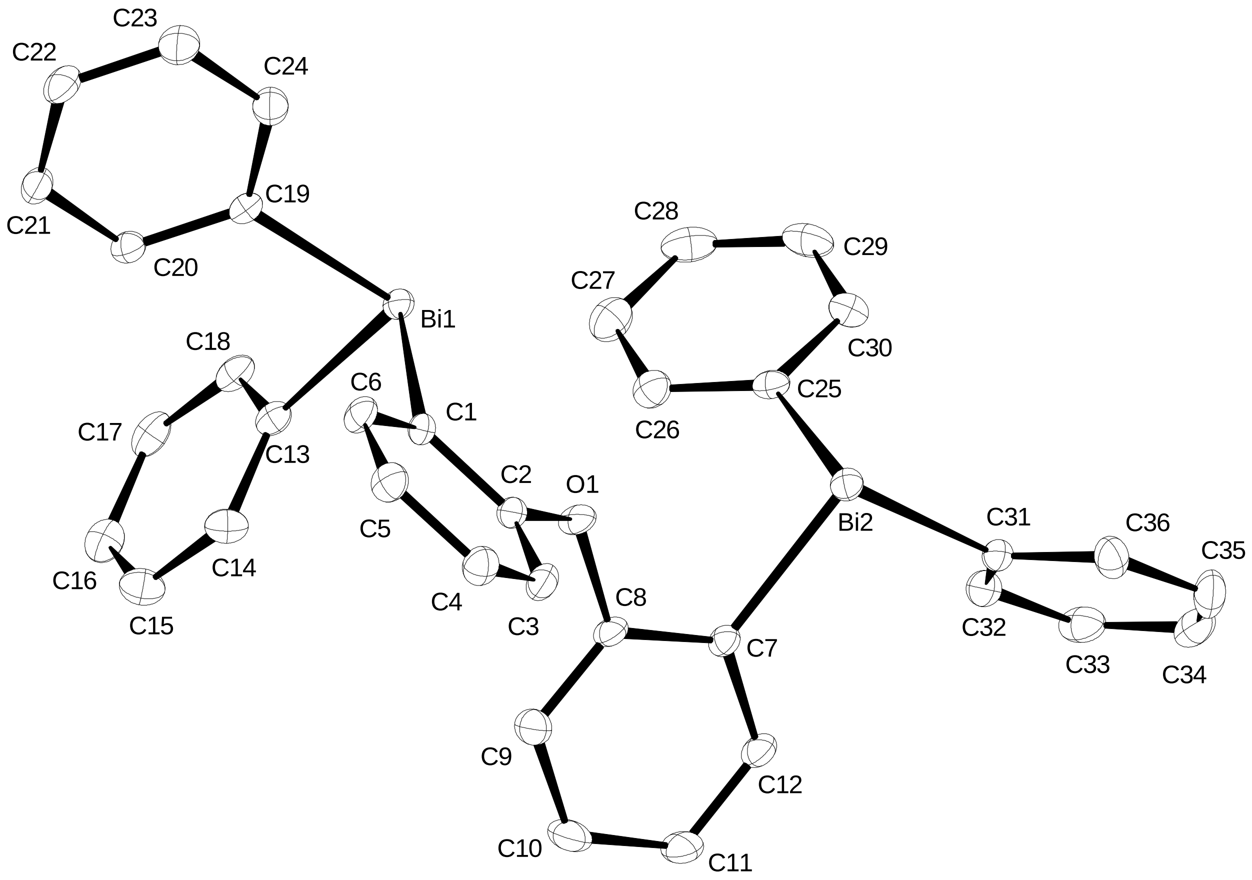

Figure 7. The molecular structure of complex **8**. H atoms have been removed for clarity.

X-ray Crystal Structure Analysis of complex 8: $C_{36}H_{28}Bi_2O$, M_r = 894.54 g mol^{-1}, colourless needle, crystal size 0.056 x 0.041 x 0.020 mm^3, monoclinic, $P2_1/c$ [14], a = 11.0777(7) Å, b = 17.8599(11) Å, c = 15.0585(9) Å, β = 92.997(2) °, V = 2975.2(3) Å^3, T = 100(2) K, Z = 4, D_{calc} = 1.997 g·cm^3, λ = 0.71073 Å, $\mu(Mo\text{-}K_\alpha)$ = 11.840 mm^{-1}, Gaussian absorption correction (T_{min} = 0.60899, T_{max} = 0.85677), Bruker-AXS Mach3 diffractometer with APEX-II detector and IµS microfocus Mo-anode X-ray source, 1.770 < θ < 34.337 °, 116047 measured reflections, 12473 independent reflections, 10883 reflections with $I > 2\sigma(I)$, R_{int} = 0.0363. The structure was solved by *SHELXT* and refined by full-matrix least-squares (*SHELXL*) against F^2 to R_1 = 0.0187 [$I > 2\sigma(I)$], wR_2 = 0.0330, 352 parameters.

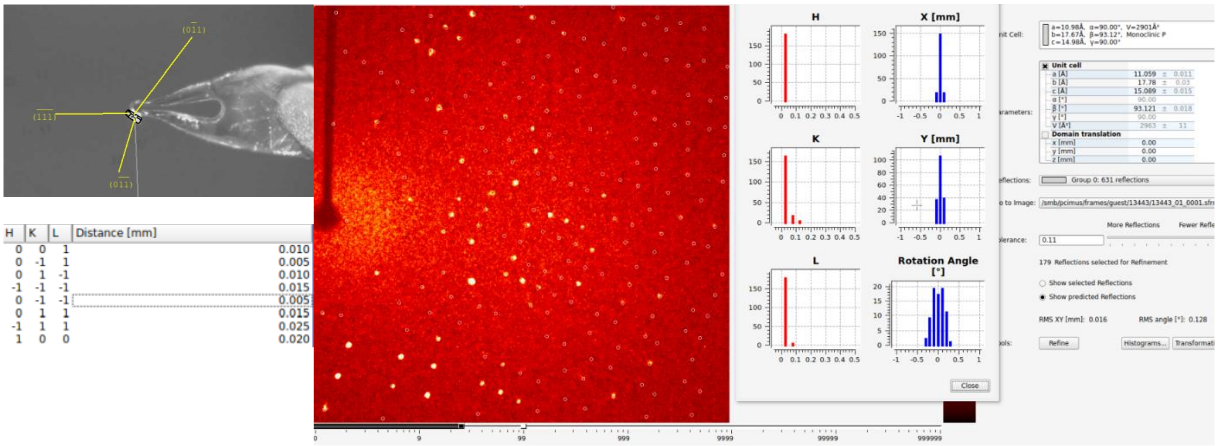

Figure 8. Crystal faces and unit cell determination of complex **8**.

INTENSITY STATISTICS FOR DATASET

Resolution	#Data	#Theory	%Complete	Redundancy	Mean I	Mean I/s	Rmerge	Rsigma
Inf - 2.62	193	193	100.0	16.66	105.31	100.95	0.0218	0.0077
2.62 - 1.73	465	465	100.0	18.07	77.19	95.92	0.0213	0.0078
1.73 - 1.36	659	659	100.0	18.21	56.68	85.70	0.0239	0.0084
1.36 - 1.19	637	637	100.0	17.89	40.21	73.92	0.0290	0.0095
1.19 - 1.08	644	644	100.0	16.19	34.66	63.67	0.0328	0.0110
1.08 - 1.00	659	659	100.0	12.04	30.16	50.59	0.0367	0.0144
1.00 - 0.94	643	643	100.0	9.96	24.96	41.52	0.0403	0.0173
0.94 - 0.89	700	700	100.0	8.42	22.48	34.84	0.0441	0.0206
0.89 - 0.85	654	654	100.0	7.81	18.60	29.75	0.0501	0.0243
0.85 - 0.82	606	606	100.0	7.49	18.04	27.34	0.0527	0.0262
0.82 - 0.79	676	676	100.0	7.22	14.37	23.26	0.0635	0.0317
0.79 - 0.77	526	526	100.0	6.93	14.23	21.71	0.0608	0.0330
0.77 - 0.74	882	882	100.0	6.73	13.25	19.76	0.0695	0.0367
0.74 - 0.72	680	680	100.0	6.25	12.03	17.70	0.0789	0.0427
0.72 - 0.71	354	354	100.0	6.39	12.06	17.95	0.0819	0.0426
0.71 - 0.69	773	773	100.0	6.05	9.64	14.48	0.0936	0.0526
0.69 - 0.67	914	914	100.0	5.83	9.13	13.50	0.1011	0.0579
0.67 - 0.66	488	488	100.0	5.68	8.11	11.88	0.1123	0.0651
0.66 - 0.65	509	509	100.0	5.49	7.47	11.07	0.1194	0.0728
0.65 - 0.64	555	555	100.0	5.38	8.04	11.27	0.1236	0.0705
0.64 - 0.63	601	601	100.0	5.13	7.03	9.96	0.1317	0.0819
0.73 - 0.63	4559	4559	100.0	5.74	8.91	13.13	0.1027	0.0599
Inf - 0.63	12818	12818	100.0	9.20	22.61	34.27	0.0359	0.0217

Complete .cif-data of the compound are available under the CCDC number **CCDC-2063976**.

Table 7. Crystal data and structure refinement.

Identification code	13443	
Empirical formula	$C_{36}H_{28}Bi_2O$	
Color	colourless	
Formula weight	894.54 g · mol^{-1}	
Temperature	100(2) K	
Wavelength	0.71073 Å	
Crystal system	MONOCLINIC	
Space group	$P2_1$/c, (No. 14)	
Unit cell dimensions	a = 11.0777(7) Å	α= 90°.
	b = 17.8599(11) Å	β= 92.997(2)°.
	c = 15.0585(9) Å	γ = 90°.
Volume	2975.2(3) Å^3	
Z	4	
Density (calculated)	1.997 Mg · m^{-3}	
Absorption coefficient	11.840 mm^{-1}	
F(000)	1672 e	
Crystal size	0.056 x 0.041 x 0.020 mm^3	
θ range for data collection	1.770 to 34.337°.	
Index ranges	-17 ≤ h ≤ 17, -28≤ k ≤ 28, -23 ≤ l ≤ 23	
Reflections collected	116047	
Independent reflections	12473 [R_{int} = 0.0363]	
Reflections with I>2σ(I)	10883	
Completeness to θ = 25.242°	100.0 %	
Absorption correction	Gaussian	
Max. and min. transmission	0.86 and 0.61	
Refinement method	Full-matrix least-squares on F^2	
Data / restraints / parameters	12473 / 0 / 352	
Goodness-of-fit on F^2	1.038	
Final R indices [I>2σ(I)]	R_1 = 0.0187	wR^2 = 0.0330
R indices (all data)	R_1 = 0.0262	wR^2 = 0.0344
Largest diff. peak and hole	1.9 and -1.2 e · Å^{-3}	

Table 8. Bond lengths [Å] and angles [°].

Bi(1)-C(1)	2.2624(17)	Bi(1)-C(13)	2.2494(19)
Bi(1)-C(19)	2.2605(18)	Bi(2)-C(7)	2.2426(18)
Bi(2)-C(25)	2.2491(18)	Bi(2)-C(31)	2.2525(19)
O(1)-C(2)	1.400(2)	O(1)-C(8)	1.397(2)
C(1)-C(2)	1.384(3)	C(1)-C(6)	1.399(2)
C(2)-C(3)	1.394(3)	C(3)-C(4)	1.393(3)
C(4)-C(5)	1.388(3)	C(5)-C(6)	1.391(3)
C(7)-C(8)	1.389(3)	C(7)-C(12)	1.400(3)
C(8)-C(9)	1.381(3)	C(9)-C(10)	1.390(3)
C(10)-C(11)	1.387(3)	C(11)-C(12)	1.388(3)
C(13)-C(14)	1.397(3)	C(13)-C(18)	1.394(2)
C(14)-C(15)	1.395(3)	C(15)-C(16)	1.391(3)
C(16)-C(17)	1.386(3)	C(17)-C(18)	1.390(3)
C(19)-C(20)	1.392(3)	C(19)-C(24)	1.390(3)
C(20)-C(21)	1.393(3)	C(21)-C(22)	1.387(3)
C(22)-C(23)	1.385(3)	C(23)-C(24)	1.395(3)
C(25)-C(26)	1.390(3)	C(25)-C(30)	1.395(3)
C(26)-C(27)	1.395(3)	C(27)-C(28)	1.386(3)
C(28)-C(29)	1.380(3)	C(29)-C(30)	1.385(3)
C(31)-C(32)	1.395(3)	C(31)-C(36)	1.395(3)
C(32)-C(33)	1.395(3)	C(33)-C(34)	1.388(3)
C(34)-C(35)	1.384(4)	C(35)-C(36)	1.394(3)
C(13)-Bi(1)-C(1)	97.61(6)	C(13)-Bi(1)-C(19)	92.20(7)
C(19)-Bi(1)-C(1)	92.13(6)	C(7)-Bi(2)-C(25)	94.66(7)
C(7)-Bi(2)-C(31)	97.14(7)	C(25)-Bi(2)-C(31)	91.50(7)
C(8)-O(1)-C(2)	118.78(14)	C(2)-C(1)-Bi(1)	118.57(12)
C(2)-C(1)-C(6)	117.86(16)	C(6)-C(1)-Bi(1)	122.94(13)
C(1)-C(2)-O(1)	117.03(16)	C(1)-C(2)-C(3)	122.08(17)
C(3)-C(2)-O(1)	120.75(16)	C(4)-C(3)-C(2)	118.99(18)
C(5)-C(4)-C(3)	120.12(18)	C(4)-C(5)-C(6)	119.83(18)
C(5)-C(6)-C(1)	121.13(18)	C(8)-C(7)-Bi(2)	117.52(13)
C(8)-C(7)-C(12)	118.09(17)	C(12)-C(7)-Bi(2)	124.06(13)
C(7)-C(8)-O(1)	116.54(16)	C(9)-C(8)-O(1)	121.33(17)

C(9)-C(8)-C(7)	122.10(17)	C(8)-C(9)-C(10)	118.84(19)
C(11)-C(10)-C(9)	120.53(19)	C(10)-C(11)-C(12)	119.85(18)
C(11)-C(12)-C(7)	120.59(18)	C(14)-C(13)-Bi(1)	124.02(13)
C(18)-C(13)-Bi(1)	116.99(14)	C(18)-C(13)-C(14)	118.83(18)
C(15)-C(14)-C(13)	120.28(17)	C(16)-C(15)-C(14)	120.39(19)
C(17)-C(16)-C(15)	119.39(19)	C(16)-C(17)-C(18)	120.45(18)
C(17)-C(18)-C(13)	120.66(18)	C(20)-C(19)-Bi(1)	122.43(13)
C(24)-C(19)-Bi(1)	118.65(14)	C(24)-C(19)-C(20)	118.92(17)
C(19)-C(20)-C(21)	120.44(17)	C(22)-C(21)-C(20)	120.38(18)
C(23)-C(22)-C(21)	119.47(18)	C(22)-C(23)-C(24)	120.22(18)
C(19)-C(24)-C(23)	120.57(19)	C(26)-C(25)-Bi(2)	123.91(14)
C(26)-C(25)-C(30)	118.84(18)	C(30)-C(25)-Bi(2)	117.20(14)
C(25)-C(26)-C(27)	120.39(19)	C(28)-C(27)-C(26)	119.7(2)
C(29)-C(28)-C(27)	120.43(19)	C(28)-C(29)-C(30)	119.6(2)
C(29)-C(30)-C(25)	120.9(2)	C(32)-C(31)-Bi(2)	123.08(14)
C(32)-C(31)-C(36)	118.90(18)	C(36)-C(31)-Bi(2)	117.84(14)
C(31)-C(32)-C(33)	120.53(19)	C(34)-C(33)-C(32)	119.8(2)
C(35)-C(34)-C(33)	120.2(2)	C(34)-C(35)-C(36)	119.9(2)
C(35)-C(36)-C(31)	120.6(2)		

Single crystal structure analysis of 9 (13680)

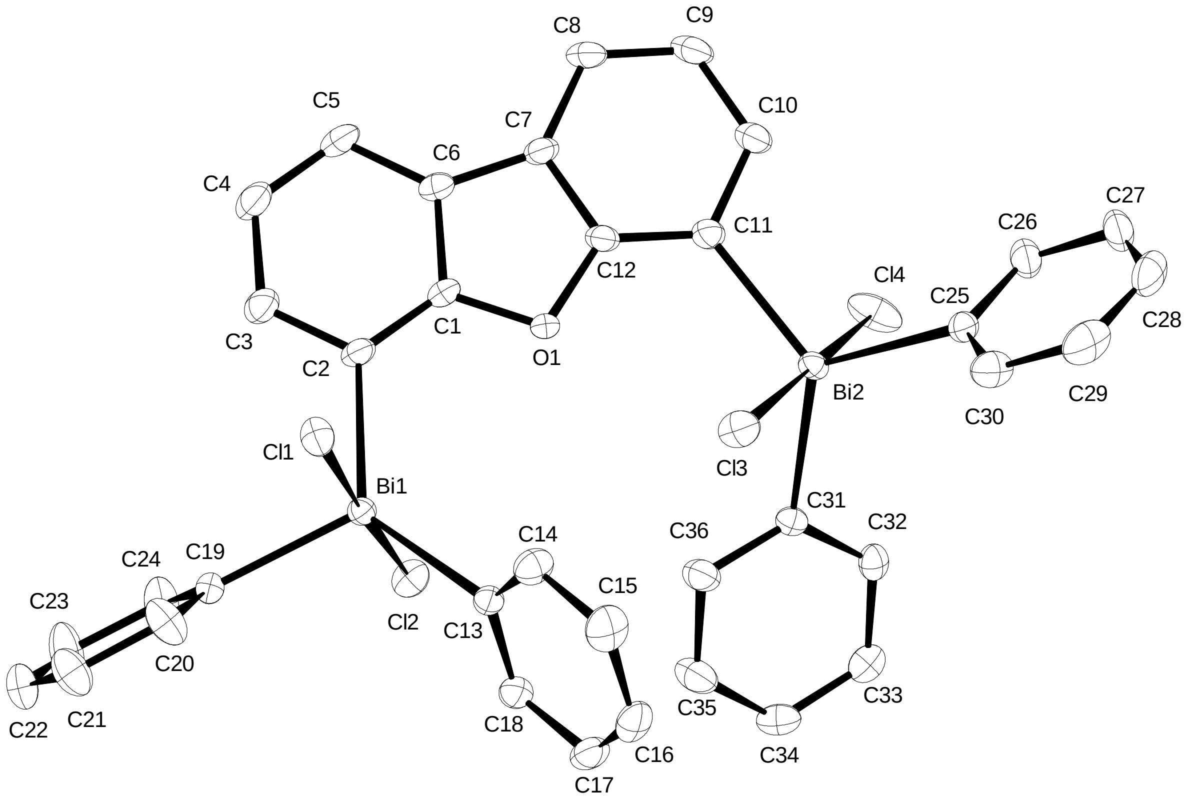

Figure 9. The molecular structure of complex **9**. H atoms have been removed for clarity.

X-ray Crystal Structure Analysis of complex 9: C_{36} H_{26} Bi_2 Cl_4 O, M_r = 1034.33 g mol^{-1}, colourless plate, crystal size 0.062 x 0.035 x 0.011 mm^3, triclinic, *P*-1 [2], a = 9.0281(4) Å, b = 12.1880(5) Å, c = 15.1195(6) Å, α = 101.289(2) °, β = 90.246(2) °, γ = 95.156(2) ° *V* = 1624.47(12) Å^3, *T* = 100(2) K, *Z* = 2, D_{calc} = 2.115 g·cm^3, λ = 0.71073 Å, $\mu(Mo\text{-}K_\alpha)$ = 11.176 mm^{-1}, Gaussian absorption correction ($T_{\min}$ = 0.62769, $T_{\max}$ = 0.89794), Bruker-AXS Mach3 diffractometer with APEX-II detector and IμS microfocus Mo-anode X-ray source, 1.374 < θ < 35.077 °, 65647 measured reflections, 14207 independent reflections, 11437 reflections with $I > 2\sigma(I)$, R_{int} = 0.0403. The structure was solved by *SHELXT* and refined by full-matrix least-squares (*SHELXL*) against F^2 to R_1 = 0.0262 [$I > 2\sigma(I)$], wR_2 = 0.0566, 388 parameters.

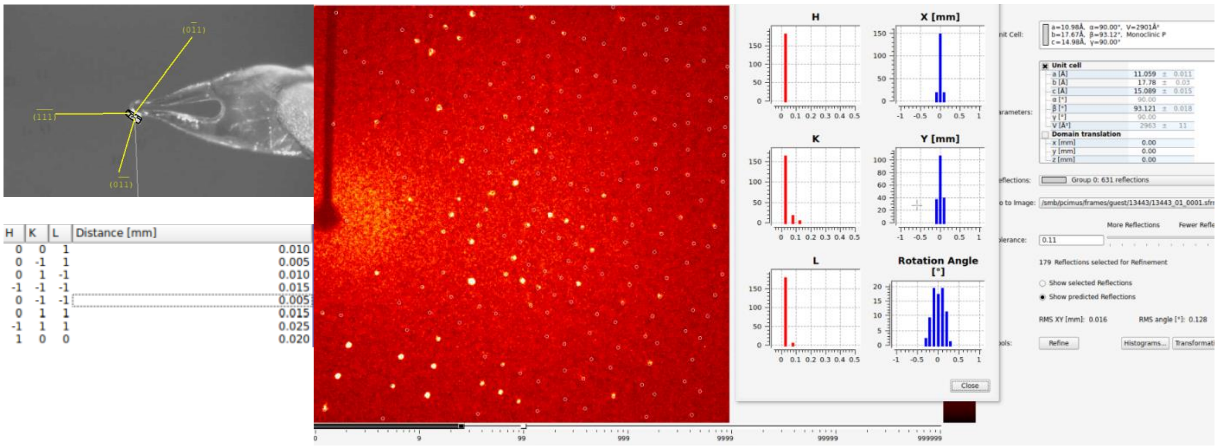

Figure 10. Crystal faces and unit cell determination of complex **9**.

INTENSITY STATISTICS FOR DATASET

Resolution	#Data	#Theory	%Complete	Redundancy	Mean I	Mean I/s	Rmerge	Rsigma
Inf - 2.62	193	193	100.0	16.66	105.31	100.95	0.0218	0.0077
2.62 - 1.73	465	465	100.0	18.07	77.19	95.92	0.0213	0.0078
1.73 - 1.36	659	659	100.0	18.21	56.68	85.70	0.0239	0.0084
1.36 - 1.19	637	637	100.0	17.89	40.21	73.92	0.0290	0.0095
1.19 - 1.08	644	644	100.0	16.19	34.66	63.67	0.0328	0.0110
1.08 - 1.00	659	659	100.0	12.04	30.16	50.59	0.0367	0.0144
1.00 - 0.94	643	643	100.0	9.96	24.96	41.52	0.0403	0.0173
0.94 - 0.89	700	700	100.0	8.42	22.48	34.84	0.0441	0.0206
0.89 - 0.85	654	654	100.0	7.81	18.60	29.75	0.0501	0.0243
0.85 - 0.82	606	606	100.0	7.49	18.04	27.34	0.0527	0.0262
0.82 - 0.79	676	676	100.0	7.22	14.37	23.26	0.0635	0.0317
0.79 - 0.77	526	526	100.0	6.93	14.23	21.71	0.0608	0.0330
0.77 - 0.74	882	882	100.0	6.73	13.25	19.76	0.0695	0.0367
0.74 - 0.72	680	680	100.0	6.25	12.03	17.70	0.0789	0.0427
0.72 - 0.71	354	354	100.0	6.39	12.06	17.95	0.0819	0.0426
0.71 - 0.69	773	773	100.0	6.05	9.64	14.48	0.0936	0.0526
0.69 - 0.67	914	914	100.0	5.83	9.13	13.50	0.1011	0.0579
0.67 - 0.66	488	488	100.0	5.68	8.11	11.88	0.1123	0.0651
0.66 - 0.65	509	509	100.0	5.49	7.47	11.07	0.1194	0.0728
0.65 - 0.64	555	555	100.0	5.38	8.04	11.27	0.1236	0.0705
0.64 - 0.63	601	601	100.0	5.13	7.03	9.96	0.1317	0.0819
0.73 - 0.63	4559	4559	100.0	5.74	8.91	13.13	0.1027	0.0599
Inf - 0.63	12818	12818	100.0	9.20	22.61	34.27	0.0359	0.0217

Complete .cif-data of the compound are available under the CCDC number **CCDC-2063977**.

Table 9. Crystal data and structure refinement.

Identification code	13680	
Empirical formula	$C_{36} H_{26} Bi_2 Cl_4 O$	
Color	colourless	
Formula weight	1034.33 g · mol^{-1}	
Temperature	100(2) K	
Wavelength	0.71073 Å	
Crystal system	TRICLINIC	
Space group	*P*-1, (No. 2)	
Unit cell dimensions	a = 9.0281(4) Å	α= 101.289(2)°.
	b = 12.1880(5) Å	β= 90.246(2)°.
	c = 15.1195(6) Å	γ = 95.156(2)°.
Volume	1624.47(12) Å^3	
Z	2	
Density (calculated)	2.115 Mg · m^{-3}	
Absorption coefficient	11.176 mm^{-1}	
F(000)	968 e	
Crystal size	0.062 x 0.035 x 0.011 mm^3	
θ range for data collection	1.374 to 35.077°.	
Index ranges	$-14 \le h \le 14$, $-19 \le k \le 19$, $-24 \le l \le 24$	
Reflections collected	65647	
Independent reflections	14207 [R_{int} = 0.0403]	
Reflections with I>2σ(I)	11437	
Completeness to θ = 25.242°	100.0 %	
Absorption correction	Gaussian	
Max. and min. transmission	0.90 and 0.63	
Refinement method	Full-matrix least-squares on F^2	
Data / restraints / parameters	14207 / 0 / 388	
Goodness-of-fit on F^2	1.046	
Final R indices [I>2σ(I)]	R_1 = 0.0262	wR^2 = 0.0527
R indices (all data)	R_1 = 0.0408	wR^2 = 0.0566
Largest diff. peak and hole	2.0 and -1.4 e · Å^{-3}	

Table 10. Bond lengths [Å] and angles [°].

Bi(1)-Cl(1)	2.5816(6)	Bi(1)-Cl(2)	2.5827(6)
Bi(1)-C(2)	2.184(2)	Bi(1)-C(13)	2.213(2)
Bi(1)-C(19)	2.213(2)	Bi(2)-Cl(3)	2.5496(6)
Bi(2)-Cl(4)	2.5769(6)	Bi(2)-C(11)	2.186(3)
Bi(2)-C(25)	2.197(3)	Bi(2)-C(31)	2.194(2)
O(1)-C(1)	1.381(3)	O(1)-C(12)	1.384(3)
C(1)-C(2)	1.375(3)	C(1)-C(6)	1.403(3)
C(2)-C(3)	1.395(4)	C(3)-C(4)	1.398(4)
C(4)-C(5)	1.375(4)	C(5)-C(6)	1.394(4)
C(6)-C(7)	1.441(4)	C(7)-C(8)	1.398(4)
C(7)-C(12)	1.396(3)	C(8)-C(9)	1.376(4)
C(9)-C(10)	1.393(4)	C(10)-C(11)	1.397(4)
C(11)-C(12)	1.379(4)	C(13)-C(14)	1.375(4)
C(13)-C(18)	1.383(4)	C(14)-C(15)	1.383(4)
C(15)-C(16)	1.376(4)	C(16)-C(17)	1.368(4)
C(17)-C(18)	1.392(4)	C(19)-C(20)	1.374(4)
C(19)-C(24)	1.380(4)	C(20)-C(21)	1.389(4)
C(21)-C(22)	1.364(4)	C(22)-C(23)	1.382(4)
C(23)-C(24)	1.387(4)	C(25)-C(26)	1.376(4)
C(25)-C(30)	1.393(4)	C(26)-C(27)	1.385(4)
C(27)-C(28)	1.371(5)	C(28)-C(29)	1.377(5)
C(29)-C(30)	1.383(4)	C(31)-C(32)	1.383(3)
C(31)-C(36)	1.383(3)	C(32)-C(33)	1.387(4)
C(33)-C(34)	1.372(4)	C(34)-C(35)	1.379(4)
C(35)-C(36)	1.390(4)		
Cl(1)-Bi(1)-Cl(2)	177.46(2)	C(2)-Bi(1)-Cl(1)	86.64(7)
C(2)-Bi(1)-Cl(2)	91.17(7)	C(2)-Bi(1)-C(13)	130.24(9)
C(2)-Bi(1)-C(19)	113.08(9)	C(13)-Bi(1)-Cl(1)	89.26(7)
C(13)-Bi(1)-Cl(2)	91.19(7)	C(19)-Bi(1)-Cl(1)	90.29(7)
C(19)-Bi(1)-Cl(2)	91.75(7)	C(19)-Bi(1)-C(13)	116.51(8)
Cl(3)-Bi(2)-Cl(4)	174.32(2)	C(11)-Bi(2)-Cl(3)	92.41(7)
C(11)-Bi(2)-Cl(4)	87.53(7)	C(11)-Bi(2)-C(25)	112.86(9)
C(11)-Bi(2)-C(31)	128.63(9)	C(25)-Bi(2)-Cl(3)	93.52(7)

C(25)-Bi(2)-Cl(4)	91.74(7)	C(31)-Bi(2)-Cl(3)	88.73(6)
C(31)-Bi(2)-Cl(4)	86.88(6)	C(31)-Bi(2)-C(25)	118.32(9)
C(1)-O(1)-C(12)	104.98(18)	O(1)-C(1)-C(6)	111.9(2)
C(2)-C(1)-O(1)	126.3(2)	C(2)-C(1)-C(6)	121.7(2)
C(1)-C(2)-Bi(1)	124.53(17)	C(1)-C(2)-C(3)	118.3(2)
C(3)-C(2)-Bi(1)	116.85(18)	C(2)-C(3)-C(4)	120.2(2)
C(5)-C(4)-C(3)	121.4(2)	C(4)-C(5)-C(6)	118.7(2)
C(1)-C(6)-C(7)	105.3(2)	C(5)-C(6)-C(1)	119.6(2)
C(5)-C(6)-C(7)	135.0(2)	C(8)-C(7)-C(6)	134.4(2)
C(12)-C(7)-C(6)	105.9(2)	C(12)-C(7)-C(8)	119.7(2)
C(9)-C(8)-C(7)	118.4(2)	C(8)-C(9)-C(10)	121.8(3)
C(9)-C(10)-C(11)	120.1(3)	C(10)-C(11)-Bi(2)	115.9(2)
C(12)-C(11)-Bi(2)	126.00(18)	C(12)-C(11)-C(10)	118.1(2)
O(1)-C(12)-C(7)	111.9(2)	C(11)-C(12)-O(1)	126.2(2)
C(11)-C(12)-C(7)	121.9(2)	C(14)-C(13)-Bi(1)	120.16(19)
C(14)-C(13)-C(18)	122.4(2)	C(18)-C(13)-Bi(1)	117.36(19)
C(13)-C(14)-C(15)	118.2(3)	C(16)-C(15)-C(14)	120.6(3)
C(17)-C(16)-C(15)	120.4(3)	C(16)-C(17)-C(18)	120.4(3)
C(13)-C(18)-C(17)	118.0(3)	C(20)-C(19)-Bi(1)	118.46(19)
C(20)-C(19)-C(24)	121.2(2)	C(24)-C(19)-Bi(1)	120.36(18)
C(19)-C(20)-C(21)	119.2(3)	C(22)-C(21)-C(20)	120.4(3)
C(21)-C(22)-C(23)	120.1(3)	C(22)-C(23)-C(24)	120.3(3)
C(19)-C(24)-C(23)	118.8(3)	C(26)-C(25)-Bi(2)	118.36(19)
C(26)-C(25)-C(30)	121.9(3)	C(30)-C(25)-Bi(2)	119.72(19)
C(25)-C(26)-C(27)	118.3(3)	C(28)-C(27)-C(26)	120.9(3)
C(27)-C(28)-C(29)	120.1(3)	C(28)-C(29)-C(30)	120.6(3)
C(29)-C(30)-C(25)	118.2(3)	C(32)-C(31)-Bi(2)	116.25(17)
C(32)-C(31)-C(36)	122.8(2)	C(36)-C(31)-Bi(2)	120.90(18)
C(31)-C(32)-C(33)	118.3(2)	C(34)-C(33)-C(32)	120.2(2)
C(33)-C(34)-C(35)	120.3(3)	C(34)-C(35)-C(36)	121.1(3)
C(31)-C(36)-C(35)	117.1(2)		

Single crystal structure analysis of 10 (13220)

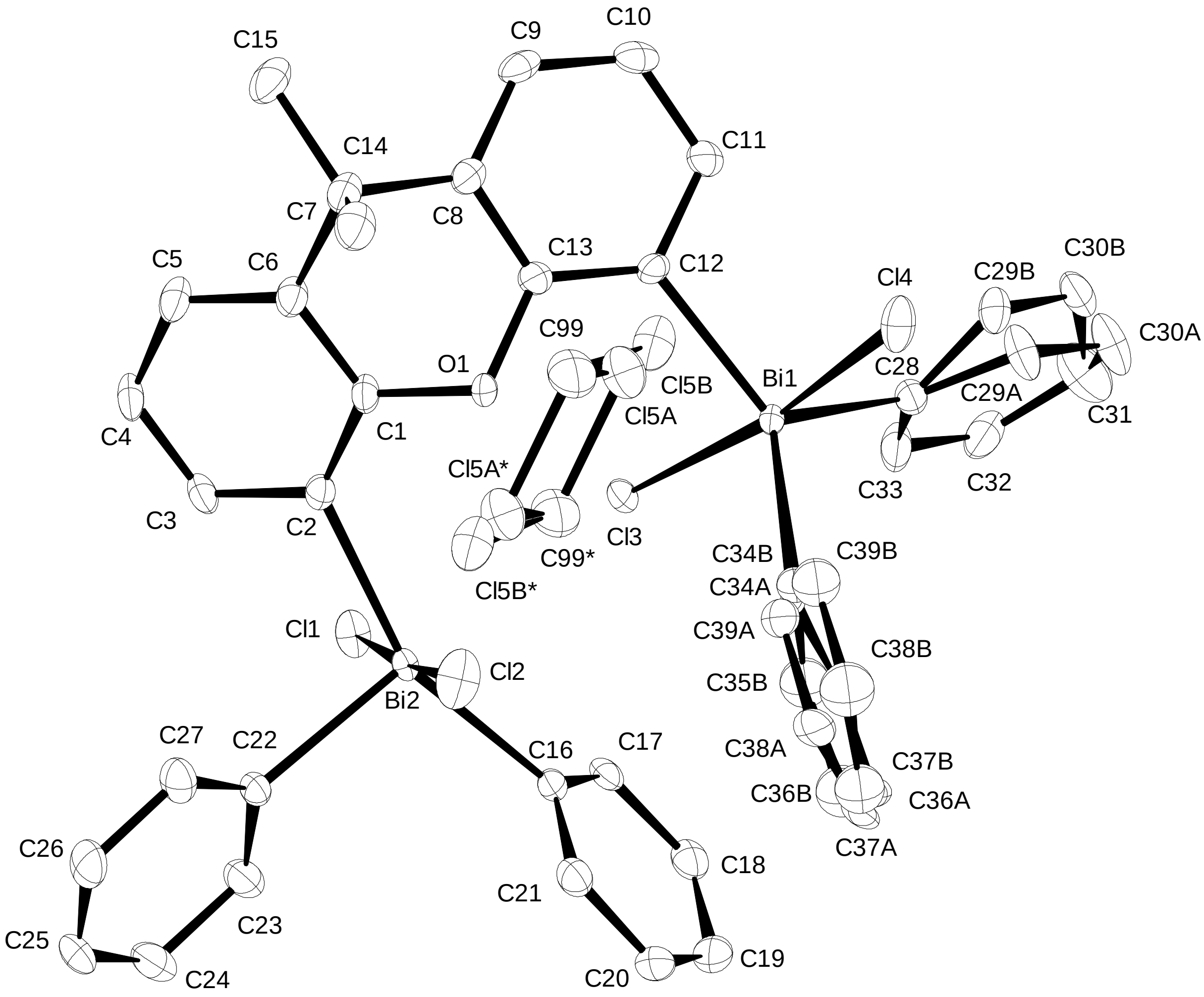

Figure 11. The molecular structure of complex **10**. H atoms have been removed for clarity.

X-ray Crystal Structure Analysis of complex 10: $C_{39.50}$ H_{33} Bi_2 Cl_5 O, M_r = 1118.87 g mol^{-1}, colourless prism, crystal size 0.052 x 0.026 x 0.021 mm^3, monoclinic, $P2_1/c$ [14], a = 12.3959(6) Å, b = 13.9993(6) Å, c = 21.7582(10) Å, β = 100.772(2) °, V = 3709.3(3) Å^3, T = 100(2) K, Z = 4, D_{calc} = 2.004 g·cm^3, λ = 0.71073 Å, $\mu(Mo\text{-}K_\alpha)$ = 9.867 mm^{-1}, Gaussian absorption correction ($T_{\min}$ = 0.67911, $T_{\max}$ = 0.83591), Bruker-AXS Mach3 diffractometer with APEX-II detector and IµS microfocus Mo-anode X-ray source, 1.672 < θ < 27.499 °, 144619 measured reflections, 8521 independent reflections, 7847 reflections with $I > 2\sigma(I)$, R_{int} = 0.0334. The structure was solved by *SHELXT* and refined by full-matrix least-squares (*SHELXL*) against F^2 to R_1 = 0.0160 [$I > 2\sigma(I)$], wR_2 = 0.0323, 492 parameters.

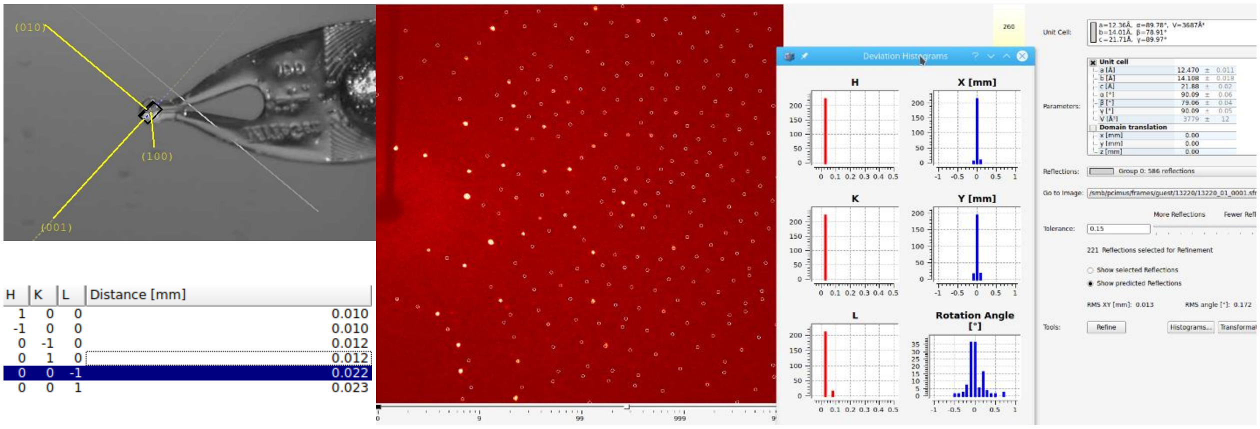

Figure 12. Crystal faces and unit cell determination of complex **10**.

INTENSITY STATISTICS FOR DATASET

Resolution	#Data	#Theory	%Complete	Redundancy	Mean I	Mean I/s	Rmerge	Rsigma
Inf - 2.69	231	231	100.0	19.55	106.93	109.89	0.0208	0.0065
2.69 - 1.77	538	538	100.0	23.22	92.12	113.70	0.0201	0.0062
1.77 - 1.39	786	786	100.0	23.81	57.52	96.90	0.0231	0.0070
1.39 - 1.21	790	790	100.0	23.76	44.42	86.92	0.0289	0.0080
1.21 - 1.10	744	744	100.0	22.34	38.58	74.03	0.0335	0.0093
1.10 - 1.02	784	784	100.0	17.83	31.29	57.27	0.0390	0.0120
1.02 - 0.96	748	748	100.0	15.43	29.79	49.87	0.0427	0.0140
0.96 - 0.91	808	808	100.0	13.74	22.93	40.46	0.0503	0.0177
0.91 - 0.87	750	750	100.0	12.55	20.43	35.44	0.0589	0.0210
0.87 - 0.83	926	926	100.0	11.95	17.78	30.48	0.0655	0.0246
0.83 - 0.80	821	821	100.0	11.48	18.04	29.28	0.0696	0.0263
0.80 - 0.78	591	591	100.0	11.11	15.30	24.73	0.0782	0.0308
0.78 - 0.76	712	712	100.0	10.80	14.98	22.97	0.0813	0.0330
0.76 - 0.74	744	744	100.0	10.54	13.30	21.03	0.0898	0.0375
0.74 - 0.72	842	842	100.0	10.16	11.08	17.75	0.1060	0.0459
0.72 - 0.70	944	944	100.0	9.84	11.55	16.91	0.1067	0.0472
0.70 - 0.68	1053	1053	100.0	9.52	10.40	15.18	0.1202	0.0542
0.68 - 0.67	577	577	100.0	9.06	8.99	13.07	0.1375	0.0646
0.67 - 0.66	599	599	100.0	9.14	8.59	12.25	0.1452	0.0681
0.66 - 0.65	667	667	100.0	8.81	7.40	10.75	0.1636	0.0804
0.65 - 0.64	694	725	95.7	7.08	7.54	9.74	0.1602	0.0960
0.74 - 0.64	5376	5407	99.4	9.17	9.61	14.08	0.1258	0.0604
Inf - 0.64	15349	15380	99.8	13.61	24.53	39.25	0.0413	0.0199

The structure contains a rotational disorder of 60:40 and 60:40 at phenyl ligands of Bi1. Disordered atoms have been partially refined isotropically. Additionally a solute molecule (DCM) is disordered about a crystallographic special position (inversion center) with 50:50 occupancy and the bond situation has been described using FREE instruction. The high residual electron density (highest peak: 2.45 at 0.66 Å from Bi1 and deepest hole: -3.00 at 0.72 Å from Bi1) could possibly be caused by anharmonic displacement of the Bi atom.

Complete .cif-data of the compound are available under the CCDC number **CCDC-2063980**.

Table 11. Crystal data and structure refinement.

Identification code	13220	
Empirical formula	$C_{39.50} H_{33} Bi_2 Cl_5 O$	
Color	colourless	
Formula weight	1118.87 g · mol^{-1}	
Temperature	100(2) K	
Wavelength	0.71073 Å	
Crystal system	MONOCLINIC	
Space group	$P2_1/c$, (No. 14)	
Unit cell dimensions	a = 12.3959(6) Å	α= 90°.
	b = 13.9993(6) Å	β= 100.772(2)°.
	c = 21.7582(10) Å	γ = 90°.
Volume	3709.3(3) Å^3	
Z	4	
Density (calculated)	2.004 Mg · m^{-3}	
Absorption coefficient	9.867 mm^{-1}	
F(000)	2116 e	
Crystal size	0.052 x 0.026 x 0.021 mm^3	
θ range for data collection	1.672 to 27.499°.	
Index ranges	-16 ≤ h ≤ 16, -18≤ k ≤ 18, -28 ≤ l ≤ 28	
Reflections collected	144619	
Independent reflections	8521 [R_{int} = 0.0334]	
Reflections with I>2σ(I)	7847	
Completeness to θ = 25.242°	100.0 %	
Absorption correction	Gaussian	
Max. and min. transmission	0.84 and 0.68	
Refinement method	Full-matrix least-squares on F^2	
Data / restraints / parameters	8521 / 0 / 492	
Goodness-of-fit on F^2	1.043	
Final R indices [I>2σ(I)]	R_1 = 0.0160	wR^2 = 0.0323
R indices (all data)	R_1 = 0.0196	wR^2 = 0.0339
Largest diff. peak and hole	2.5 and -3.0 e · Å^{-3}	

Table 12. Bond lengths [Å] and angles [°].

Bi(1)-Cl(3)	2.5828(7)	Bi(1)-Cl(4)	2.5825(7)
Bi(1)-C(12)	2.190(2)	Bi(1)-C(28)	2.214(3)
Bi(1)-C(34A)	2.189(5)	Bi(1)-C(34B)	2.258(10)
Bi(2)-Cl(1)	2.5977(6)	Bi(2)-Cl(2)	2.5702(7)
Bi(2)-C(2)	2.212(3)	Bi(2)-C(16)	2.223(2)
Bi(2)-C(22)	2.212(2)	O(1)-C(1)	1.391(3)
O(1)-C(13)	1.391(3)	C(1)-C(2)	1.388(4)
C(1)-C(6)	1.402(4)	C(2)-C(3)	1.394(3)
C(3)-C(4)	1.381(4)	C(4)-C(5)	1.386(4)
C(5)-C(6)	1.392(4)	C(6)-C(7)	1.521(4)
C(7)-C(8)	1.523(4)	C(7)-C(14)	1.541(4)
C(7)-C(15)	1.527(4)	C(8)-C(9)	1.389(4)
C(8)-C(13)	1.397(4)	C(9)-C(10)	1.388(4)
C(10)-C(11)	1.386(4)	C(11)-C(12)	1.388(4)
C(12)-C(13)	1.384(4)	C(16)-C(17)	1.380(4)
C(16)-C(21)	1.381(4)	C(17)-C(18)	1.391(4)
C(18)-C(19)	1.384(4)	C(19)-C(20)	1.385(4)
C(20)-C(21)	1.393(4)	C(22)-C(23)	1.379(4)
C(22)-C(27)	1.391(4)	C(23)-C(24)	1.394(4)
C(24)-C(25)	1.381(5)	C(25)-C(26)	1.377(5)
C(26)-C(27)	1.395(4)	C(28)-C(29A)	1.395(9)
C(28)-C(29B)	1.391(14)	C(28)-C(33)	1.379(4)
(29A)-C(30A)	1.377(11)	C(29B)-C(30B)	1.402(17)
C(30A)-C(31)	1.367(8)	C(30B)-C(31)	1.472(11)
C(31)-C(32)	1.365(4)	C(32)-C(33)	1.384(4)
C(34A)-C(35A)	1.377(8)	C(34A)-C(39A)	1.389(7)
C(34B)-C(35B)	1.364(13)	C(34B)-C(39B)	1.375(13)
C(35A)-C(36A)	1.391(6)	C(35B)-C(36B)	1.439(15)
C(36A)-C(37A)	1.398(8)	C(36B)-C(37B)	1.367(14)
C(37A)-C(38A)	1.376(8)	C(37B)-C(38B)	1.353(15)
C(38A)-C(39A)	1.398(7)	C(38B)-C(39B)	1.386(13)
Cl(5A)-C(99)	1.155(8)	Cl(5A)-C(99)*	1.769(7)
Cl(5B)-C(99)*	2.256(7)	Cl(5B)-C(99)	1.750(8)
C(99)-H(99A)	0.92(8)	C(99)-H(99B)	0.96(9)

Cl(4)-Bi(1)-Cl(3)	172.07(2)	C(12)-Bi(1)-Cl(3)	87.13(7)
C(12)-Bi(1)-Cl(4)	85.19(7)	C(12)-Bi(1)-C(28)	116.05(10)
C(12)-Bi(1)-C(34B)	129.8(3)	C(28)-Bi(1)-Cl(3)	91.72(7)
C(28)-Bi(1)-Cl(4)	93.43(7)	C(28)-Bi(1)-C(34B)	113.4(3)
C(34A)-Bi(1)-Cl(3)	90.43(15)	C(34A)-Bi(1)-Cl(4)	93.92(16)
C(34A)-Bi(1)-C(12)	137.74(16)	C(34A)-Bi(1)-C(28)	106.19(16)
C(34B)-Bi(1)-Cl(3)	100.0(2)	C(34B)-Bi(1)-Cl(4)	83.5(2)
Cl(2)-Bi(2)-Cl(1)	176.01(2)	C(2)-Bi(2)-Cl(1)	87.53(7)
C(2)-Bi(2)-Cl(2)	89.03(7)	C(2)-Bi(2)-C(16)	155.63(9)
C(2)-Bi(2)-C(22)	102.11(9)	C(16)-Bi(2)-Cl(1)	90.16(7)
C(16)-Bi(2)-Cl(2)	92.14(7)	C(22)-Bi(2)-Cl(1)	90.51(7)
C(22)-Bi(2)-Cl(2)	92.19(7)	C(22)-Bi(2)-C(16)	102.17(9)
C(13)-O(1)-C(1)	113.94(19)	O(1)-C(1)-C(6)	120.0(2)
C(2)-C(1)-O(1)	120.3(2)	C(2)-C(1)-C(6)	119.7(2)
C(1)-C(2)-Bi(2)	128.98(18)	C(1)-C(2)-C(3)	120.6(2)
C(3)-C(2)-Bi(2)	110.27(18)	C(4)-C(3)-C(2)	119.8(2)
C(3)-C(4)-C(5)	119.8(2)	C(4)-C(5)-C(6)	121.1(3)
C(1)-C(6)-C(7)	117.7(2)	C(5)-C(6)-C(1)	118.9(2)
C(5)-C(6)-C(7)	123.4(2)	C(6)-C(7)-C(8)	105.4(2)
C(6)-C(7)-C(14)	109.1(2)	C(6)-C(7)-C(15)	112.5(2)
C(8)-C(7)-C(14)	109.1(2)	C(8)-C(7)-C(15)	111.9(2)
C(15)-C(7)-C(14)	108.7(2)	C(9)-C(8)-C(7)	124.5(2)
C(9)-C(8)-C(13)	118.4(2)	C(13)-C(8)-C(7)	117.1(2)
C(10)-C(9)-C(8)	121.5(2)	C(11)-C(10)-C(9)	119.6(3)
C(10)-C(11)-C(12)	119.1(3)	C(11)-C(12)-Bi(1)	114.55(19)
C(13)-C(12)-Bi(1)	124.27(19)	C(13)-C(12)-C(11)	121.2(2)
O(1)-C(13)-C(8)	120.6(2)	C(12)-C(13)-O(1)	119.6(2)
C(12)-C(13)-C(8)	119.8(2)	C(17)-C(16)-Bi(2)	120.63(19)
C(17)-C(16)-C(21)	123.2(2)	C(21)-C(16)-Bi(2)	115.69(19)
C(16)-C(17)-C(18)	117.4(3)	C(19)-C(18)-C(17)	120.8(3)
C(18)-C(19)-C(20)	120.4(3)	C(19)-C(20)-C(21)	119.9(3)
C(16)-C(21)-C(20)	118.2(3)	C(23)-C(22)-Bi(2)	117.91(19)
C(23)-C(22)-C(27)	121.2(2)	C(27)-C(22)-Bi(2)	120.8(2)
C(22)-C(23)-C(24)	119.2(3)	C(25)-C(24)-C(23)	120.0(3)
C(26)-C(25)-C(24)	120.6(3)	C(25)-C(26)-C(27)	120.1(3)

C(22)-C(27)-C(26)	118.9(3)	C(29A)-C(28)-Bi(1)	121.5(4)
C(29B)-C(28)-Bi(1)	114.9(6)	C(33)-C(28)-Bi(1)	119.6(2)
C(33)-C(28)-C(29A)	117.8(4)	C(33)-C(28)-C(29B)	123.8(6)
C(28)-C(29A)-H(29A)	119.2	C(30A)-C(29A)-C(28)	121.6(7)
C(28)-C(29B)-C(30B)	113.4(10)	C(31)-C(30A)-C(29A)	118.2(6)
C(29B)-C(30B)-C(31)	122.6(9)	C(32)-C(31)-C(30A)	120.1(4)
C(32)-C(31)-C(30B)	115.6(5)	C(31)-C(32)-C(33)	120.4(3)
C(28)-C(33)-C(32)	119.5(3)	C(35A)-C(34A)-Bi(1)	117.6(4)
C(35A)-C(34A)-C(39A)	122.4(4)	C(39A)-C(34A)-Bi(1)	119.0(4)
C(35B)-C(34B)-Bi(1)	111.0(7)	C(35B)-C(34B)-C(39B)	124.9(9)
C(39B)-C(34B)-Bi(1)	123.1(7)	C(34A)-C(35A)-C(36A)	118.4(5)
C(34B)-C(35B)-C(36B)	117.3(10)	C(35A)-C(36A)-C(37A)	120.1(5)
C(37B)-C(36B)-C(35B)	117.3(10)	C(38A)-C(37A)-C(36A)	120.6(5)
C(38B)-C(37B)-C(36B)	123.1(11)	C(37A)-C(38A)-C(39A)	119.9(5)
C(37B)-C(38B)-C(39B)	121.1(10)	C(34A)-C(39A)-C(38A)	118.6(5)
C(34B)-C(39B)-C(38B)	116.2(10)	C(99)-Cl(5A)-C(99)*	78.0(5)
C(99)-Cl(5B)-C(99)*	54.9(4)	Cl(5A)-C(99)-Cl(5A)*	102.0(5)
Cl(5B)-C(99)-Cl(5A)*	112.6(4)	Cl(5B)-C(99)-H(99A)	109(5)
Cl(5B)-C(99)-H(99B)	111(5)	H(99A)-C(99)-H(99B)	110(7)

Symmetry transformations used to generate equivalent atoms: * -x+2,-y+2,-z+1

Single crystal structure analysis of 11 (13708)

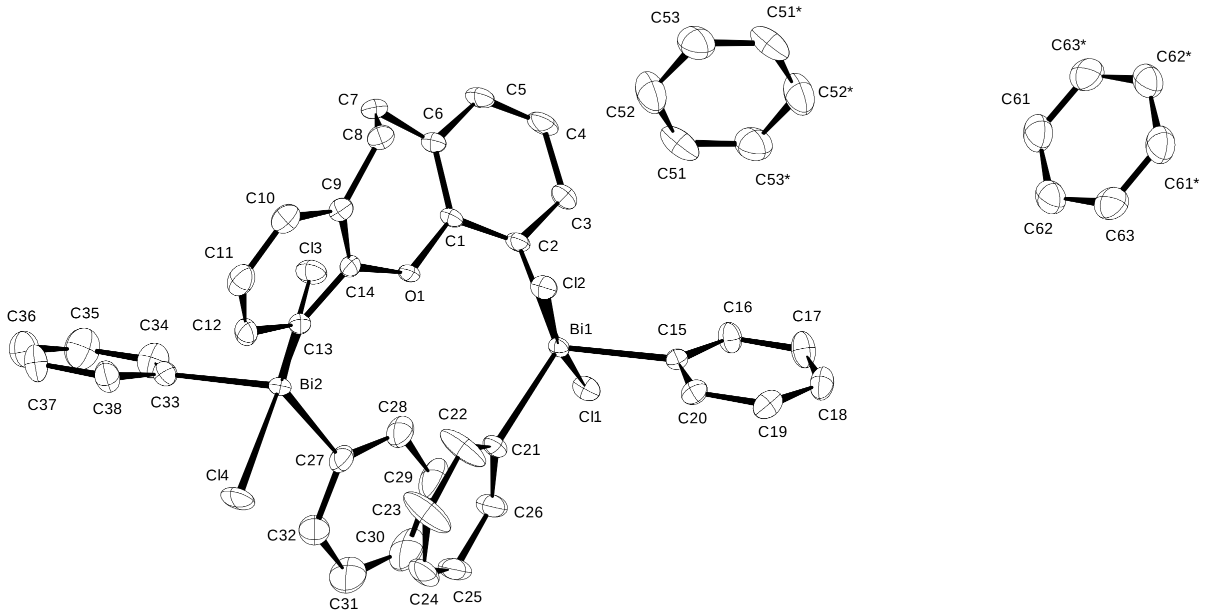

Figure 13. The molecular structure of complex **11**. H atoms have been removed for clarity.

X-ray Crystal Structure Analysis of complex 11: $C_{44}H_{36}Bi_2Cl_4O$, M_r = 1140.49 g mol^{-1}, yellow prism, crystal size 0.15 x 0.13 x 0.07 mm^3, triclinic, *P*-1 [2], a = 12.0198(3) Å, b = 12.1771(7) Å, c = 15.7796(11) Å, α = 96.190(5) °, β = 103.900(4) °, γ = 114.199(3) °, V = 1988.7(2) Å^3, T = 100(2) K, Z = 2, D_{calc} = 1.905 g·cm^3, λ = 0.71073 Å, $\mu(Mo\text{-}K_\alpha)$= 9.139 mm^{-1}, Gaussian absorption correction (T_{min} = 0.30255, T_{max} = 0.59321), Bruker AXS Enraf-Nonius KappaCCD diffractometer with a FR591 rotating Mo-anode X-ray source, 2.716 < θ < 30.508 °, 75349 measured reflections, 12134 independent reflections, 10803 reflections with $I > 2\sigma(I)$, R_{int} = 0.0444. The structure was solved by *SHELXS* and refined by full-matrix least-squares (*SHELXL*) against F^2 to R_1 = 0.0210 [$I > 2\sigma(I)$], wR_2 = 0.0436, 460 parameters.

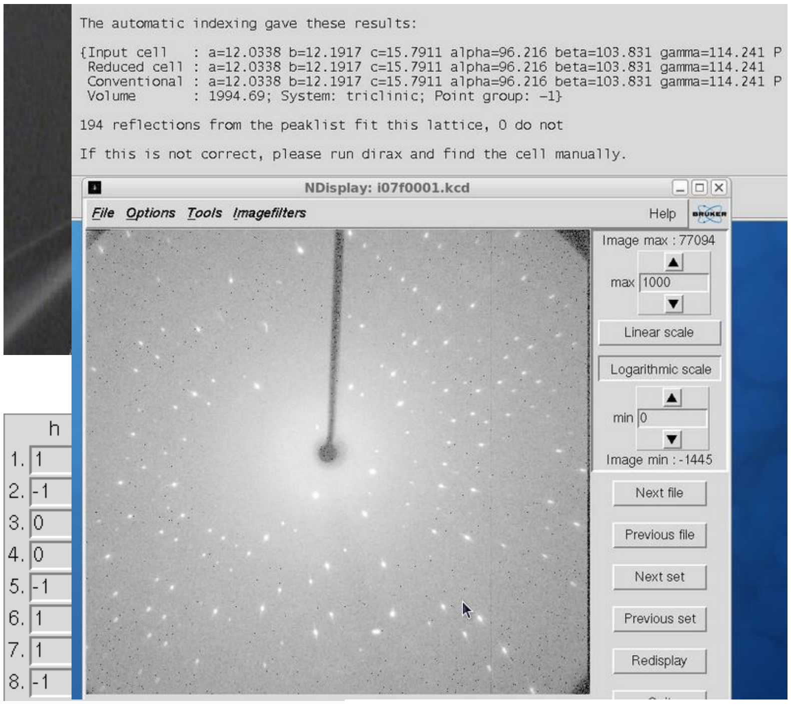

Figure 14. Crystal faces and unit cell determination of complex **11**.

INTENSITY STATISTICS FOR DATASET

Resolution	#Data	#Theory	%Complete	Redundancy	Mean I	Mean I/s	Rmerge	Rsigma
Inf - 2.32	326	335	97.3	7.26	132.92	52.70	0.0448	0.0163
2.32 - 1.56	763	763	100.0	7.62	95.60	47.40	0.0342	0.0166
1.56 - 1.24	1082	1082	100.0	7.50	62.64	39.99	0.0341	0.0181
1.24 - 1.08	1130	1130	100.0	7.27	47.51	36.25	0.0368	0.0201
1.08 - 0.98	1126	1126	100.0	6.89	36.41	30.62	0.0417	0.0228
0.98 - 0.91	1098	1098	100.0	6.47	28.86	27.10	0.0457	0.0263
0.91 - 0.86	1011	1011	100.0	6.15	24.12	23.50	0.0506	0.0300
0.86 - 0.81	1279	1279	100.0	5.80	20.66	20.40	0.0557	0.0344
0.81 - 0.78	947	947	100.0	5.52	18.15	18.56	0.0619	0.0389
0.78 - 0.75	1091	1091	100.0	5.28	15.08	15.78	0.0735	0.0454
0.75 - 0.72	1289	1289	100.0	5.05	14.01	14.83	0.0794	0.0509
0.72 - 0.70	992	992	100.0	4.84	11.26	12.27	0.0925	0.0623
0.70 - 0.68	1115	1115	100.0	4.61	10.57	11.32	0.1002	0.0688
0.68 - 0.66	1225	1225	100.0	4.48	8.63	9.44	0.1237	0.0863
0.66 - 0.64	1423	1423	100.0	4.21	7.55	7.95	0.1461	0.1079
0.64 - 0.63	729	729	100.0	4.15	7.54	7.43	0.1543	0.1176
0.63 - 0.62	842	842	100.0	4.01	7.00	6.68	0.1665	0.1369
0.62 - 0.60	1796	1796	100.0	3.86	5.60	4.96	0.2054	0.1933
0.60 - 0.59	1020	1020	100.0	3.68	4.73	3.67	0.2448	0.2715
0.59 - 0.58	1426	1474	96.7	3.44	4.54	3.11	0.2664	0.3321
0.68 - 0.58	8461	8509	99.4	3.95	6.39	6.03	0.1755	0.1652
Inf - 0.58	21710	21767	99.7	5.24	22.57	17.57	0.0540	0.0416

A resolution cut off (SHEL 99 0.7) was applied to suppress poorly measured intensities at higher diffraction angles.

Complete .cif-data of the compound are available under the CCDC number **CCDC-2063974**.

Table 13. Crystal data and structure refinement.

Identification code	13708	
Empirical formula	$C_{44} H_{36} Bi_2 Cl_4 O$	
Color	yellow	
Formula weight	1140.49 g · mol^{-1}	
Temperature	100(2) K	
Wavelength	0.71073 Å	
Crystal system	TRICLINIC	
Space group	*P*-1, (No. 2)	
Unit cell dimensions	a = 12.0198(3) Å	α= 96.190(5)°.
	b = 12.1771(7) Å	β= 103.900(4)°.
	c = 15.7796(11) Å	γ = 114.199(3)°.
Volume	1988.7(2) Å^3	
Z	2	
Density (calculated)	1.905 Mg · m^{-3}	
Absorption coefficient	9.139 mm^{-1}	
F(000)	1084 e	
Crystal size	0.15 x 0.13 x 0.07 mm^3	
θ range for data collection	2.716 to 30.508°.	
Index ranges	$-17 \leq h \leq 17$, $-17 \leq k \leq 17$, $-22 \leq l \leq 22$	
Reflections collected	75349	
Independent reflections	12134 [R_{int} = 0.0444]	
Reflections with $I>2\sigma(I)$	10803	
Completeness to θ = 25.242°	99.9 %	
Absorption correction	Gaussian	
Max. and min. transmission	0.59 and 0.30	
Refinement method	Full-matrix least-squares on F^2	
Data / restraints / parameters	12134 / 0 / 460	
Goodness-of-fit on F^2	1.072	
Final R indices [$I>2\sigma(I)$]	R_1 = 0.0210	wR^2 = 0.0436
R indices (all data)	R_1 = 0.0270	wR^2 = 0.0457
Largest diff. peak and hole	1.0 and -1.8 e · Å^{-3}	

Table 14. Bond lengths [Å] and angles [°].

Bi(1)-Cl(1)	2.5972(6)	Bi(1)-Cl(2)	2.5962(6)
Bi(1)-C(2)	2.192(2)	Bi(1)-C(15)	2.200(2)
Bi(1)-C(21)	2.212(2)	Bi(2)-Cl(3)	2.5881(7)
Bi(2)-Cl(4)	2.5856(7)	Bi(2)-C(13)	2.218(3)
Bi(2)-C(27)	2.216(3)	Bi(2)-C(33)	2.206(3)
O(1)-C(1)	1.381(3)	O(1)-C(14)	1.379(3)
C(1)-C(2)	1.386(3)	C(1)-C(6)	1.400(3)
C(2)-C(3)	1.386(3)	C(3)-C(4)	1.392(4)
C(4)-C(5)	1.380(4)	C(5)-C(6)	1.391(4)
C(6)-C(7)	1.502(4)	C(7)-C(8)	1.537(5)
C(8)-C(9)	1.499(4)	C(9)-C(10)	1.397(4)
C(9)-C(14)	1.401(4)	C(10)-C(11)	1.376(4)
C(11)-C(12)	1.383(4)	C(12)-C(13)	1.387(4)
C(13)-C(14)	1.392(3)	C(15)-C(16)	1.388(3)
C(15)-C(20)	1.386(3)	C(16)-C(17)	1.392(4)
C(17)-C(18)	1.388(4)	C(18)-C(19)	1.384(4)
C(19)-C(20)	1.389(4)	C(21)-C(22)	1.376(4)
C(21)-C(26)	1.373(4)	C(22)-C(23)	1.391(4)
C(23)-C(24)	1.375(5)	C(24)-C(25)	1.373(5)
C(25)-C(26)	1.393(4)	C(27)-C(28)	1.377(5)
C(27)-C(32)	1.387(4)	C(28)-C(29)	1.395(4)
C(29)-C(30)	1.387(6)	C(30)-C(31)	1.376(7)
C(31)-C(32)	1.391(5)	C(33)-C(34)	1.372(4)
C(33)-C(38)	1.389(4)	(34)-C(35)	1.387(5)
C(35)-C(36)	1.384(5)	C(36)-C(37)	1.382(5)
C(37)-C(38)	1.394(4)	C(51)-C(52)	1.381(5)
C(51)-C(53)*	1.364(5)	C(52)-C(53)	1.371(5)
C(61)-C(62)	1.372(5)	C(61)-C(63)**	1.388(5)
C(62)-C(63)	1.377(5)		
Cl(2)-Bi(1)-Cl(1)	170.725(19)	C(2)-Bi(1)-Cl(1)	88.91(7)
C(2)-Bi(1)-Cl(2)	85.48(7)	C(2)-Bi(1)-C(15)	119.38(9)
C(2)-Bi(1)-C(21)	127.71(9)	C(15)-Bi(1)-Cl(1)	88.26(6)
C(15)-Bi(1)-Cl(2)	88.06(6)	C(15)-Bi(1)-C(21)	112.80(9)

C(21)-Bi(1)-Cl(1)	96.08(7)	C(21)-Bi(1)-Cl(2)	93.19(7)
Cl(4)-Bi(2)-Cl(3)	175.59(2)	C(13)-Bi(2)-Cl(3)	91.39(7)
C(13)-Bi(2)-Cl(4)	89.29(7)	C(27)-Bi(2)-Cl(3)	90.69(9)
C(27)-Bi(2)-Cl(4)	91.67(9)	C(27)-Bi(2)-C(13)	138.46(10)
C(33)-Bi(2)-Cl(3)	87.62(7)	C(33)-Bi(2)-Cl(4)	88.07(7)
C(33)-Bi(2)-C(13)	110.94(10)	C(33)-Bi(2)-C(27)	110.60(11)
C(14)-O(1)-C(1)	127.87(19)	O(1)-C(1)-C(2)	113.8(2)
O(1)-C(1)-C(6)	126.8(2)	C(2)-C(1)-C(6)	119.4(2)
C(1)-C(2)-Bi(1)	116.30(17)	C(3)-C(2)-Bi(1)	119.50(19)
C(3)-C(2)-C(1)	122.6(2)	C(2)-C(3)-C(4)	117.8(2)
C(5)-C(4)-C(3)	119.4(2)	C(4)-C(5)-C(6)	123.1(3)
C(1)-C(6)-C(7)	121.7(2)	C(5)-C(6)-C(1)	117.1(3)
C(5)-C(6)-C(7)	120.5(2)	C(6)-C(7)-C(8)	110.1(2)
C(9)-C(8)-C(7)	109.6(2)	C(10)-C(9)-C(8)	120.7(2)
C(10)-C(9)-C(14)	117.3(2)	C(14)-C(9)-C(8)	121.4(2)
C(11)-C(10)-C(9)	122.8(3)	C(10)-C(11)-C(12)	119.3(3)
C(11)-C(12)-C(13)	118.9(3)	C(12)-C(13)-Bi(2)	114.97(19)
C(12)-C(13)-C(14)	121.7(2)	C(14)-C(13)-Bi(2)	121.05(19)
O(1)-C(14)-C(9)	125.4(2)	O(1)-C(14)-C(13)	115.2(2)
C(13)-C(14)-C(9)	119.3(2)	C(16)-C(15)-Bi(1)	121.55(19)
C(20)-C(15)-Bi(1)	116.02(17)	C(20)-C(15)-C(16)	122.4(2)
C(15)-C(16)-C(17)	118.0(3)	C(18)-C(17)-C(16)	120.5(3)
C(19)-C(18)-C(17)	120.4(3)	C(18)-C(19)-C(20)	120.2(3)
C(15)-C(20)-C(19)	118.6(2)	C(22)-C(21)-Bi(1)	118.0(2)
C(26)-C(21)-Bi(1)	119.80(18)	C(26)-C(21)-C(22)	122.1(3)
C(21)-C(22)-C(23)	118.7(3)	C(24)-C(23)-C(22)	120.3(3)
C(25)-C(24)-C(23)	119.9(3)	C(24)-C(25)-C(26)	120.9(3)
C(21)-C(26)-C(25)	118.1(3)	C(28)-C(27)-Bi(2)	120.7(2)
C(28)-C(27)-C(32)	123.0(3)	C(32)-C(27)-Bi(2)	116.1(3)
C(27)-C(28)-C(29)	118.0(3)	C(30)-C(29)-C(28)	120.3(4)
C(31)-C(30)-C(29)	120.2(3)	C(30)-C(31)-C(32)	120.8(4)
C(27)-C(32)-C(31)	117.7(4)	C(34)-C(33)-Bi(2)	117.5(2)
C(34)-C(33)-C(38)	121.3(3)	C(38)-C(33)-Bi(2)	121.1(2)
C(33)-C(34)-C(35)	119.5(3)	C(36)-C(35)-C(34)	120.0(3)
C(37)-C(36)-C(35)	120.4(3)	C(36)-C(37)-C(38)	119.9(3)
C(33)-C(38)-C(37)	118.9(3)	C(53)*-C(51)-C(52)	120.1(3)

C(53)-C(52)-C(51)	119.7(3)	C(51)*-C(53)-C(52)	120.2(3)
C(62)-C(61)-C(63)**	120.0(4)	C(61)-C(62)-C(63)	120.4(3)
C(62)-C(63)-C(61)**	119.6(4)		

Symmetry transformations used to generate equivalent atoms:

* -x,-y+1,-z+1   ** -x,-y+2,-z

Single crystal structure analysis of 12 (13386)

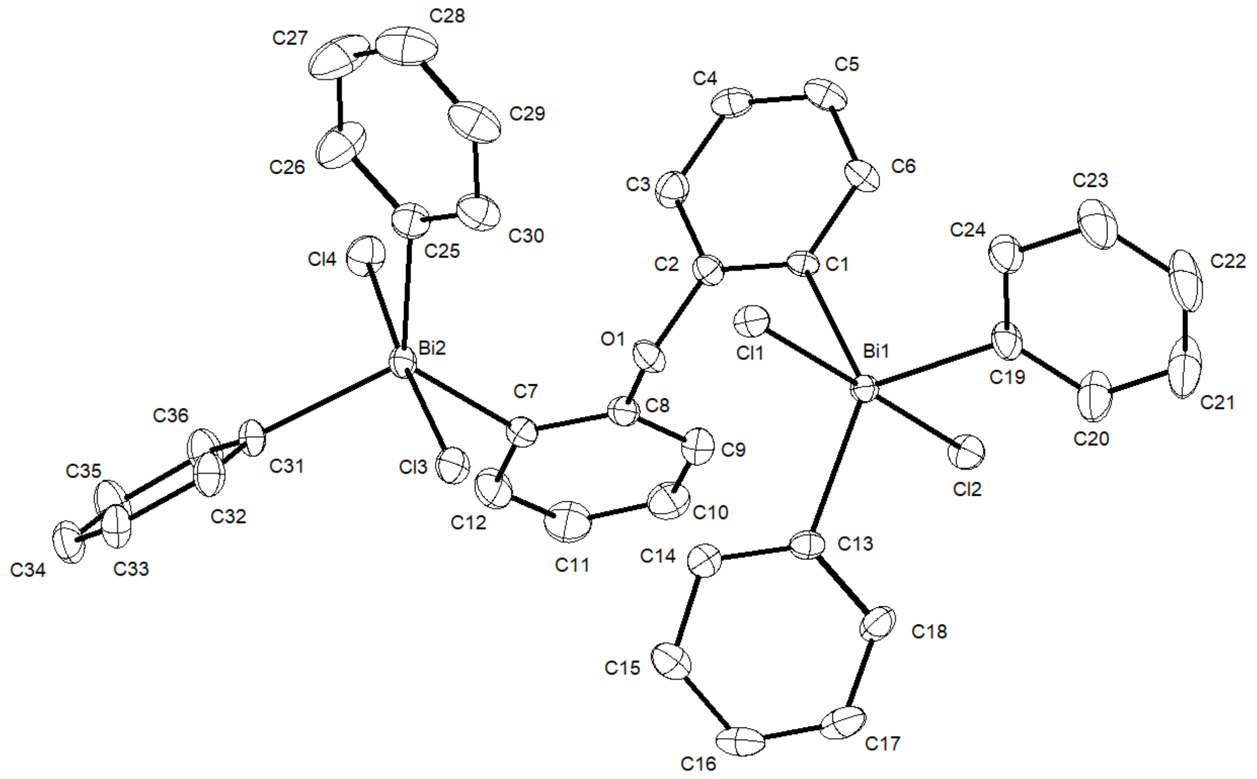

Figure 15. The molecular structure of complex **12**. H atoms have been removed for clarity.

X-ray Crystal Structure Analysis of complex 12: C_{36} H_{28} Bi_2 Cl_4 O, M_r = 1036.34 g mol^{-1}, colourless prism, crystal size 0.046 x 0.024 x 0.022 mm^3, monoclinic, $P2_1/n$ [14], a = 8.4334(5) Å, b = 25.9105(15) Å, c = 15.5944(9) Å, β = 91.060(2) °, V = 3407.0(3) Å^3, T = 100(2) K, Z = 4, D_{calc} = 2.020 g·cm^3, λ = 0.71073 Å, $\mu(Mo\text{-}K_\alpha)$= 10.658 mm^{-1}, Gaussian absorption correction ($T_{\min}$ = 0.67881, $T_{\max}$ = 0.85075), Bruker-AXS Mach3 diffractometer with APEX-II detector and IµS microfocus Mo-anode X-ray source, 1.524 < θ < 30.508 °, 151031 measured reflections, 10382 independent reflections, 9085 reflections with $I > 2\sigma(I)$, R_{int} = 0.0595]. The structure was solved by *SHELXT* and refined by full-matrix least-squares (*SHELXL*) against F^2 to R_1 = 0.0253 [$I > 2\sigma(I)$], wR_2 = 0.0521, 388 parameters.

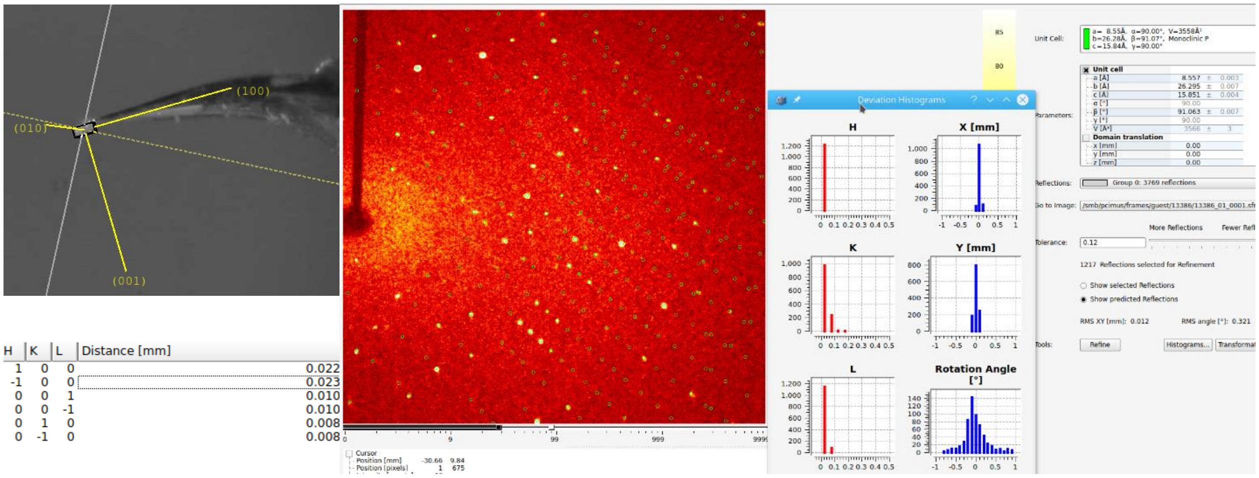

Figure 16. Crystal faces and unit cell determination of complex **12**.

INTENSITY STATISTICS FOR DATASET

Resolution	#Data	#Theory	%Complete	Redundancy	Mean I	Mean I/s	Rmerge	Rsigma
Inf - 2.45	267	267	100.0	20.20	103.09	56.90	0.0462	0.0144
2.45 - 1.62	616	616	100.0	23.06	72.24	58.09	0.0399	0.0137
1.62 - 1.28	895	895	100.0	23.17	46.06	51.39	0.0389	0.0146
1.28 - 1.11	924	924	100.0	22.10	35.78	43.86	0.0466	0.0162
1.11 - 1.01	877	877	100.0	17.24	26.65	32.97	0.0588	0.0210
1.01 - 0.93	981	981	100.0	14.04	22.64	27.09	0.0690	0.0262
0.93 - 0.88	830	830	100.0	12.06	17.49	21.36	0.0862	0.0333
0.88 - 0.83	1012	1012	100.0	11.32	16.19	18.62	0.0963	0.0377
0.83 - 0.80	755	755	100.0	10.72	13.84	16.33	0.1110	0.0444
0.80 - 0.77	834	834	100.0	10.42	13.17	15.62	0.1263	0.0490
0.77 - 0.74	1021	1021	100.0	9.80	10.77	12.97	0.1454	0.0598
0.74 - 0.72	768	768	100.0	9.54	10.42	12.18	0.1553	0.0646
0.72 - 0.70	832	832	100.0	9.15	8.45	9.88	0.1810	0.0805
0.70 - 0.68	985	985	100.0	8.90	7.63	8.96	0.2039	0.0909
0.68 - 0.66	1071	1071	100.0	8.51	7.31	8.15	0.2203	0.1012
0.66 - 0.65	563	563	100.0	8.32	5.96	6.93	0.2573	0.1267
0.65 - 0.63	1322	1322	100.0	7.90	5.42	6.03	0.2729	0.1441
0.63 - 0.62	725	725	100.0	7.75	5.22	5.68	0.2990	0.1563
0.62 - 0.61	750	750	100.0	7.42	4.74	5.04	0.3239	0.1810
0.61 - 0.60	781	781	100.0	7.17	4.39	4.47	0.3471	0.2085
0.60 - 0.59	834	1025	81.4	3.23	3.14	2.26	0.4051	0.4727
0.69 - 0.59	6537	6728	97.2	7.23	5.43	5.83	0.2715	0.1631
Inf - 0.59	17643	17834	98.9	11.55	17.54	18.61	0.0711	0.0426

A resolution cut off (SHEL 99 0.7) was applied to suppress poorly measured intensities at higher diffraction angles. The high residual electron density (highest peak: 3.13 at 0.78 Å from Bi1 and deepest hole: -1.09 at 1.28 Å from Bi1) could possibly be caused by anharmonic displacement of the Bi atom.

Complete .cif-data of the compound are available under the CCDC number **CCDC-2063979**.

Table 15. Crystal data and structure refinement.

Identification code	13386	
Empirical formula	$C_{36} H_{28} Bi_2 Cl_4 O$	
Color	colourless	
Formula weight	1036.34 g·mol^{-1}	
Temperature	100(2) K	
Wavelength	0.71073 Å	
Crystal system	Monoclinic	
Space group	*P* 2_1/n, (No. 14)	
Unit cell dimensions	a = 8.4334(5) Å	α= 90°.
	b = 25.9105(15) Å	β= 91.060(2)°.
	c = 15.5944(9) Å	γ = 90°.
Volume	3407.0(3) Å^3	
Z	4	
Density (calculated)	2.020 Mg·m^{-3}	
Absorption coefficient	10.658 mm^{-1}	
F(000)	1944 e	
Crystal size	0.046 x 0.024 x 0.022 mm^3	
θ range for data collection	1.524 to 30.508°.	
Index ranges	$-12 \le h \le 12$, $-37\le k \le 37$, $-22 \le l \le 22$	
Reflections collected	151031	
Independent reflections	10382 [R_{int} = 0.0595]	
Reflections with I>2σ(I)	9085	
Completeness to θ = 25.242°	100.0 %	
Absorption correction	Gaussian	
Max. and min. transmission	0.85075 and 0.67881	
Refinement method	Full-matrix least-squares on F^2	
Data / restraints / parameters	10382 / 0 / 388	
Goodness-of-fit on F^2	1.089	
Final R indices [I>2σ(I)]	R_1 = 0.0253	wR^2 = 0.0521
R indices (all data)	R_1 = 0.0327	wR^2 = 0.0542
Extinction coefficient	n/a	
Largest diff. peak and hole	3.134 and -1.090 e·Å^{-3}	

Table 16. Bond lengths [Å] and angles [°].

Bi(1)-Cl(2)	2.5892(8)	Bi(1)-Cl(1)	2.5862(8)
Bi(1)-C(13)	2.199(3)	Bi(1)-C(1)	2.184(3)
Bi(1)-C(19)	2.204(3)	Bi(2)-Cl(4)	2.6191(8)
Bi(2)-Cl(3)	2.5677(8)	Bi(2)-C(7)	2.189(3)
Bi(2)-C(31)	2.214(3)	Bi(2)-C(25)	2.205(3)
O(1)-C(2)	1.399(4)	O(1)-C(8)	1.396(4)
C(33)-H(33)	0.9500	C(33)-C(32)	1.391(5)
C(33)-C(34)	1.380(5)	C(7)-C(8)	1.385(4)
C(7)-C(12)	1.388(5)	C(4)-H(4)	0.9500
C(4)-C(3)	1.386(5)	C(4)-C(5)	1.383(5)
C(13)-C(18)	1.385(4)	C(13)-C(14)	1.380(4)
C(2)-C(1)	1.377(4)	C(2)-C(3)	1.392(4)
C(1)-C(6)	1.389(4)	C(3)-H(3)	0.9500
C(19)-C(24)	1.392(4)	C(19)-C(20)	1.382(5)
C(5)-H(5)	0.9500	C(5)-C(6)	1.382(5)
C(17)-H(17)	0.9500	C(17)-C(18)	1.390(5)
C(17)-C(16)	1.377(5)	C(8)-C(9)	1.384(5)
C(24)-H(24)	0.9500	C(24)-C(23)	1.393(5)
C(15)-H(15)	0.9500	C(15)-C(16)	1.386(5)
C(15)-C(14)	1.394(5)	C(32)-H(32)	0.9500
C(32)-C(31)	1.380(5)	C(34)-H(34)	0.9500
C(34)-C(35)	1.379(5)	C(31)-C(36)	1.387(4)
C(18)-H(18)	0.9500	C(36)-H(36)	0.9500
C(36)-C(35)	1.396(5)	C(16)-H(16)	0.9500
C(25)-C(30)	1.375(5)	C(25)-C(26)	1.385(5)
C(9)-H(9)	0.9500	C(9)-C(10)	1.383(5)
C(14)-H(14)	0.9500	C(35)-H(35)	0.9500
C(23)-H(23)	0.9500	C(23)-C(22)	1.384(6)
C(6)-H(6)	0.9500	C(30)-H(30)	0.9500
C(30)-C(29)	1.404(5)	C(11)-H(11)	0.9500
C(11)-C(12)	1.382(6)	C(11)-C(10)	1.381(6)
C(12)-H(12)	0.9500	C(10)-H(10)	0.9500
C(28)-H(28)	0.9500	C(28)-C(29)	1.366(6)
C(28)-C(27)	1.375(6)	C(20)-H(20)	0.9500

C(20)-C(21)	1.392(5)	C(29)-H(29)	0.9500
C(22)-H(22)	0.9500	C(22)-C(21)	1.384(6)
C(26)-H(26)	0.9500	C(26)-C(27)	1.390(6)
C(21)-H(21)	0.9500	C(27)-H(27)	0.9500

Cl(1)-Bi(1)-Cl(2)	175.42(3)	C(13)-Bi(1)-Cl(2)	88.59(8)
C(13)-Bi(1)-Cl(1)	92.04(8)	C(13)-Bi(1)-C(19)	120.09(12)
C(1)-Bi(1)-Cl(2)	90.01(8)	C(1)-Bi(1)-Cl(1)	86.12(8)
C(1)-Bi(1)-C(13)	130.36(11)	C(1)-Bi(1)-C(19)	109.56(12)
C(19)-Bi(1)-Cl(2)	91.13(9)	C(19)-Bi(1)-Cl(1)	92.47(9)
Cl(3)-Bi(2)-Cl(4)	176.06(3)	C(7)-Bi(2)-Cl(4)	83.75(9)
C(7)-Bi(2)-Cl(3)	93.06(9)	C(7)-Bi(2)-C(31)	114.23(12)
C(7)-Bi(2)-C(25)	128.79(12)	C(31)-Bi(2)-Cl(4)	91.96(9)
C(31)-Bi(2)-Cl(3)	91.45(8)	C(25)-Bi(2)-Cl(4)	90.22(9)
C(25)-Bi(2)-Cl(3)	89.99(9)	C(25)-Bi(2)-C(31)	116.77(12)
C(8)-O(1)-C(2)	115.0(2)	C(32)-C(33)-H(33)	119.9
C(34)-C(33)-H(33)	119.9	C(34)-C(33)-C(32)	120.3(3)
C(8)-C(7)-Bi(2)	120.0(2)	C(8)-C(7)-C(12)	120.5(3)
C(12)-C(7)-Bi(2)	119.2(2)	C(3)-C(4)-H(4)	119.4
C(5)-C(4)-H(4)	119.4	C(5)-C(4)-C(3)	121.1(3)
C(18)-C(13)-Bi(1)	117.5(2)	C(14)-C(13)-Bi(1)	119.6(2)
C(14)-C(13)-C(18)	122.9(3)	C(1)-C(2)-O(1)	118.7(3)
C(1)-C(2)-C(3)	120.1(3)	C(3)-C(2)-O(1)	121.1(3)
C(2)-C(1)-Bi(1)	121.2(2)	C(2)-C(1)-C(6)	121.2(3)
C(6)-C(1)-Bi(1)	117.6(2)	C(4)-C(3)-C(2)	118.6(3)
C(4)-C(3)-H(3)	120.7	C(2)-C(3)-H(3)	120.7
C(24)-C(19)-Bi(1)	119.3(2)	C(20)-C(19)-Bi(1)	118.5(2)
C(20)-C(19)-C(24)	122.2(3)	C(4)-C(5)-H(5)	119.9
C(6)-C(5)-C(4)	120.2(3)	C(6)-C(5)-H(5)	119.9
C(18)-C(17)-H(17)	119.8	C(16)-C(17)-H(17)	119.8
C(16)-C(17)-C(18)	120.4(3)	C(7)-C(8)-O(1)	118.7(3)
C(9)-C(8)-O(1)	121.2(3)	C(9)-C(8)-C(7)	120.1(3)
C(19)-C(24)-H(24)	121.0	C(19)-C(24)-C(23)	118.0(3)
C(23)-C(24)-H(24)	121.0	C(16)-C(15)-H(15)	119.7
C(16)-C(15)-C(14)	120.6(3)	C(14)-C(15)-H(15)	119.7
C(33)-C(32)-H(32)	120.4	C(31)-C(32)-C(33)	119.1(3)

C(31)-C(32)-H(32)	120.4
C(35)-C(34)-C(33)	120.0(3)
C(32)-C(31)-Bi(2)	118.3(2)
C(36)-C(31)-Bi(2)	120.0(2)
C(13)-C(18)-H(18)	120.9
C(31)-C(36)-H(36)	120.9
C(35)-C(36)-H(36)	120.9
C(17)-C(16)-H(16)	119.8
C(30)-C(25)-Bi(2)	120.7(3)
C(26)-C(25)-Bi(2)	116.5(3)
C(10)-C(9)-C(8)	119.1(3)
C(13)-C(14)-C(15)	117.6(3)
C(15)-C(14)-H(14)	121.2
C(34)-C(35)-H(35)	119.6
C(24)-C(23)-H(23)	119.8
C(22)-C(23)-H(23)	119.8
C(5)-C(6)-C(1)	118.8(3)
C(25)-C(30)-H(30)	121.2
C(29)-C(30)-H(30)	121.2
C(10)-C(11)-H(11)	120.0
C(7)-C(12)-H(12)	120.4
C(11)-C(12)-H(12)	120.4
C(11)-C(10)-C(9)	121.0(4)
C(29)-C(28)-H(28)	119.7
C(27)-C(28)-H(28)	119.7
C(19)-C(20)-C(21)	118.7(4)
C(30)-C(29)-H(29)	119.6
C(28)-C(29)-H(29)	119.6
C(21)-C(22)-C(23)	120.5(4)
C(25)-C(26)-H(26)	121.0
C(27)-C(26)-H(26)	121.0
C(22)-C(21)-C(20)	120.0(4)
C(28)-C(27)-C(26)	120.4(4)
C(26)-C(27)-H(27)	119.8
C(33)-C(34)-H(34)	120.0
C(35)-C(34)-H(34)	120.0
C(32)-C(31)-C(36)	121.6(3)
C(13)-C(18)-C(17)	118.1(3)
C(17)-C(18)-H(18)	120.9
C(31)-C(36)-C(35)	118.2(3)
C(17)-C(16)-C(15)	120.3(3)
C(15)-C(16)-H(16)	119.8
C(30)-C(25)-C(26)	122.7(3)
C(8)-C(9)-H(9)	120.5
C(10)-C(9)-H(9)	120.5
C(13)-C(14)-H(14)	121.2
C(34)-C(35)-C(36)	120.7(3)
C(36)-C(35)-H(35)	119.6
C(22)-C(23)-C(24)	120.4(3)
C(1)-C(6)-H(6)	120.6
C(5)-C(6)-H(6)	120.6
C(25)-C(30)-C(29)	117.5(4)
C(12)-C(11)-H(11)	120.0
C(10)-C(11)-C(12)	120.0(3)
C(11)-C(12)-C(7)	119.3(3)
C(9)-C(10)-H(10)	119.5
C(11)-C(10)-H(10)	119.5
C(29)-C(28)-C(27)	120.5(4)
C(19)-C(20)-H(20)	120.7
C(21)-C(20)-H(20)	120.7
C(28)-C(29)-C(30)	120.8(4)
C(23)-C(22)-H(22)	119.7
C(21)-C(22)-H(22)	119.7
C(25)-C(26)-C(27)	118.1(4)
C(20)-C(21)-H(21)	120.0
C(22)-C(21)-H(21)	120.0
C(28)-C(27)-H(27)	119.8
